# A revision of “blanket-hermit crabs” of the genus *Paguropsis* Henderson, 1888, with the description of a new genus and five new species (Crustacea, Anomura, Diogenidae)

**DOI:** 10.3897/zookeys.752.23712

**Published:** 2018-04-23

**Authors:** Rafael Lemaitre, Dwi Listyo Rahayu, Tomoyuki Komai

**Affiliations:** 1 Department of Invertebrate Zoology, National Museum of Natural History, Smithsonian Institution, 4210 Silver Hill Road, Suitland, MD 20746, USA; 2 Marine Bio-Industry Technical Implementation Unit, Mataram, Research Center for Oceanography-Indonesian Institute of Sciences (LIPI), Teluk Kodek, Pemenang, Lombok Barat, NTB, Indonesia; 3 Natural History Museum and Institute, Chiba, 955–2 Aoba-cho, Chuo-ku, Chiba, 260–8682 Japan

**Keywords:** Diogenidae, hermit crab, new species, *Paguropsina* gen n. *Paguropsis*, symbiotic anemone

## Abstract

For 130 years the diogenid genus *Paguropsis* Henderson, 1888 was considered monotypic for an unusual species, *P. typica* Henderson, 1888, described from the Philippines and seldom reported since. Although scantly studied, this species is known to live in striking symbiosis with a colonial sea anemone that the hermit can stretch back and forth like a blanket over its cephalic shield and part of cephalothoracic appendages, and thus the common name “blanket-crab”. During a study of paguroid collections obtained during recent French-sponsored biodiversity campaigns in the Indo-West Pacific, numerous specimens assignable to *Paguropsis* were encountered. Analysis and comparison with types and other historical specimens deposited in various museums revealed the existence of five undescribed species. Discovery of these new species, together with the observation of anatomical characters previously undocumented or poorly described, including coloration, required a revision of the genus *Paguropsis*. The name *Chlaenopagurus andersoni* Alcock & McArdle, 1901, considered by Alcock (1905) a junior synonym of *P. typica*, proved to be a valid species and is resurrected as *P. andersoni* (Alcock, 1899). In two of the new species, the shape of the gills, length/width of exopod of maxilliped 3, width and shape of sternite XI (of pereopods 3), and armature of the dactyls and fixed fingers of the chelate pereopods 4, were found to be characters so markedly different from *P. typica* and other species discovered that a new genus for them, *Paguropsina* **gen. n.**, is justified. As result, the genus *Paguropsis* is found to contain five species: *P. typica*, *P. andersoni*, *P. confusa* **sp. n.**, *P. gigas* **sp. n.**, and *P. lacinia* **sp. n.** Herein, *Paguropsina* **gen. n.**, is proposed and diagnosed for two new species, *P. pistillata* **gen. et sp. n.**, and *P. inermis* **gen. et sp. n.**; *Paguropsis* is redefined, *P. typica* and its previously believed junior synonym, *P. andersoni*, are redescribed. All species are illustrated, and color photographs provided. Also included are a summary of the biogeography of the two genera and all species; remarks on the significance of the unusual morphology; and remarks on knowledge of the symbiotic anemones used by the species. To complement the morphological descriptions and assist in future population and phylogenetic investigations, molecular data for mitochondrial COI barcode region and partial sequences of 12S and 16S rRNA are reported. A preliminary phylogenetic analysis using molecular data distinctly shows support for the separation of the species into two clades, one with all five species of *Paguropsis*, and another with the two species *Paguropsina* **gen. n.**

“*In the case of Paguropsis typica the association with a colonial sea-anemone of a genus related to Mamillifera is even more remarkable. Here there is no shell to play the part of “Sir Pandarus of Troy,” but the sea-anemone settles upon the hinder part of the young hermit-crab’s tail, and the two animals grow up together, in such a way that the spreading zoophytes form a blanket which the hermit can either draw completely forwards over its head or throw half-back, as it pleases.*”

(Alcock 1905: 7)

## Introduction

Two unusual hermit crab specimens collected in the Sibuyan Sea, the Philippines, on board the HMS *Challenger* Expedition in 1874, were considered so unique by Henderson (1888) that he named a new genus and new species for them, *Paguropsis typicus* Henderson, 1888 [the spelling of the specific epithet was subsequently corrected by Alcock (1905) to *P. typica*, for gender agreement]. Henderson found the morphology of the specimens, a male and a female, to be peculiar among hermit crabs, in particular the subdorsal position of pereopods 4 and 5, the presence of unpaired pleopods on the right side rather than on the left, and the membranous pleon bent on itself rather than spirally curved. He was intrigued by the lack of pleonal curvature, and that no trace of habitation or pleonal protection was found with the specimens. Apparently unaware of Henderson’s (1888) taxon, Alcock (1899) described, and Alcock and McArdle (1901) later illustrated, a new genus and species of a hermit crab based on hundreds of specimens collected off southern India, which he named *Chlaenopagurus andersoni* Alcock, 1899. Alcock also noted various unusual characters such as the puzzling general symmetry, despite the variability of pleopods 3-5 which in his specimens could be present on either the left or right sides in either sex, and devoted considerable discussion to the commensalism he observed. He was able to determine that his species did not use a shell for housing but instead was adapted to live with a colonial sea anemone with a sheet-like coenosarc that the hermit crab tucked under its telson and could stretch over its back like a blanket, thus the common name “blanket-crab” he used in the narratives while on board the “*Investigator*” expeditions (Alcock 1902). Subsequently, Alcock (1905) considered his taxon to be the same as Henderson’s, and formally placed his *C. andersoni* as a junior synonym of *P. typica*.

*Paguropsis* Henderson, 1888 has remained a monotypic genus since its original description. The single representative of this genus, *P. typica*, has been presumed to have a broad distribution across the tropical Indo-West-Pacific, from the Philippines, Japan, and Indonesia, to off eastern Africa, where adults have been reported in depths ranging from 110 to 350 m (Henderson 1888, Alcock 1899, Thompson 1943, Kamalaveni 1950, Barnard 1962, Miyake 1982, Baba et al. 1986, Thomas 1989). *Paguropsis typica* has often been used as an example of a remarkable adaptive specialization and diversity in the Anomura (Stebbing 1893, Boas 1926, Russell 1962, Nicol 1967, Kaestner 1970, Schäfer et al. 1983, McLaughlin 2015) and considerable attention has been given to the curious symbiotic association of this hermit crab with its cnidarian symbiont. However, much confusion exists on the identity of the symbiont as various names and misspellings have been used, such as *Epizoanthus paguropsidis* or *E. paguropsides*, by Boas (1926), Schäfer et al. (1983), Ates (2003), and Williams and McDermott (2004); *Mammillifera* sp. or *Mammillifera* sp., by Balss (1924, 1927), and Kaestner (1970); *Anemonia mammifera*, by Nicol (1967); and *Actinia equina*, by Williams and McDermott (2004). Thus, the true identity of the cnidarian living with *P. typica* still remains uncertain.

Despite the uniqueness of *Paguropsis* among the Diogenidae and even Paguroidea as a whole, the morphology of *P. typica* has not been studied in sufficient detail in light of the modern concept of paguroids. Furthermore, aside from the inclusion of this genus and species in phylogenetic studies related to the origin of lithodids and the phenomenon of carcinization (Richter and Scholtz 1994, McLaughlin and Lemaitre 1997), the evolutionary significance and position of *Paguropsis* among paguroids has remained unexplored. This knowledge deficiency can be attributed, in part, to the fact that the material of *P. typica* reported in the literature is scattered in a few museums, with the majority of specimens originally deposited and largely unavailable, in the Indian Museum, Kolkata. The opportunity to examine new material of *Paguropsis* came when fresh specimens assignable to this genus were found while studying the rich paguroid collections obtained during various collaborative French deep-water faunistic expeditions to the tropical western Pacific. The detailed examination of this new material, and comparison with the types and other previously unreported specimens identified under *P. typica* and deposited in various museums, revealed that *Paguropsis* is not monotypic or even monophyletic, and that in addition to *P. typica*, there are six more species, five of them new to science, all confounded under what was previously believed to be a single species. Altogether, 1042 specimens were examined. Here it is shown that Alcock’s (1899) name *P. andersoni* needs to be resurrected from the synonymy of *P. typica* as a valid species. Furthermore, a detailed comparative morphological analysis has shown that two of the five new species discovered, although closely related to *P. typica*, are morphologically so fundamentally different in characters of the mouthparts, cephalothoracic sternite, chela of pereopods 4, and position of pleopods that they require placement in a new genus, *Paguropsina* gen. n. Thus, the genus *Paguropsis* is rediagnosed, and the new genus diagnosed. Both *P. typica* and *P. andersoni* are redescribed and illustrated, and all new species fully described and illustrated. Information on coloration is presented for all seven species with newly obtained color photographs. A key to the species of *Paguropsis* and *Paguropsina* gen. n. is provided, along with a brief discussion of the biogeography and significance of the morphology of the two genera among the Paguroidea. To assist in future phylogenetic studies, genetic data for each of the seven species is reported for the mitochondrial COI barcode region and partial sequences of 12S and 16S rRNA (Table 1).

**Table 1.** List of species, collection site, museum catalog number of vouchers, and GenBank accession numbers for which mitochondrial COI barcode region and partial sequences of 12S and 16S rRNA were obtained in this report.

Species	Locality	Voucher deposition	COI GenBank no.	16S GenBank no.	12S GenBank no.
*Paguropsis typica*	Philippines	MNHN-IU-2014-9399	MG759674	MG759703	MG773703
	Philippines	MNHN-IU-2014-9427	–	MG759705	–
	Philippines	MNHN-IU-2014-9411	MG759677	MG759704	MG773704
*Paguropsis andersoni*	Indian Ocean	USNM 42719	–	MG950177	MG950176
	Madagascar	MNHN-IU-2014-9393	MG759673	MG759692	MG773702
*Paguropsis confusa*	Philippines	MNHN 50015	MG759683	MG759696	MG773707
	Locality uncertain	MNHN-IU-2014-9420	–	MG759695	MG773705
	Philippines	USNM 1441901	MG759682	MG759694	MG773706
*Paguropsis gigas*	South China Sea	NTOU A01445	MG759678	MG759697	MG773709
	South China Sea	NTOU A01446	MG759679	MG759698	MG773710
*Paguropsis lacinia*	Papua New Guinea	MNHN-IU-2013-2288	MG759665	MG759699	MG773711
	New Caledonia	MNHN-IU-2014-9360	MG759666	MG759700	MG773712
	Solomon Islands	MNHN-IU-2014-9367	MG759668	MG759701	MG773713
*Paguropsina pistillata*	New Caledonia	MNHN-IU-2014-9391	MG759672	MG759689	MG773695
	New Caledonia	MNHN-IU-2014-9401	MG759675	MG759690	MG773696
	New Caledonia	MNHN-IU-2014-9402	MG759676	MG759691	MG773699
*Paguropsina inermis*	Philippines	MNHN-IU-2014-9372	MG759669	MG759686	MG773700
	New Caledonia	USNM 1441892	MG759681	MG759684	MG773701
	New Caledonia	MNHN-IU-2014-9383	MG759670	MG759687	MG773698
	New Caledonia	MNHN-IU-2014-9362	MG759667	MG759685	MG773697
	South China Sea	NTOU A01447	MG759680	MG759688	MG773693

## Materials and methods

Specimens used for this study remain deposited in the following museums: The Natural History Museum, London, UK (formerly British Museum) (**BMNH**); Natural History Museum and Institute, Chiba, Japan (**CBM**); Lee Kong Chian Natural History Museum, Zoological Reference Collection of the Raffles Museum of Biodiversity Research, National University of Singapore (**LKCNHM ZRC**); Muséum national d’Histoire naturelle, Paris, France (**MNHN**); National Museum of the Philippines, Manila (**MNHN**); National Taiwan Ocean University, Keelung, Taiwan, ROC (**NTOU**); National Natuurhistorisch Museum, Naturalis Biodiversity Center, Leiden (formerly Rijksmuseum van Natuurlijke Histoire), the Netherlands (**RMNH**); Iziko South African Museum, Cape Town, South Africa (**SAMC**); National Museum of Natural History, Smithsonian Institution, Washington DC USA (**USNM**); and the Zoological Museum, University of Copenhagen, Denmark (**ZMUC**).

General morphological terminology follows that of McLaughlin (2003) and Tudge et al. (2012), except for the use of “pleon” instead of “abdomen” used according to Schram and Koenemann (2004). The term “chelipeds” applies to pereopod 1, and “ambulatory legs” to pereopods 2 and 3. Paired pleopods 1 and 2 in males, and paired pleopod 1 in females, are referred to as gonopods. Cephalothoracic somites and their sternites, are numbered I–XIII (five cephalic and eight thoracic), and thoracomeres are I–VIII (three maxillipeds and five pereopods). The measurements indicated for the specimens refer to shield length (SL) in millimeters (mm), measured to the nearest 0.01 from the midpoint of the rostral lobe to the midpoint of the posterior margin of the shield. Miscellaneous abbreviations used are:

**CC** otter trawl;

**CP** beam trawl;

**DW** Warén dredge;

**FB** fishing boat;

**INVMAR** Invertébrés Marins (for collections of MNHN);

**NO** navire océanographique;

**ovig** ovigerous female(s);

**sta** station;

**RV** research vessel;

**ROV** remotely operated vehicle;

**TRV** trawling vessel;

**USFC** United States Fish Commission.

The station data for the following French collaborative campaigns have been obtained from the MNHN website (https://expeditions.mnhn.fr/), published reports (Richer de Forges 1990), or supplied by MNHN staff (P Bouchet, L Corbari, P Martin-Lefèvre, or P Maestrati): BIOPAPUA; BORDAU 1, 2; BATHUS 3; CREVETTIERE; EBISCO; KARUBAR; LITHIST; LUMIWAN; MADEEP; MAINBAZA; MUSORSTOM 1–3, 5, 6; NANHAI; NORFOLK 1, 2; PANGLAO; SALOMON 1, 2; ZHONGSHA. Other expedition or research program abbreviations: DST/NRF ACEP, Department of Science and Technology/National Research Foundation and African Coelacanth Ecosystem Programme or “Spatial Solutions Project”. The original, official format (DDM or DDS) for reporting latitude and longitude for these expeditions has been retained in order to avoid confusion. Months are indicated using the first three letters.

Vouchers, tissue samples, and sequence data of specimens for DNA Barcoding or other genetic analysis were deposited in the MNHN or USNM. All PCR, sequencing, and analytics were carried out at the Laboratories of Analytical Biology at the National Museum of Natural History, Smithsonian Institution. Mitochondrial COI barcode region and partial sequences of 12S and 16S rRNA were obtained by standard protocols (see Evans and Paulay 2012). Unaligned sequences for at least one type specimen of each new species were accessioned to GenBank along with archival data for the respective voucher specimens listed in Table 1. Although this data is included as diagnostic identifiers for the species, and for future phylogenetic studies, a preliminary analysis was performed to generate a tree. Alignments for each locus were obtained using the L-INS-i alignment strategy in MAFFT version 7 (Katoh and Standley 2013). The aligned sequences were concatenated using Sequence Matrix (Vaidya et al. 2011). Phylogenetic analyses were performed on a concatenated dataset (12S + 16S + COI) using Maximum Likelihood (ML) with RAxML (Stamatakis 2006). ML options for RAxML included the GTRCAT model of nucleotide evolution (-m), rapid bootstrap analysis, and search for best-scoring ML tree (-f a), and 1000 bootstrap replicates.

## Systematic account

### Family Diogenidae Ortmann, 1892

#### Genus Paguropsis

Taxon classificationAnimaliaDecapodaDiogenidae

Henderson, 1888

Paguropsis Henderson, 1888: 98 (type species by monotypy: Paguropsis typica Henderson, 1888, gender feminine); Stebbing, 1893: 169; Alcock, 1905: 27; Gordan, 1956: 325; McLaughlin and Lemaitre, 1997: 112 (phylogeny); McLaughlin, 2003: 114 (key); McLaughlin et al., 2010: 23; McLaughlin, 2015: 153, fig. 6.3D.
Chlaenopagurus Alcock, 1899: 113 (type species by monotypy: Chlaenopagurus andersoni Alcock, 1899, gender masculine).

##### Diagnosis.

**Figure 1.**
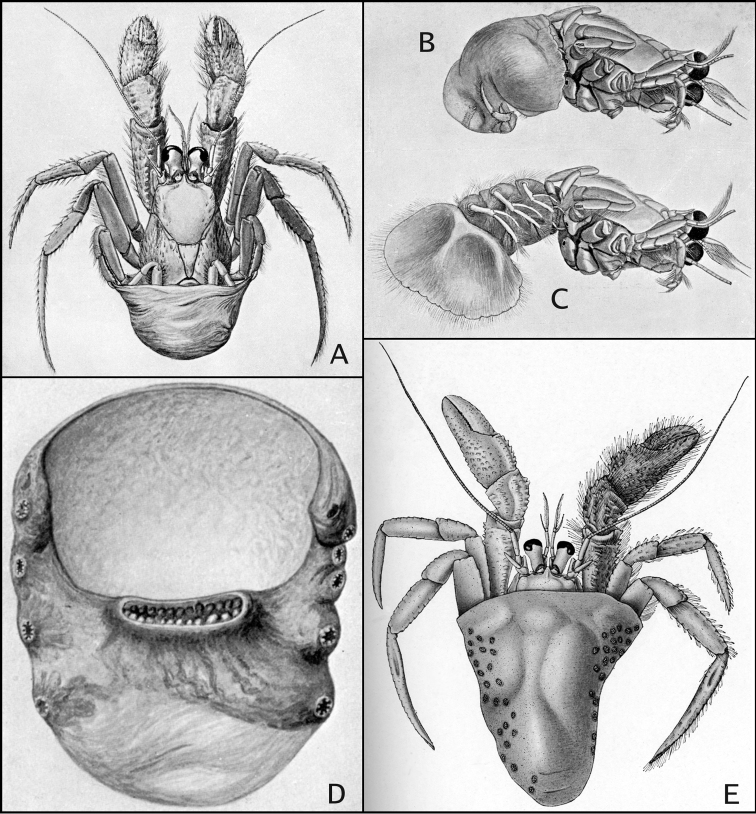
**A–C** *Paguropsis typica* Henderson, 1888, after Boas (1926: figs 1, 11A, B): **A** habitus **B, C** female right side with carcinoecium and pereopods 1–3 removed, with brood pouch (**B**) and with brood pouch folded posteriorly (**C**) showing pleopods **D** carcinoecium: *Epizoanthus paguropsidis* Ates, 2003 [nomen nudum], after Boas (1926: fig. 2) **E** *Paguropsis andersoni* (Alcock, 1899), after Alcock (1902: fig. 2), habitus.

**Figure 2.**
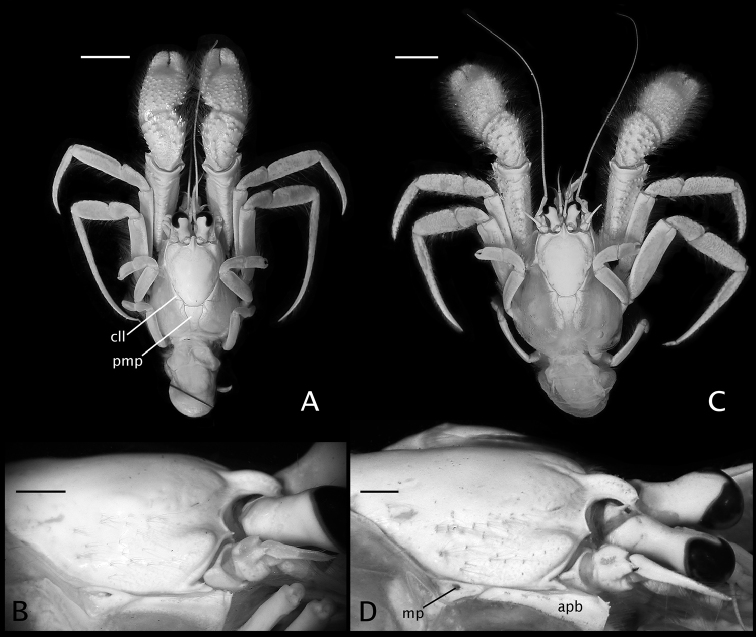
**A, B** *Paguropsis typica* Henderson, 1888 male, 14.6 mm, Philippines, USFC *Albatross*, sta 5409 (USNM 1107574) **C, D** *Paguropsis andersoni* (Alcock, 1899) lectotype male, 18.3 mm, Laccadive Sea, Indian Ocean, HM Indian Marine Survey Steamer *Investigator* (USNM 42719). **A, C** dorsal view **B, D** right side of shield and rostrum, branchiostegite, and anterodorsal portion of posterior carapace. Abbreviations: apb, anterodorsal plate of branchiostegite; mp, median plate; cll, carapace lateral lobe; pmp, posteromedian plate. Scale bars: 10 mm (**A, C**), 2 mm (**B, D**).

**Figure 3.**
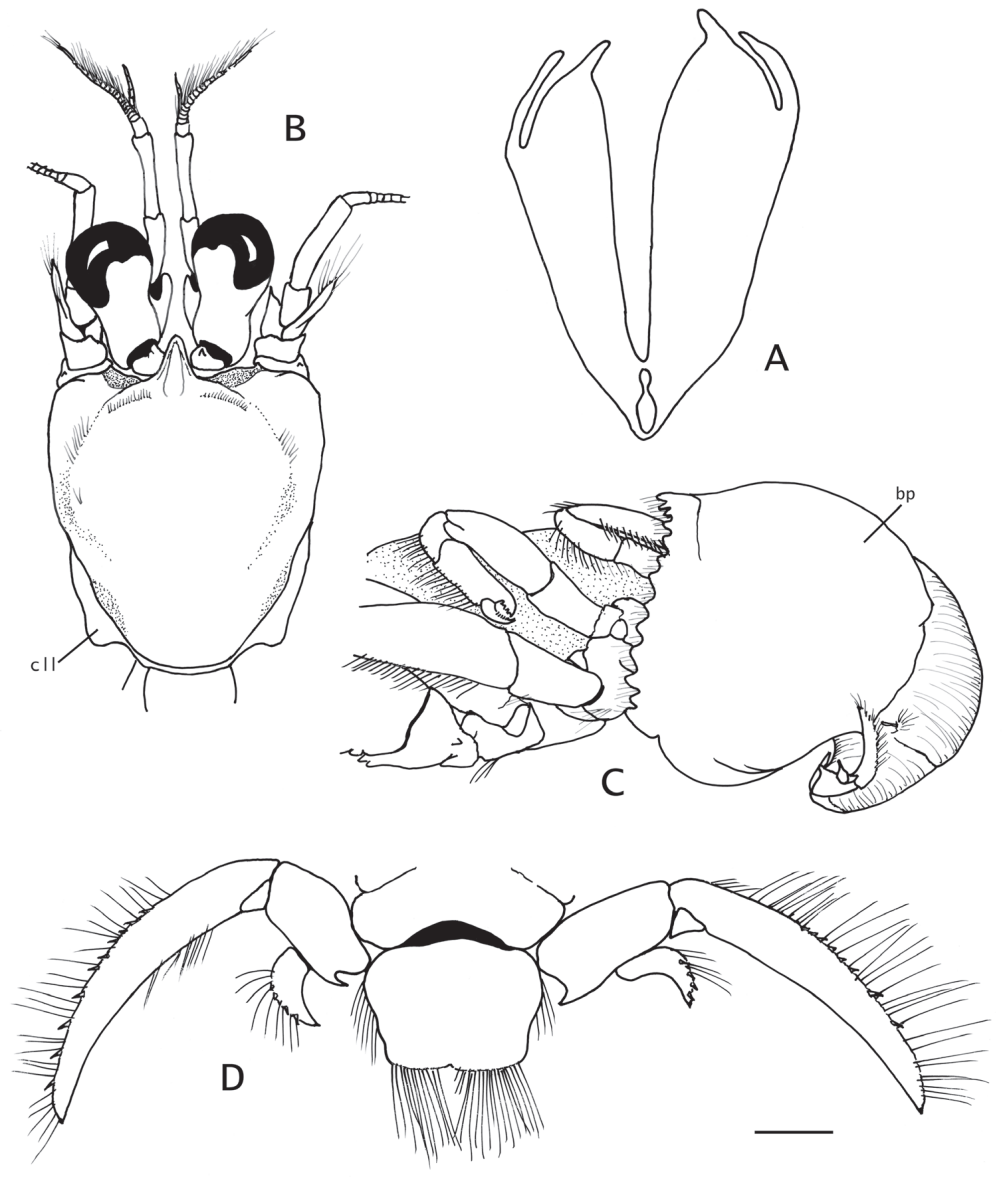
*Paguropsis typica* Henderson, 1888, Philippines, USFC *Albatross*, sta 5409: **A, B, D** male, 14.6 mm, **C** female, 7.1 mm (USNM 1107574). **A** gill lamella **B** shield and cephalic appendages, dorsal **C** left side of posterior thorax and pleon showing brood pouch (bp), lateral **D** uropods and telson, dorsal. Abbreviations: cll, carapace lateral lobe; bp, brood pouch. Scale bar: 0.5 mm (**A**), 4 mm (**B**), 2 mm (**C, D**).

Thirteen pairs of quadriserial gills [no pleurobranchs on thoracomere VIII (last)], gills consisting of series of twin lamellae each ending on distolateral and distomesial angles in filamentous or stub-like extensions (e.g., Fig. 3A). Shield well calcified, subtriangular or subrectangular; dorsal surface somewhat vaulted; lateral projections broadly triangular, each terminating in small spine. Rostrum prominent and projecting anteriorly, subtriangular, arched and dorsally ridged. Branchiostegite with dorsal margin (e.g., Fig. 2B, D) divided into two calcified plates: one anterodorsal plate poorly delimited ventrally, and one small, subtriangular median plate with distinct central pit. Posterior carapace (e.g., Fig. 2A, C) with well calcified posteromedian plate, and well calcified lateral lobe on each side adjacent to shield. Ocular peduncles short, approx. half as long as shield; corneas dilated (diameter typically half or slightly more than length of ocular peduncle, including cornea); ocular acicles relatively small, subtriangular, armed with small dorsodistal spine. Antennal peduncles distinctly exceeding distal margins of corneas; acicles long, reaching to level of corneas. Mouthparts: maxillule with well-developed and strongly recurved external lobe of endopod; maxilliped 1 with exopodal flagellum, endopodite medially bent at nearly right angle, with distinctly developed epipod; maxilliped 3 ischium with well-developed crista dentata, lacking accessory tooth, exopod slender, 4 or more times as long as broad. Epistome unarmed. Chelipeds symmetrical or nearly so, subequal in size, armed with moderately dense to dense setation and numerous well-spaced small spines or tubercles; coxae each with ventral surface having an uncalcified median longitudinal fissure starting on distal margin and incompletely covering length of ventral surface. Pereopods 2 and 3 long; dactyl of pereopods 3 distinctly longer than dactyl of pereopod 2. Sternite XI (between pereopods 3; e.g., Fig. 5B, D) narrow, separating coxae of pereopods 3 by distinctly less than half length of one coxa (typically 0.2 to 0.3 length of one coxa); anterior lobe flat or slightly concave, posterior lobes broad, arched and forming arrowhead shape with apex directed anteriorly. Pereopod 4 chelate, extending to subdorsal position to manipulate carcinoecium (e.g., Fig. 1A, 2A, C), lacking rasp-like surfaces; dactyl with cutting edge armed with row of small corneous spines; fixed finger with sharp spines on cutting edge arranged like bear claw; coxae (e.g., Fig. 5B, D) with anteroventral margin sharply delimited, keel-like. Sternite XII (between pereopods 4; e.g., Fig. 5B, D) broad, ridge-like, weakly divided medially, with fringe of setae. Pereopod 5 chelate, with weakly-developed propodal rasp. Pleon curling under but not dextrally or sinistrally twisted; pleonal somite 1 not fused to last thoracic somite, with partly calcified tergite and pleura. Male with well-developed paired gonopods 1 and 2, and reduced (uniramous or biramous) pleopod 3–5 on left or right side (occasionally lacking pleopod 5), or altogether lacking pleopods 3–5. Female with paired gonopores; with paired uniramous pleopods 1 modified as gonopods (Fig. 7D); left or right side of pleon with well-developed biramous unpaired pleopods 2–4 (ovigerous) and reduced biramous or uniramous unpaired pleopod 5 (not ovigerous, occasionally absent); brood pouch large (e.g., Figs 1C, 3C), covering pleopods 2–4 and entire egg mass. Uropods and telson symmetrical; exopods long, slender; endopod small, curved. Telson subquadrate or subrectangular, lacking or with obscure lateral indentations; posterior margin weakly divided into broadly rounded lobes.

##### Distribution.

Subtropical to tropical Indo-West Pacific. Depth: 30 to 1125 m.

##### Habitat and symbionts.

Several cnidarian names have been reported in the literature as symbionts of what has been presumed to be *P. typica*, including: *Epizoanthus paguropsidis* [e.g., Boas (1926), Schäfer et al. (1983, as *E. paguropsides*), Ates (2003), Williams and McDermott (2004)]; *Mammillifera* sp. [e.g., Alcock (1905), Balss (1924, 1927), Ross (1983)]; *Actinia equina* [e.g., Williams and McDermott (2004)]. There is considerable taxonomic confusion on these cnidarian names. The name *E. paguropsidis* is considered a nomen nudum apparently introduced by Boas (1926), and attributed to Ates (2003, as per WoRMS Editorial Board 2018). Schäfer et al.’s (1983) study identified the host of the cnidarian as *P. typica*, but that host is shown herein to actually apply to a species of Parapaguridae. *Mammillifera* is currently a subjective junior synonym of *Zoanthus*. Given the discovery of several species previously confounded under *P. typica* as well several new species, and the general unavailability of hermit crab materials that go along with reports of cnidarian symbionts, it is impossible to ascertain the identity of the cnidarian as well as to which species of *Paguropsis* or the new genus described herein. Those symbiont names apply to the Zoanthidea (often called Zoantharia), and those associated with hermit crabs that are typically assigned in the literature to colonial species of the genus *Epizoanthus*, (JD Reimer, pers. comm.; see Fig. 1D). However, the symbionts found with species of *Paguropsis* are actually indeterminate species of acontiate anemones which belong to the Actiniaria (DG Fautin, pers. comm.), and are herein reported as such.

##### Type species.

*Paguropsis typica* Henderson, 1888, by monotypy. Gender: feminine.

##### Species included.

In addition to the type species, *P. typica*, the genus includes: *P. andersoni* (Alcock, 1899), and three new species described herein.

##### Remarks.

Henderson (1888) considered *Paguropsis* and its only species at that time, *P. typica*, to be unique among hermit crabs based on the peculiar subdorsal position of pereopods 4 and 5 adapted to manipulate the symbiont zoanthid, the presence on the left or right side of the pleopods, and the straight pleon. Alcock (1905) considered his monotypic genus *Chlaenopagurus* Alcock, 1899 to be a junior synonym of *Paguropsis*, although he provided no explanation for that taxonomic action. Alcock (1905) suggested that *Paguropsis* was closely related to *Paguristes* Dana, 1851, but differed in the former having stout eyestalk, a non-coiled pleon, symmetrical tail fan (uropods and telson), chelate pereopod 4, and indifferent position (left or right side) of the pleopods. The taxonomy or morphology of *Paguropsis* has not been discussed or revised since that early time, although Boas (1926) did study in detail the morphology of *P. typica* relative to its symbiotic zoanthid.

During this study, several important characters previously overlooked or not sufficiently discussed have been added to the diagnosis of *Paguropsis*. Among these are the shapes of mouthparts (maxillule external lobe of endopod, and maxilliped 3 exopod); presence on posterior carapace of a well delimited calcified lateral lobe (e.g., Fig. 2A) fused to shield; on the branchiostegite (e.g., Fig. 2B, D), presence of a well calcified median plate with a central pit adjacent to the cervical groove, and an anterodorsal plate; presence on ventral surface of coxae of chelipeds of a longitudinal, uncalcified fissure (e.g., Fig. 5B, D); shape of thoracic sternites XI and XII (between pereopods 3 and 4 respectively; e.g., Fig. 5B, D); development of a full chela lacking rasp on pereopod 4, and distinct, bear-like claw armature of fixed finger; and sharply delimited anteroventral margin of coxae of pereopods 4 (e.g., Fig. 5B, D). Furthermore, coloration has been found to be unique for each of the species (Figs 8, 18A, B, 28A–D).

#### Paguropsis typica

Taxon classificationAnimaliaDecapodaDiogenidae

Henderson, 1888

Figs 1A–C , 2A, B , 3 , 4 , 5A, B , 6 , 7 , 8A, B , 14A , 28A , Table 1

Paguropsis typicus Henderson, 1888: 99, pl. 10, fig. 4 (type locality: HMS *Challenger*sta 204A or B, off Tablas Island, Philippines); Murray, 1895: 789.
Paguropsis typicus : Pzibram, 1905: 199; Boas, 1926: 1, figs 1, 7, 8–11; Rabaud, 1941: 263; Gordan, 1956: 325 (in part). (See “Remarks”).
Paguropsis typica : Estampador, 1937: 55; Balss, 1924: 775, figs 31, tbl. 2 (see “Remarks”); Balss, 1956: 1429 (in part); Nicol, 1967: 583; Kaestner, 1970: 299; Miyake, 1982: 98, pl. 33, fig. 6 (color photo); Schäfer et al., 1983: figs 12 (in part, see “Remarks”); Ross, 1983: 171; Baba et al., 1986: 192 (fig. 141, color photo), 193 (Japanese text), 299 (English text); Richter and Scholtz, 1994: 189 (phylogeny); Ates, 2003: 42, tbl. 1; Williams and McDermott, 2004: 16, tbl. 1; McLaughlin et al., 2010: 23; McLaughlin, 2015: 152, fig. 6.3D; Malay et al., 2018: 55.
*Paguropsis tyica* (misspelling): Schäfer et al., 1983, fig. 12 (in part, see “Remarks”)
Not Paguropsis typica: Schäfer et al., 1983: 229, figs 1, 2; Williams and McDermott, 2004: 12, 66, tbl. 1 (= glaucothoë stage of Parapaguridae, see “Remarks”)

##### Type material.

Lectotype (herein selected), male 6.0 mm, Tablas Island, Philippines, HMS *Challenger*, sta 204A to 204B, 12°43' to 12°46'N, 122°09' to 122°10'E, 182.9–210.3 m, 2 Nov 1874 (BMNH 1888.33). Paralectotype: 1 female 7.1 mm, same data as lectotype (BMNH 1888.33).

##### Other material.

*Japan*: Intensive Research of Unexploited Fisheries Resource on Continental Slopes, Japan Fisheries Resources Conservation Association, FB *Shin’ei-maru No. 53*, Kita-Koho Seamount, Kyushu-Palau Ridge, 26°46'09"N, 135°20'03"E, 360 m, 17 Nov 1978, trawl: 4 males 10.2–16.4 mm (CBM-ZC 4898); same data, 4 females 10.3–13.2 mm (CBM-ZC 4899).

*South China Sea*: NANHAI 2014, cruise OR5: sta DW 4105, 13°57.8902'N, 115°25.5073'E, 297–565 m, 3 Jan 2014: 1 male 3.6 mm (NTOU A01442). ZHONGSHA 2015, cruise ORI 1113: sta CP 4149, 16°06.54'N, 114°20.05'E, 165 m, 26 Jul 2015: 1 male 5.8 mm, 1 female 6.6 mm (NTOU A01443); sta CP 4150, 16°06.602'N, 114°21.45'E, 162 m, 26 Jul 2015: 1 male 6.0 mm (NTOU A01444). Hong Kong: Cruise 4/63, sta 66, Transect 56, [no locality, coordinates, depth, or date], coll. Fisheries Research Station: 1 female 5.8 mm (MNHN-IU-2014–9438).

*Philippines*: USFC *Albatross*, Philippines Expedition: Quezon, Luzon Island, Tayabas Bay, Lucena City, sta 5369, 13°48'00"N, 121°43'00"E, 193.8 m, 24 Feb 1909: 1 male 10.7 mm, 1 female 5.7 mm (USNM 1107610); Quezon, Luzon Island, Tayabas Bay, Lucena City, sta 5371, 13°49'40"N, 121°40'15"E, 151.8 m, 24 Feb 1909: 1 male 10.4 mm, 1 female 6.2 (molted, parasitized) (USNM 1107593); Quezon, Luzon Island, Tayabas Bay, Unisan, sta 5375, 13°42'15"N, 121°50'15"E, 195.7 m, 2 Mar 1909: 1 male 12.6 mm (USNM 1107608); Visayan Sea, Leyte Island, Villaba, near Capitancillo Island, sta 5403, 11°10'00"N, 124°17'15"E, 332.8 m, 16 Mar 1909: 1 male 11.0 mm (USNM 1107573); Camotes Sea, Cebu, Camotes Islands, NW of Pacijan Island, sta 5408, 10°40'15"N, 124°15'00"E, 290.8 m, 18 Mar 1909: 2 males 11.8, 12.8 mm, 1 female 11.7 mm, 1 ovig female 11.4 mm (USNM 1107590); Camotes Sea, Cebu, Camotes Islands, W of Pacijan Island, sta 5409, 345.6 m, 18 Mar 1909: 2 males 10.9, 14.6 mm, 1 female 8.5 mm (USNM 1107574); Cebu Island, Bohol Strait, SW of Lauis Point, sta 5411, 265.2 m, 23 Mar 1909: 2 males 8.6 mm, 13.3 mm, 2 females 11.2, 11.9 mm (USNM 1107594); Bohol Strait, Between Bohol and Cebu Islands, sta 5412, 10°09'00"N, 123°52'00"E, 296.3 m, 23 Mar 1909: 1 female 9.1 mm, 3 ovig females 10.9–11.8 mm (USNM 1107599); Cebu Island, Naga, Bohol Strait, sta 5417, 301.7 m, 25 Mar 1909: 4 males 7.9–13.2 mm, 2 females 7.1, 10.0 mm (USNM 1107584); Gulf of Albay, Albay, Luzon Island, E of S Luzon, sta 5454, 13°12'00"N, 123°50'30"E, 279.8 m, 7 Jun 1909: 1 female 7.8 mm (USNM 1107578); northern Mindanao, sta 5519, 8°47'00"N, 123°31'15"E, 332.8 m, 9 Aug 1909: 1 female 6.4 mm (USNM 1107588); Zamboanga del Norte, Mindanao Island, sta 5520, 10 Aug 1909, 186.5 m: 1 female 5.7 mm (USNM 1100423). MUSORSTOM 1, NO *Vauban*: N of Lubang, sta CC 12, 14°00'N, 120°17'E, 187–210 m, 20 Mar1976: 1 male 7.0 mm, 3 females 6.4–6.8 mm (USNM 1441983); N of Lubang, sta CP 18, 13°57'N, 120°17'E, 150–159 m, 21 Mar 1976: 1 ovig 7.5 mm (MNHN-IU-2014–9374); N of Lubang, sta CP 25, 14°02'N, 120°18'E, 191–200 m, 22 Mar 1976: 1 male 10.5 mm (MNHN-IU-2014–9373); N of Lubang, sta CP 27, 14°00'N, 120°16'E, 188–192 m, 22 Mar 1976: 22 males 4.6–4.8 mm, 25 females 3.9–9.0 mm (MNHN-IU-2014–9399); NW of Lubang, sta CP 54, 13°56'N, 119°58'E, 975–1125 m, 26 Mar 1976: 37 males 5.1–10.4 mm, 26 females 5.5–9.7 mm, 11 ovig females 6.7–8.3 mm (MNHN-IU-2014–9395); N of Lubang, sta CP 64, 14°00'N, 120°19'E, 194–195 m, 27 Mar 1976: 16 males 4.2–8.1 mm, 19 females 4.3–6.7 mm, 1 ovig female 7.0 mm (MNHN-IU-2014–9371). MUSORSTOM 2, NO *Coriolis*: N of Lubang, sta CP 01, 14°00'N, 120°18'E, 188–198 m, 20 Nov 1980: 5 males 5.8–11.0 mm, 2 females 6.0, 6.1 mm, 1 ovig female 9.6 mm (MNHN-IU-2014–9425); N of Lubang, sta CP 02, 14°00'N, 120°17'E, 184–186 m, 20 Nov 1980: 2 males 7.1, 9.4 mm, 1 female 6.7 mm, 2 ovig females 7.0, 7.8 mm (MNHN-IU-2014–9426), 1 ovig female 8.8 mm (MNHN-IU-2014–9431); between Luçon and Lubang, sta CP 06, 13°56'N, 120°22'E, 136–152 m, 20 Nov 1980: 1 female 6.7 mm (MNHN-IU-2014–9432); N of Lubang, sta CP 10, 14°01'N, 120°18'E, 188–195 m, 21 Nov 1980: 1 male 5.7 mm (MNHN-IU-2014–9429); N of Lubang, sta CP 11, 14°00'N, 120°19'E, 194–196 m, 21 Nov 1980: 2 males 6.1, 7.3 mm (MNHN-IU-2014–9427), 1 male 9.5 mm (MNHN-IU-2014–9428); N of Lubang, sta CP 18, 14°00'N, 120°17'E, 188–195 m, 22 Nov 1980: 1 female 6.4 mm (MNHN-IU-2014–9430); N of Lubang, sta CP 19, 14°01'N, 120°18'E, 189–192 m, 22 Nov 1980: 3 males 4.8–7.5 mm, 2 females 5.7, 5.8 mm, 1 ovig 6.0 mm (MNHN-IU-2014–9404); N Lubang, sta CP 53, 14°01'N, 120°17'E, 215–216 m, 27 Nov 1980: 1 male 7.2 mm (MNHN-IU-2014–9433); N of Lubang, sta CP 68, 14°00'N, 120°17'E, 195–199 m, 29 Nov 1980: 1 male 5.2 mm (MNHN-IU-2014–9434); N of Lubang, sta CP 71, 14°01'N, 120°19'E, 189–197 m, 30 Nov 1980: 1 female 6.0 mm (MNHN-IU-2014–9435); between Luçon and Lubang, sta CP 80, 13°45'N, 120°37'E, 178–205 m, 1 Dec 1980: 1 female 5.1 mm (MNHN-IU-2014–9436). MUSORSTOM 3, NO *Coriolis*: W of Luçon, sta CP 90, 14°00'N, 120°19'E, 195 m, 31 May 1985: 134 males 5.1–9.4 mm, 108 females 4.6–8.7 mm, 8 ovig females 6.4–7.8 mm (MNHN-IU-2014–9411), 16 males 5.1–9.7 mm, 11 females 4.9–7.5 mm, 9 ovig females 6.1–7.5 mm (MNHN-IU-2014–9413). PANGLAO 2004: sta T2, Bolod, Panglao Island, 9°32.4'N, 123°47.8'E, 152 m, coarse sand, 31 May 2004: not examined, color photos (#48, 54), Fig. 8A, B (ZRC or NTOU); Balicasag, [sta number unknown], May 2004: 1 male 12.1 mm (USNM 1441800). LUMIWAN 2008: NO *DA-BFAR*, sta CP 2870, 14°02'N, 120°17'E, 183–188m, 24 Mar 2008: 1 male, not examined (MNHN); [station unknown]: specimen not examined, color photograph, Fig. 28A.

*Papua New Guinea*: BIOPAPUA, NO *Alis*: Manus Island SE point, sta CP 3693, 02°10'S, 147°17'E, 300 m, 29 Sep 2010: 2 males 5.2, 6.7 mm (MNHN-IU-2014–2447), 10 males 4.3–6.7 mm, 6 females 4.2–5.3 mm, 4 ovig females 5.8–6.4 mm (MNHN-IU-2014–2653).

*Indonesia*: Danish Kai Islands Expedition: sta 44, 05°39'S, 132°23'E, 268 m, 30 Apr 1922: 1 female 12.2 mm (ZMUC-CRU–006723), 1 female 6.3 mm (ZMUC-CRU–006724); sta 49, 05°37'10"S, 132°24'E, 245 m, 3 May 1922: 1 female 8.7 mm (ZMUC-CRU 007031); sta 50, 05°34'S, 132°25'4"E, 233 m, 4 May 1922: 1 male 9.2 mm (ZMUC-CRU–006725). Dr. Th. Mortensen’s Expedition: Java, sta 2, 07°33'S, 114°36'E, 200 m, 3 April 1929: 1 male 8.6 mm (ZMUC-CRU–006987). KARUBAR, RV *Baruna Jaya 1*: Kai Islands, sta CP 35, 06°08'S, 132°45'E, 390–502 m, 27 Oct 1991: 1 female 6.9 mm (USNM 1441994); Tanimbar Islands, sta DW 49, 08°00'S, 132°59'E, 206–210 m, 29 Oct 1991: 1 male 3.6 mm (USNM 1441991).

*Fiji*: BORDAU 1, NO *Alis*: Lau Ridge, Lakeba, sta DW 1463, 18°10'S, 178°44'W, 300–400 m, 6 Mar 1999: 1 male 10.7 mm (MNHN-IU-2014–9364); Lau Lakeba Ridge, sta DW 1507, 18°09'S, 178°38'W, 255–290 m, 13 Mar 1999: 1 male 5.1 mm (MNHN-IU-2014–9365).

*New Caledonia*: MUSORSTOM 5, NO *Coriolis*: Coral Sea, Lord Howe Ridge, Capel Bank, sta CP 275, 24°46.60'S, 150°40.30'E, 285 m, 9 Oct 1986: 1 male 4.3 mm (USNM 1441993); Coral Sea, Lord Howe Ridge, Argosta Bank, sta DC 291, 23°07.70'S, 159°28.40'E, 300 m, 11 Oct 1986: 3 females 4.8–5.7 mm (USNM 1441992). MUSORSTOM 6, NO *Alis*: NW of Lifou, sta CP 419, 20°42'S, 167°04'E, 283 m, 16 Feb 1989: 2 males 5.7, 5.8 mm, 2 females 5.7, 6.0 mm (USNM 1441990). LIFOU 2000, NO *Alis*: Santal Bay, in front of Hacu Hutighé islet, sta DW 1647, 20°42'S, 167°08'E, 150–200 m, 6 Nov 2000: 1 female 11.4 mm (MNHN-IU-2014–9376). NORFOLK 1, NO *Alis*: Norfolk Ridge, Kaimon-Maru Bank, sta CP 1676, 24°44'S, 168°09'E, 227–232 m, 22 Jun 2001: 1 female 8.5 mm (MNHN-IU-2014–9356); Norfolk Ridge, Kaimon-Maru Bank, sta CP 1683, 24°44'S, 168°07'E, 248–272 m, 22 Jun 2001: 1 male 7.8 mm (MNHN-IU-2014–9361). NORFOLK 2, NO *Alis*: Kaimon-Maru Bank, sta DW 2093, 24°44'S, 168°09'E, 230 m, 29 Oct 2003: 1 female 10.3 mm (MNHN-IU-2014–9387); Kaimon-Maru Bank, sta CP 2095, 24°46'S, 168°10'E, 283–310 m, 29 Oct 2003: 1 female 12.5 mm (MNHN-IU-2014–9384). EBISCO, NO *Alis*: Capel Bank, sta CP 2492, 24°44'S, 159°41'E, 285 m, 6 Oct 2005: 1 male 4.3 mm (ex MNHN-IU–2014–9401, USNM 1441984); N of Bellona, sta DW 2578, 20°21'S 158°40'E, 440–505 m, 14 Oct 2005: 1 female 3.0 mm (USNM 1441995).

*Eastern Australia*: Queensland, *Nimbus* 1/68, sta 29, 26°30'S, 153°44'E, 184 m, 29 Jul 1968, coll. AJ Bruce: 1 male 7.0 mm (MNHN-IU-2014–9403, = MNHN-Pg 3670); sta 39, [same coordinates, depth as sta 29], 30 Jul 1968: 1 male 8.0 mm (MNHN-IU-2014–9410).

[*Locality uncertain*]: INVMAR: sta 15, [coordinates on label in error], 240 m, [no day] April 1929: 1 male 10.3 mm, 1 female 3.9 mm (MNHN-IU-2014–9416); sta 50, 233 m, [no other data]: 1 ovig female 8.1 mm (MNHN-IU-2014–9414); sta 52, [no other data]: 2 males 8.6, 12.7 mm (MNHN-IU-2014–9418, = MNHN-Pg 2313); sta 66, [no other data]: 2 females 5.4, 7.8 mm (MNHN-IU-2014–9422, = MNHN-Pg 2316); sta 125, 200 m, 4 Mar 1977 [no other data]: 1 female 10.9 mm (MNHN-IU-2014–9405); [no data]: 5 males 6.6–7.6 mm (MNHN-IU-2014 9415), 1 ovig female 7.1 mm (MNHN-IU–2014–9423, = MNHN-Pg 2315).

##### Redescription.

*Shield* (Figs 1A, 2A, 3B) subtriangular, ca. 1.2 times as long as broad; dorsal surface glabrous except for scattered setae and transverse fringe of short setae on sloping anterior margins of gastric region; anterior margin between rostrum and lateral projections concave; lateral projections broadly triangular, each terminating in small spine; posterior margin roundly truncate; lateroventral distal angle produced into strong spine adjacent to proximal margin of first segment of antennal segment, usually with 1 small sharp or blunt spine dorsally. Rostrum (Figs 2A, B, 3B) bluntly subtriangular, arched dorsally, strongly produced and extending slightly beyond distal margin of ocular acicles; with distinct rounded dorsal longitudinal ridge having few short setae laterally, ending smoothly or with 1 or 2 minute subterminal spines. Branchiostegites (Fig. 2B) unarmed except for 1 or 2 spines on dorsodistal angle of anterodorsal plate, and setose distal margin.

*Ocular peduncles* ca. 0.5 length of shield, constricted medially and noticeably broadened distally, glabrous or at most with row of short dorsomedian row of short setae; corneas strongly dilated, diameter 0.5–0.6 total peduncular length (including the cornea). Ocular acicles small, triangular, each armed with distal or dorsodistal spine often directed anterodorsally.

*Antennular peduncles* when fully extended overreaching distal margins of corneas by full length of ultimate peduncular segments. Ultimate and penultimate segments glabrous or at most with scattered short setae. Basal segment with ventromesial tuft of setae distally; lateral face with distal subrectangular lobe, small medial spine, and setose lobe proximally.

*Antennal peduncles* overreaching distal corneal margins by 0.3–0.4 lengths of ultimate segments. Fifth and fourth segments unarmed except for scattered setae. Third segment with spine at ventrodistal angle. Second segment with dorsolateral distal angle produced, terminating in small, usually bifid spine; mesial margin rounded, setose, dorsomesial distal angle with small, usually blunt spine. First segment unarmed. Antennal acicle reaching level of distal portion of optic calathus, slender, terminating in sharp spine, with long setae mostly distally; usually armed with row of 2 or 3 minute spines lateroproximally. Antennal flagellum long, reaching to distal end of cheliped fingers, articles with scattered short setae (< 1 flagellar article in length) and 1 or 2 longer setae (ca. 2 flagellar articles in length) every 12 articles or so.

**Figure 4.**
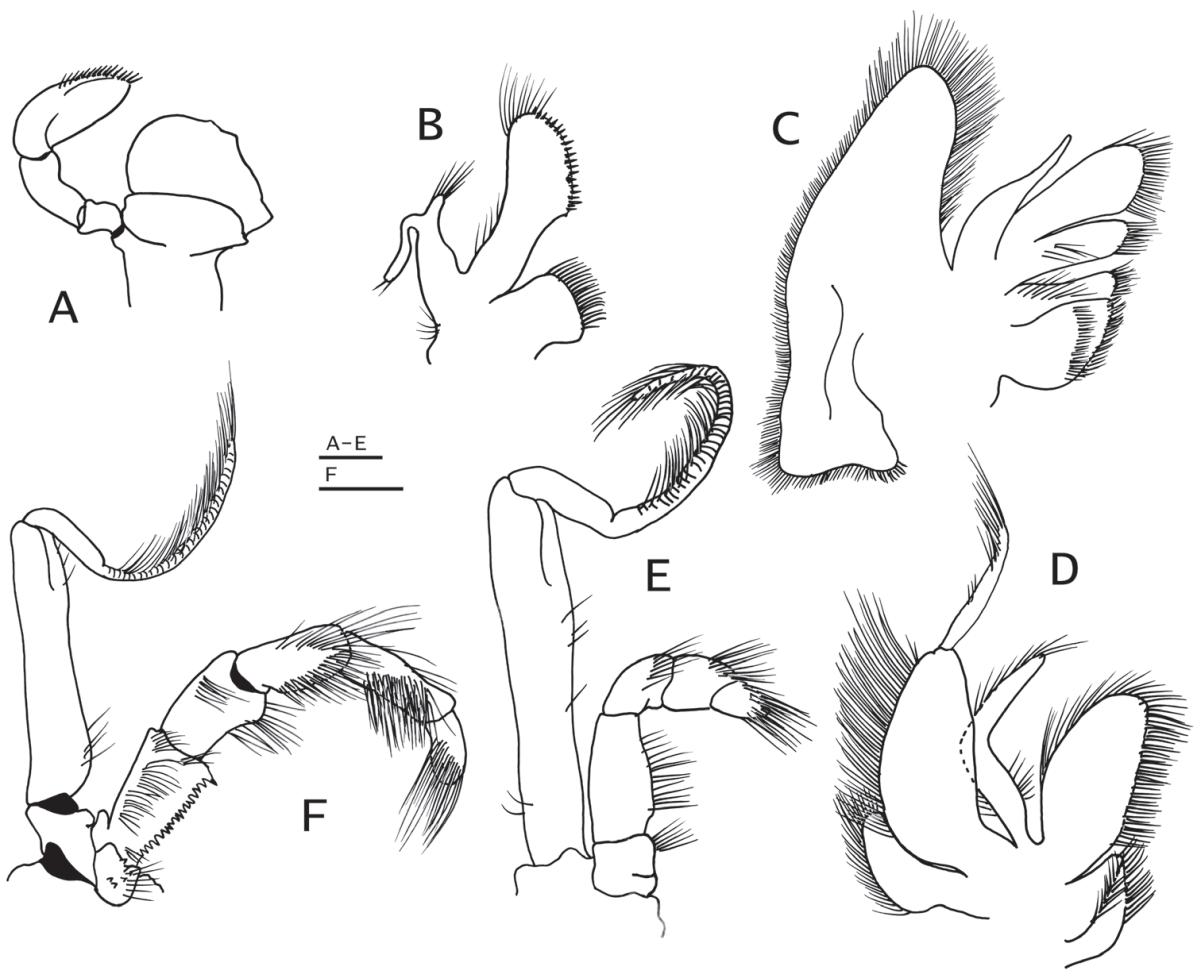
*Paguropsis typica* Henderson, 1888, female, 8.5 mm, Philippines, USFC *Albatross*, sta 5409 (USNM 1107574), left mouthparts, internal view: **A** mandible **B** maxillule **C** maxilla **D** maxilliped 1 **E** maxilliped 2 **F** maxilliped 3. Scale bars: 1 mm (**A–E**), 2 mm (**F**).

*Mandible* (Fig. 4A) with stout palp. Maxillule (Fig. 4B) with recurved external lobe of endopod nearly as long as endopod. Maxilla (Fig. 4C) with endopod not exceeding distal end of scaphognathite. Maxilliped 1 (Fig. 4D) with endopodite bent medially nearly at right angle, reaching distal end of exopod; with oval-shaped epipod. Maxilliped 2 (Fig. 4E) without distinguishing characters. Maxilliped 3 (Fig. 4F) exopod 4.5 times as long as broad; ischium having crista dentata armed with 15–17 small subequal (except for larger distal and proximal) corneous-tipped teeth; merus with 3–5 small spines on ventral margin, and usually two small spines on ventromesial distal angle; basis with row of small spines on mesial margin; coxa with ventromesial angle strongly produced ventrally, with 2–4 small spines and fringe of setae; sternite VIII narrow, with setose lobe on each side of midline.

**Figure 5.**
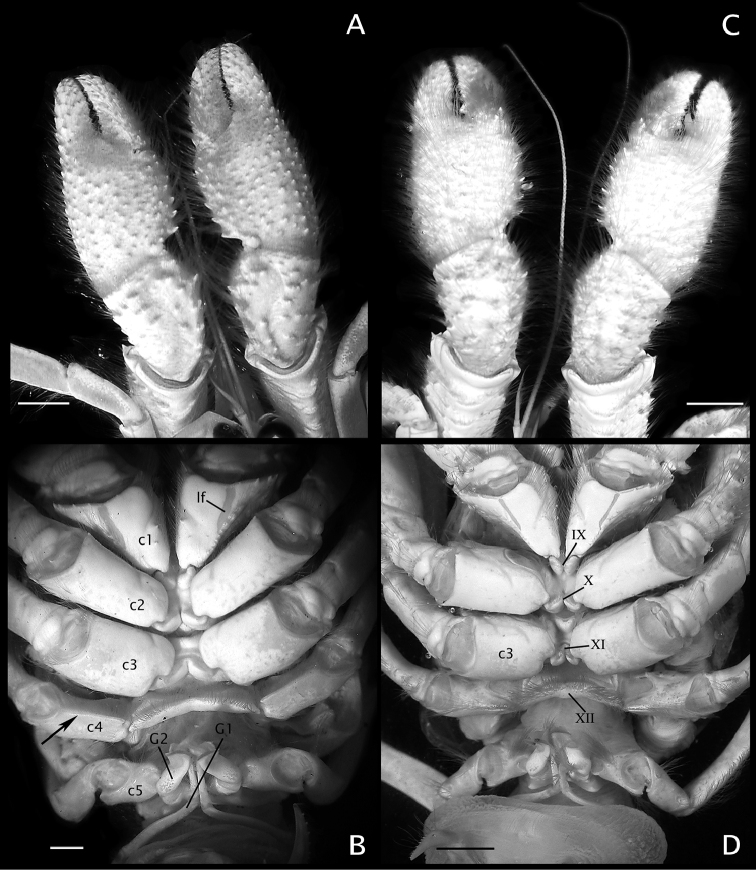
**A, B** *Paguropsis typica* Henderson, 1888 male, 14.6 mm, Philippines, USFC *Albatross*, sta 5409 (USNM 1107574); **C, D** *Paguropsis andersoni* (Alcock, 1899) lectotype male, 18.3 mm, Laccadive Sea, Indian Ocean, HM Indian Marine Survey Steamer *Investigator* (USNM 42719). **A, C** chelipeds, dorsal **B, D** coxae of pereopods 1–5, sternites IX–XIII, and anterior portion of pleon with gonopods 1 and 2 (arrow in B indicates keel-like anteroventral margin of coxa). Abbreviations: roman numerals refer to sternites; C1–5, coxae of pereopods 1–5; G1, G2, gonopods 1 and 2; lf, longitudinal fissure. Scale bars: 5 mm (**A, C**), 2 mm (**B**), 4 mm (**D**).

*Chelipeds* (Figs 1A, 2A, 5A) subequal, similar in armature and setation; dorsal surfaces of chelae and carpi covered with moderately dense tufts or short rows of bristle-like setae not hiding ornamentation beneath; ventral surfaces of palms smooth except for two submedian longitudinal rows of well-spaced low tubercles each with tuft of long bristle-like setae. Fingers with narrow hiatus proximally, forming spoon-like shape in ventral view when closed; each finger terminating in small curved corneous claw and subdistal blunt calcareous tooth ventral to claw, both claws and teeth interlocking when fingers closed; cutting edge of dactyl with row of small, fused corneous teeth on distal one-third, and row of unequal calcareous teeth on proximal two-thirds; cutting edge of fixed finger with row of blunt calcareous teeth decreasing in size distally. Dactyl ca. 1.2 times as long as palm; dorsal surface somewhat convex, armed with small spines proximally, dorsomesial margin rounded; ventral face with well-spaced tufts of long bristle-like setae, lacking spines. Fixed finger with dorsal, lateral, and ventral surfaces similar to dactyl in armature. Palm slightly shorter than carpus; dorsal surface covered with moderately dense, well-spaced small spines, accompanied by tufts of setae, arranged mostly in longitudinal rows (some extending to bases of fingers), and dense patch of plumose setae medially near base of fingers; dorsomesial margin with double row of well-spaced spines and tufts of long setae; dorsolateral margin rounded, not delimited, with irregular rows of small tubercles or spines, each accompanied by long setae. Carpus ca. 0.5 length of merus; dorsal surface with scattered simple spines or short transverse rows of 2 or 3 small spines accompanied by tufts of setae; dorsomesial margin with row of spines accompanied by tufts of setae, and dorsodistal spine; dorsolateral margin rounded; ventral surface smooth, with fringe of long setae on ventrodistal margin. Merus nearly as long as chela, subtriangular in cross-section; dorsal margin with row of protuberances each bearing transverse row of setae, ventromesial and ventrolateral margins each with row of spines with tufts of long setae; lateral and mesial surfaces with tufts of long and short setae mostly on ventral half. Ischium with row of small spines on ventrolateral margin. Basis with ventromesial row of long setae. Coxa with well-marked longitudinal fissure (Fig. 5B) on ventral surface.

**Figure 6.**
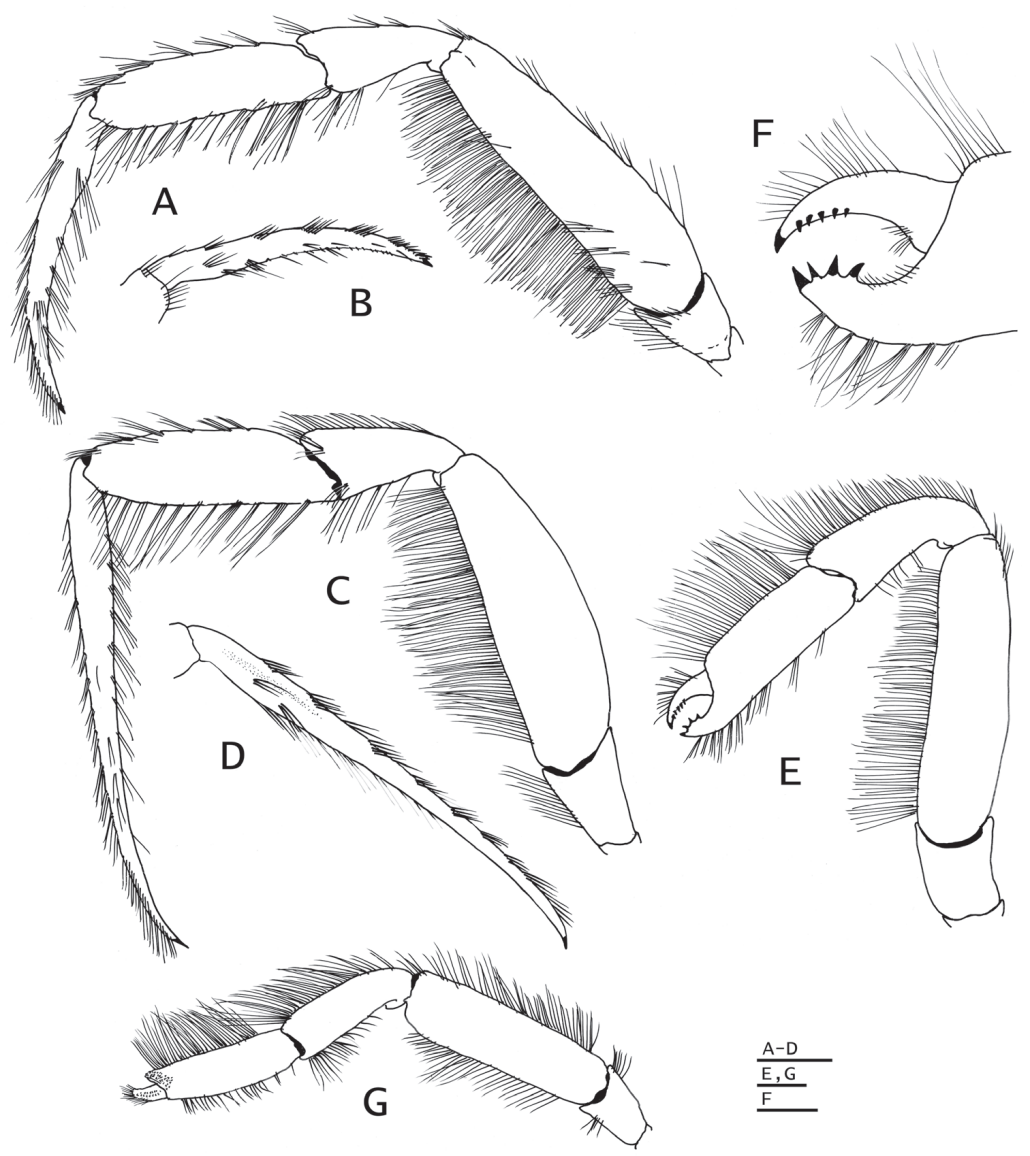
*Paguropsis typica* Henderson, 1888, male, 14.6 mm, Philippines, USFC *Albatross*, sta 5409 (USNM 1107574). **A** left pereopod 2, lateral **B** dactyl of same, mesial **C** left pereopod 3, lateral **D** dactyl of same, mesial **E** left pereopod 4, lateral **F** dactyl and fixed finger of same, lateral **G** left pereopod 5, lateral. Scale bars: 5 mm (**A–D**), 2 mm (**E, G**), 1 mm (**F**).

*Pereopods 2 and 3* (Figs 1A, 2A, 6A–D, 8A) similar in armature and setation, distinctly dissimilar in length, with pereopod 2 shorter than pereopod 3. Dactyls 1.5 (pereopod 2) or 2.5 (pereopod 3) times as long as propodi, each terminating in sharp corneous claw, lateral and mesial surfaces flat or very weakly convex; dactyl of pereopod 2 broadly curved on distal half; dactyl of pereopod 3 slender, nearly straight except for broadly curved distally near claw, from 1.6–1.8 times as long as dactyl of pereopod 2; dorsal margins with tufts of long setae, ventral margins with row of usually 14 short, minutely obscure corneous spinules (pereopod 2) or lacking spines or spinules (pereopod 3). Propodi 1.2 length of carpi; surfaces unarmed except for tufts of long setae on dorsal and ventral margins. Carpi unarmed except for tufts of setae dorsally and ventrodistally. Meri with fringe of long setae on ventral margins; ventral margin of pereopod 2 with row of few small, well-spaced blunt spines hidden by setae. Ischia unarmed except for scattered setae or tufts of setae (pereopod 2) or with ventral row of small spines (pereopod 3). Coxae of pereopods 3 (Fig. 5B) separated by 0.3 ventral length of 1 coxa. Sternite XI (between pereopods 3; Fig. 5B) having anterior lobe slightly concave and short setae on distal margin; posterior lobes each with transverse fringe of setae.

*Pereopod 4* (Fig. 6E, F) with chela 1.2 times as long as carpus and 3 times as long as high, palm 2.3 times as long as high. Dactyl and fixed finger widely gaping, each terminating in sharp, inwardly curved corneous claw crossing at tips when closed. Dactyl strongly curved inward, dorsal margin with row of short, sparse setae; cutting edge with ventrolateral row of 5–7 small corneous-tipped spines (in addition to corneous claw). Fixed finger curving inward, cutting edge of fixed finger with 3–5 corneous-tipped spines (in addition to corneous claw) arranged like bear claw; lateral face usually with one or two minute scale-like corneous spines near base of finger. Palm straight, dorsal margin with dense fringe of long setae often interspersed with fringe of shorter setae (occasionally slightly thickening distally), and few tufts of setae on ventral margin continued on fixed finger. Carpus unarmed except for fringe of long setae dorsally and scarce, moderately long setae ventrally. Merus long, 0.6 times as long as meri of pereopods 2 and 3. Sternite XII (between pereopods 4; Fig. 5B) broad, with fringe of long dense setae.

*Pereopod 5* (Fig. 6G) with chela 0.7 times as long as merus, with long, brush-like setae on dorsomesial and ventromesial faces; merus and carpus each with dorsal and ventral row of long setae. Dactyl with rasp on ventral face. Propodal rasp consisting of minute, ovate scales, occupying 0.1 length of propodus. Ischium with setae dorsally and ventrally. Coxa with fringe of long setae on ventrodistal margin.

**Figure 7.**
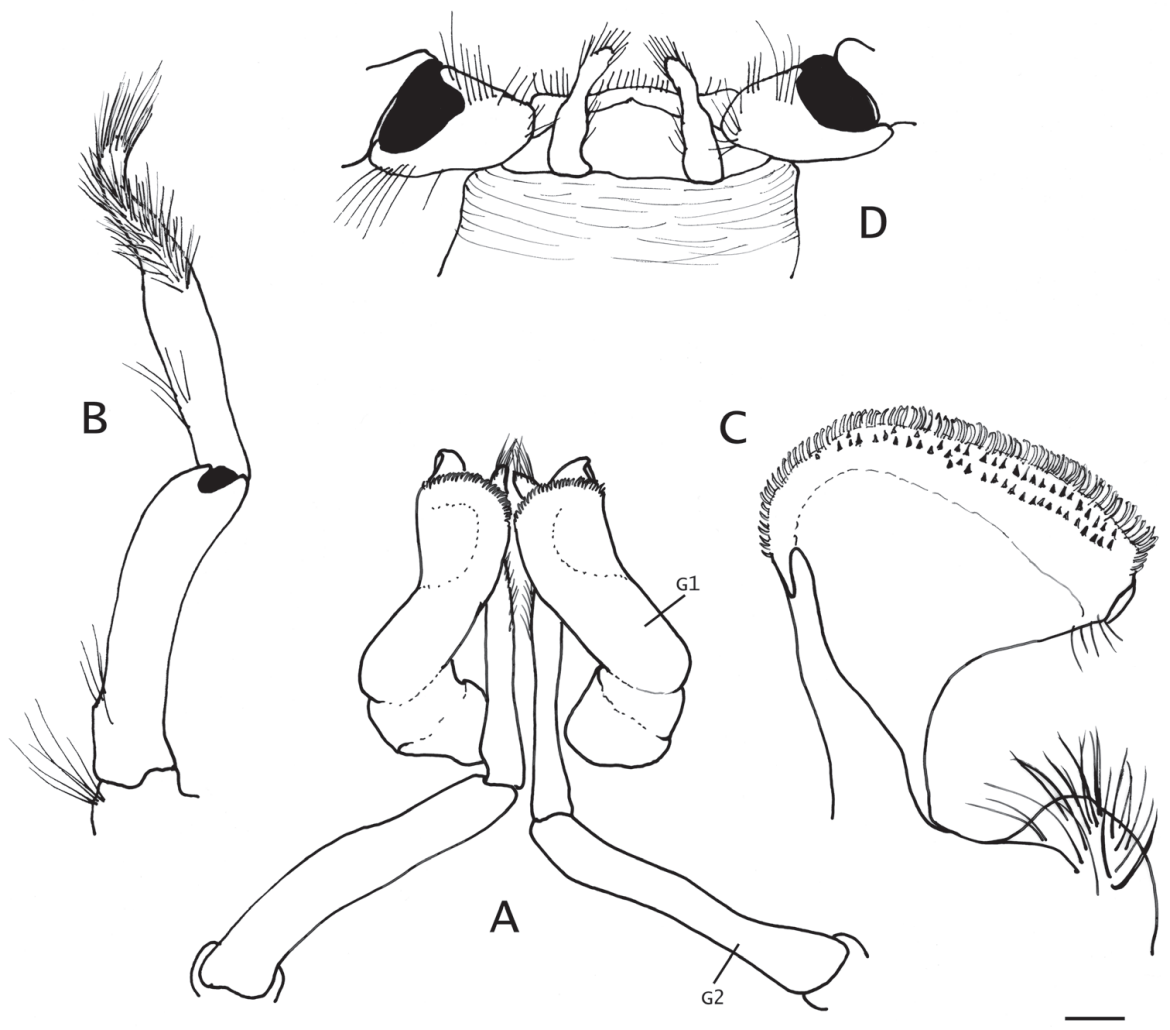
*Paguropsis typica* Henderson, 1888, Philippines, USFC *Albatross*, sta 5409 (USNM 1107574): **A–C** male, 14.6 mm, **D** female, 7.1 mm. **A** gonopods 1 and 2 *in situ*, ventroposterior **B** left gonopod 2, lateral, **C** distal portion of left gonopod 1, anterior **D** female gonopods 1 *in situ*, sternite XIII and coxae of pereopods 5. Abbreviations: G1, G2, gonopods 1 and 2. Scale bar: 1 mm (**A, B**), 0.25 mm (**C**), 0.5 mm (**D**).

*Male* gonopod 1 (Fig. 7A, C) with inferior lamella armed on distal margin with posterior row of slender, semitransparent hook-like spines, and 2 anterior rows of short straight or slightly curved corneous spines. Gonopod 2 (Fig. 7A, B) with distal segment strongly twisted distally, densely setose. Usually with unpaired, reduced pleopods 3–5 on left side or less frequently on right side, as follows: biramous or uniramous pleopod 3 and 4, and lacking or rarely with uniramous pleopod 5 (see “Variations”).

*Female* with unpaired pleopods 2–5 usually on left side or less frequently on right side, as follows: pleopods 2–4 biramous, well developed, and reduced biramous or uniramous vestigial pleopod 5. Brood pouch (Fig. 3C) large, subquadrate, distal margin scalloped and fringed with setae (see “Variations”).

*Uropodal exopods* (Fig. 3D) slender, broadly curved, terminating in strong spine, anterior margin with fringe of long setae and row of well-spaced corneous-tipped spines; endopods short, strongly curved, anterior margin with long setae and row of corneous-tipped spines; protopods with strong, curved proximal spine.

*Telson* (Fig. 3D) subrectangular, wider than long; posterior lobes separated by shallow median cleft, terminal margins unarmed except for fringe of long setae.

##### Genetic data.

See Table 1.

##### Color

**Figure 8.**
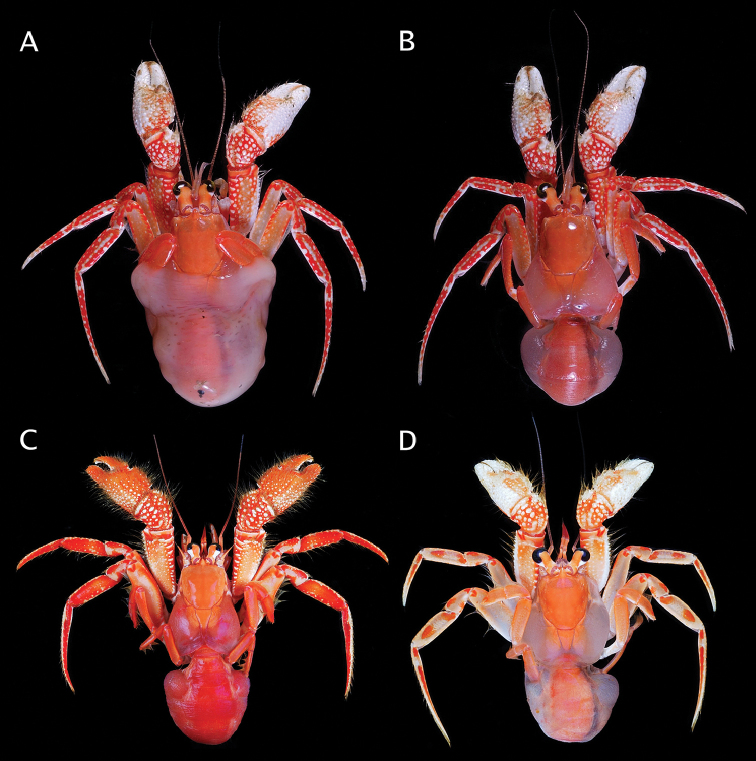
**A, B** *Paguropsis typica* Henderson, 1888, Philippines, PANGLAO 2004, sta T2 [#48] (**A**) and T2 [#54] (B) (photographs: T-Y Chan): **A** habitus, with carcinoecium **B** habitus, with carcinoecium removed **C** *Paguropsis andersoni* (Alcock, 1899), habitus, MAINBAZA sta CC 3159 (#29) (MNHN) (photograph: T-Y Chan) **D** *Paguropsis confusa* sp. n., MAINBAZA, sta CP 3134 (#23) (MNHN) (photograph: T-Y Chan).

(Figs 8A, B, 28A). Shield evenly orange. Ocular peduncles orange except for white along proximal and proximomesial margins of black corneas, orange tone darker medially; ocular acicles light orange fading to weak orange distally. Antennules light orange, flagella of similar color except for transparent bluish color distally. Antennal peduncles light orange, and similar but lighter toned and somewhat transparent flagella. Chelipeds with dactyl and fixed finger white; palms mostly white except for light orange distally and reddish proximally with white spines or tubercles; carpi red with white spines or tubercles; meri red except for orange on lateral and mesial surfaces, and white spines or tubercles. Pereopods 2 and 3 red with white spots on lateral faces of dactyls, carpi and propodi; dactyls each with uneven or interrupted white band proximally; meri with orange lateral and mesial faces; ischium with orange.

##### Distribution.

Western Pacific: from Japan, off Daito Islands, Ryukyu Islands (Miyake 1982), and Kyushu-Palau Ridge (Baba et al. 1986, this study); South China Sea; Philippines; New Guinea; Indonesia (Java and Arafura Seas); Fiji Islands; New Caledonia; and eastern Australia. Depth: 136 to 1125 m.

##### Habitat and symbiont.

Found with indeterminate species of acontiate anemone (see “Remarks” under genus).

##### Variations.

Aside from the meristics accounted for in the above redescription, this species is relatively constant in morphology. The only remarkable variation is in the position of unpaired pleopods 2–5 in females, and presence and degree of development of pleopods 3–5 in males. Females of *Paguropsis typica* have unpaired pleopods 2–5 on either side, with pleopods 2–4 well developed, biramous and ovigerous, whereas pleopod 5 is considerably reduced or absent. Males have unpaired pleopods 3–5 on either side, all considerably reduced, uni- or biramous, although pleopod 5 or occasionally all pleopods 3–5, can be absent. McLaughlin and Lemaitre (1997: 111) erroneously stated that males of *Paguropsis* lacked unpaired pleopods. Based on the specimens examined with complete pleons (n = 45), we observed that in females, 65.5% had pleopods 2–5 on the left side, 24.2% on the right side (often pleopod 5 is absent), and 10.3% did not have any unpaired pleopods on either side. In males, 75.1% had one or more of pleopods 3–5 on the left side, 18.7% on the right side, and 6.2% did not have any unpaired pleopods on either side.

##### Affinities.

Except for the drastic difference in coloration, *Paguropsis typica* and *P. confusa* sp. n. are remarkably similar in morphology, and can thus be easily confused unless fresh specimens that still retain their color patterns are available (Figs 8A, B, D, 28A, C, D). In the absence of color information, the two can be separated with difficulty using only subtle characters, such as the degree of setation and strength of armature of chelipeds and pereopods 2 and 3 (less dense setation and spination in *P. typica* than in *P. confusa* sp. n.), and minor differences on the lateroproximal surface of the dactyl of pereopod 3 (convex in *P. typica* vs. slightly concave in *P. confusa* sp. n.).

General similarities exist between *Paguropsis typica* and *P. andersoni*, in particular the cephalic appendages (i.e., dilation of corneas, development of antennular and antennal peduncles), and shape of shield (i.e., posterior half of shield narrowly subtriangular). However, the two species can immediately be differentiated by the shape of the dactyls of pereopods 2 and 3 (Figs 1A, E, 2A, C, 6A–D, 8A–C, 10A–D), which are distinctly more slender and narrower in *P. typica* than in *P. andersoni*; and more clearly, the lateral face of the dactyl of pereopod 3 is evenly flat or convex throughout in *P. typica*, whereas the surface is distinctly concave (and often weakly calcified) on the proximal one-third in *P. andersoni*. Both species have numerous tufts of long setae on chelipeds and ambulatory legs, but in *P. typica* these are not as dense or as stiff and bristle-like as in *P. andersoni*. The antennal acicles are shorter, reaching approx. to the level of distal portion of optic calathus, and the chela of each pereopod 4 is more elongate (1.2 times as long as carpus), in *P. typica*; whereas the acicles slightly exceed the distal margins of corneas, and the palm of each chelate pereopod 4 is shorter (0.6 times as long as carpus), in *P. andersoni*. Color differences also exist between these two species, and these are discussed under “Remarks” for *P. andersoni*.

##### Remarks.

Alcock (1905) corrected the spelling of Henderson’s (1888) species for gender agreement, from *typicus* to *typica*, although as shown here, his specimens actually represent *Paguropsis andersoni*. Nevertheless, various carcinologists (Pzibram 1905, Boas 1926, Rabaud 1941, Thompson 1943, Kamalaveni 1950, Gordan 1956) continued to use the original spelling *P. typicus*.

Since Henderson’s (1888) description of *Paguropsis typica* a good number of biologists have used Henderson’s taxon name in faunal inventories or checklists (e.g., Estampador 1937, Miyake 1982, Baba et al. 1986, McLaughlin et al. 2010, Malay et al. 2018), as example of symbiosis (Balss 1924, 1956, Ross 1983, Ates 2003, Williams and McDermott 2004), in textbooks on biology or anatomy of invertebrates (Nicol 1967, Kaestner 1970), in phylogenetic analyses (Richter and Scholtz 1994), and reviews of hermit crab housing (McLaughlin 2015). Of these, records from the Indian Ocean are referred to *P. andersoni* (see “Remarks” under *P. andersoni*). Gordan (1956), in her bibliography of pagurids, did include under *Paguropsis typica* (using the spelling *P. typicus*), references to both Henderson’s (1888) original description and Alcock’s (1899) *Chlaenopagurus andersoni* (= *Paguropsis andersoni*); thus, her taxon concept included both *P. typica* and the herein resurrected *P. andersoni*. The studies by Balss (1924, 1956) did include figures or information that clearly are referable to *P. typica* or *P. andersoni*, and thus are herein listed accordingly in the synonymy for each of these two taxa as “in part”. Miyake (1982) and Baba et al. (1986) reported on new specimens from Japanese waters. The specimen from off Daito Islands, shown in Miyake’s (1982) book, has an abnormally small left cheliped perhaps in the process of regeneration, but agrees well with *P. typica* in the general coloration, confirming its identity. The specimens from the Kyushu-Palau Ridge examined in this study (CBM-ZC 4898, 4899) originated from the same source as the material studied by Baba et al. (1986).

Schäfer et al. (1983) studied the morphology of an aberrant actinian which they found attached to the underside of the thorax of planktonic glaucothoe larvae they identified as *Paguropsis typica*. However, in a worldwide review of hermit crab biocoenoses Williams and McDermott (2004, based on pers. comm. from PA McLaughlin), questioned the identity of the hermit crab host name used by Schäfer et al. (1983). We have examined the glaucothoe larval specimens used by Schäfer et al. deposited in ZMUC, and found the larvae to actually belong to one or more indeterminate species of the family Parapaguridae. These larvae are of the type similar to “*Glaucothoe peronii*”, which were shown to represent parapagurids by de Saint Laurent-Dechancé (1964). Glaucothoe stages of several species of parapagurids have been described by Lemaitre and McLaughlin (1992) and Lemaitre (1997). Our assignment of Schäfer et al.’s (1983) glaucothoe to parapagurids is made based on the following characters present in those larvae: 1) maxilliped 1 lacking flagellum (a major parapagurid character); 2) pereopod 4 semi-chelate and with a propodal rasp (it is uniquely chelate and lacking a propodal rasp in *P. typica*); 3) pleonal pleura terminating ventrally in anteriorly directed hook-like process; 4) telson anterior half broad and rounded laterally, and narrow posterior half with long setae on terminal margin. Schäfer et al. (1983: fig. 12), however, based on the literature presented a map showing the known distribution of adult specimens of *P. typica* (misspelled therein also “*P. tyica*”), which includes historical records of both *P. typica* and the herein resurrected *P. andersoni*.

#### Paguropsis andersoni

Taxon classificationAnimaliaDecapodaDiogenidae

(Alcock, 1899), resurrected

Figs 1E , 2C, D , 5C, D , 8C , 9 , 10 , 14B , 28B , Table 1

Chlaenopagurus Andersoni Alcock, 1899: 115, pl. 1 (type locality: Indian Marine Survey *Investigator*, off Comorin).
Chlaenopagurus andersoni Alcock & McArdle, 1901: pl. 53, figs 1, 1a, 2, pl. 54, figs 1, 1a; Alcock, 1901: 229; Alcock, 1902: 67, fig. 2.
Paguropsis typica : Alcock, 1905: 28, pl. 2; Balss, 1924: 775, figs 30, 32 (see “Remarks” under P. typica); Balss, 1927: 963, fig. 1059; Thompson, 1943: 414 (see “Remarks”); Balss, 1956: 1429 (in part); Barnard, 1962: 240; Russell, 1962: 19, fig. 12; Sarojini and Nagabhushanam, 1972: 250, fig. I, fig. A, B, C; Kensley, 1981: 33 (list); Thomas, 1989: 59; Schäfer et al., 1983: figs 12 (in part, see “Remarks” under P. typica); Emmerson, 2016: 449 (list).
Paguropsis typicus : Thompson, 1943: 413 (see “Remarks”); Kamalaveni, 1950: 77, fig. 1 (see “Remarks”); Gordan, 1956: 325 (in part, see “Remarks” under P. typica).
*P. tyica* (misspelling): Schäfer et al., 1983, fig. 12 (in part, see “Remarks”)

##### Type material.

Lectotype herein selected: off Cape Comorin (Kanyakumari), Laccadive Sea, Indian Ocean, HM Indian Marine Survey Steamer *Investigator*, [probably sta 258, see “Remarks”], 23 Apr 1899, 08°23'N, 76°28'E, 186.5 m (102 fm): male 18.3 mm (USNM 42719, ex Indian Museum reg. no. 3173–5). Paralectotypes, [same sta data as lectotype]: 2 males 11.7, 17.6 mm (USNM 1441996, ex Indian Museum reg. no. 3173–5); 2 males 7.8, 8.2 mm (ZMUC-CRU–006727); 1 ovig female 8.1 mm (BMNH 1899.11.30.3).

##### Other material.

*Philippines*: NW coast of Panglao Island, 146.3–548.6 m, [no day] Jan-Mar 2011, coll. J Arbasto: 4 males 14.2–16.3 mm (LKCNHM ZRC 2011.0067). PANGLAO 2004: Balicasag Island, sta PN 1, 09°31'N, 123°41'E, 50–500 m, Apr-Jul 2004, from local fisherman: 1 male 16.6 mm (LKCNHM ZRC 2018.0171).

*Indonesia*: KARUBAR, RV *Baruna Jaya 1*: off Tanimbar Island (Arafura Sea), sta CP 46, 08°01'S, 132°51'E, 271–273 m, 29 Oct 1991: 7 males 9.3–16.1 mm (USNM 1442005).

*Indian Ocean*: southwestern India: off Neendakara, Munambam, Kerala State, 30 m, 2006, commercial trawler, coll. A Biju Kumar: 4 males 14.1–18.2 mm (CBM-ZC 10006). Kenya: off Mombasa, RV *Ujizi*, 03°09'S, 40°29'E, [no depth], 24 Mar 1980, coll. WJ Scheffers: 1 male 19.9 mm (RMNH.CRUS.D.34951). Seychelles Islands: CEPROS, traps, Radiale 2, Ech. 34, 04°22.5'S, 56°19.1'E, 200–190 m, 21–22 Oct 1987, coll. A Intes: 1 male 17.3 mm (MNHN-IU-2014–9400). Madagascar: CREVETTIERE 1971, N Madagascar, [Mozambique Channel], sta CH 11, 12°40'S, 48°15'E, 375–385 m, 14 Apr 1971: 2 males 12.4, 20.6 mm, 1 female 17.2 mm (MNHN-IU-2014–9394, = MNHN-Pg 1865); CREVETTIERE 1972, sta CH 32, 12°34'S, 48°18'E, 310–320 m, 13 Sep 1972: 2 males 17.1, 19.6 mm (MNHN-IU-2014–9393, = MNHN-Pg 1864). Mozambique Channel: Mozambique: RV *Algoa*, Mozambique Scad Survey SFRI, sta C00815–014–012–2144, 23°07.98'S, 35°42.00'E, 180 m, trawl, 12 Dec 1994: 1 male 16.8 mm (SAMC MB-A041691); MAINBAZA, NO *Vizconde de Eza*: Inhambane transect, sta CC 3159, 23°55'S, 35°37'E, 148–152 m, 15 Apr 2009, colls. P Bouchet, J Rosado & E Strong: not examined, color photograph (Fig. 8C) (MNHN). KwaZulu-Natal, South Africa: sta NAD11H, 29°46.02'S, 31°16.98'E, 110–130 m, 23 Apr 1958, coll. University of Cape Town Ecological Survey: 1 male 15.0 mm [det. KH Barnard] (SAMC MB-A019489); off Durban, KwaZulu-Natal, Oceanographic Research Institute ORI 68, sta ACEP 1–4, 29°58.56'S, 31°04.98'E, 119 m, trawl, 18 Feb 2010: 1 ovig female 14.4 mm (ZRC 2013.0535); off Durban, KwaZulu-Natal, Oceanographic Research Institute ORI 17, sta ACEP 4–1, 29°06.60'S, 32°07.32'E, 128 m, trawl, 20 Feb 2010: 1 male 14.2 mm (ZRC 2013.0537); Aliwal outer reef, off KwaZulu-Natal, DST/NRF ACEP, RY *Angra Pequena*, sta R50, 30°12.36'S, 30°59.16'E, 106–149 m, 5 Jun 2017, ROV: specimen not collected, color photograph *in situ* (Fig. 28B); Coffee Bay (Eastern Cape), 31°59.34'S, 29°09.96'E, 100 m, dredge, 10 Sep 2016: 1 male 24.1 mm (SAMC MB-A066723).

[*Locality uncertain*]: INVMAR: sta 17, 155–165 m, 3 Aug 1964, coll. OT Chan: 1 ovig female 11.2 mm (MNHN-IU-2014–9412, = MNHN-Pg 1828).

##### Redescription.

**Figure 9.**
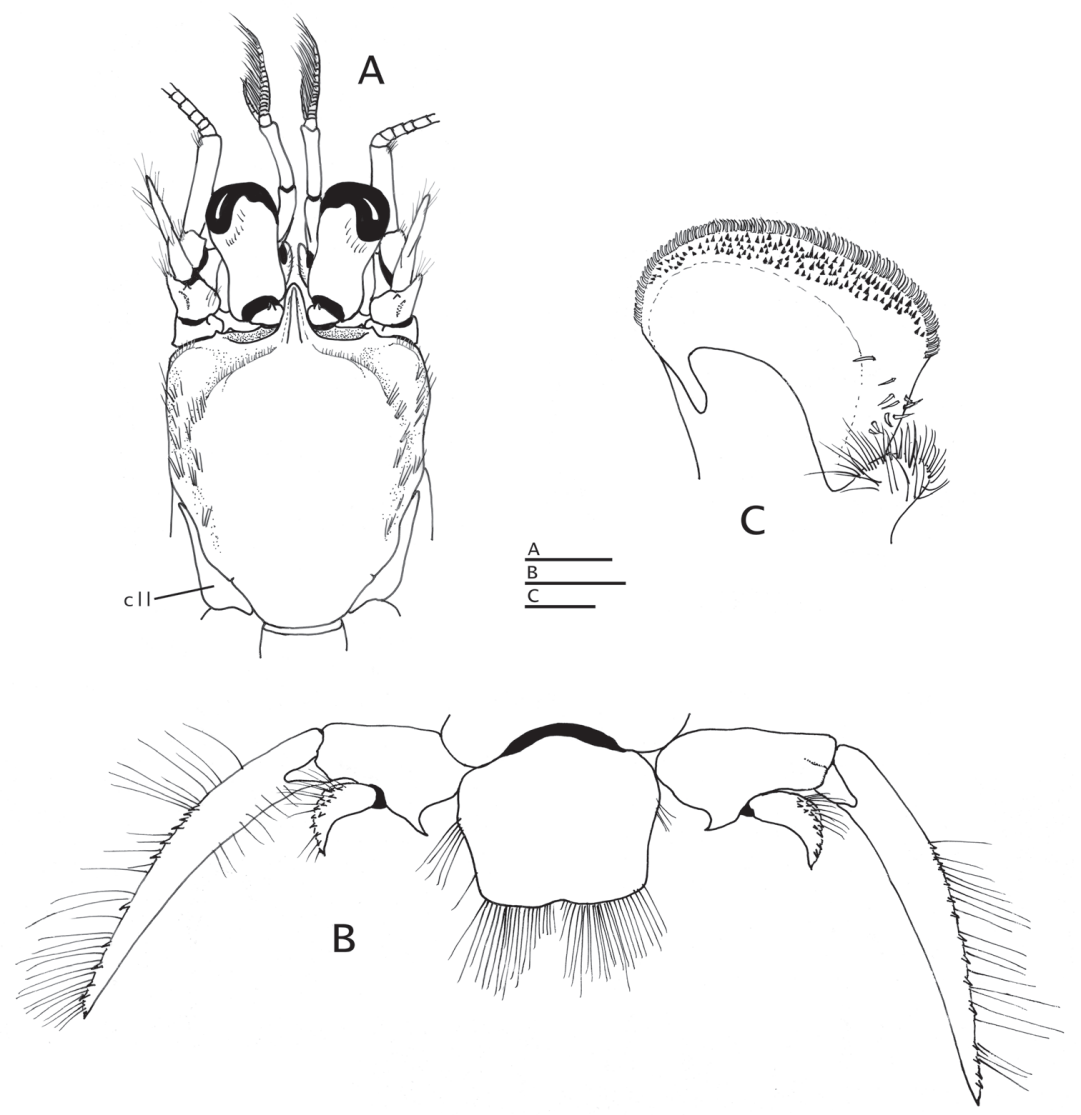
*Paguropsis andersoni* (Alcock, 1899) lectotype male, 18.3 mm, Laccadive Sea, Indian Ocean, HM Indian Marine Survey Steamer *Investigator* (USNM 42719). **A** shield and cephalic appendages, dorsal **B** uropods and telson, dorsal **C** distal portion of left gonopod 2, anterior. Abbreviations: cll, carapace lateral lobe. Scale bars: 5 mm (**A**), 3 mm (**B**), 0.5 mm (**C**).

*Shield* (Figs 2C, 8C, 9A) subtriangular, ca. 1.3 times as long as broad; dorsal surface distinctly rounded, somewhat vaulted, glabrous except for tufts of setae anterolaterally and transverse fringe of short setae on sloping anterior margins of gastric region; anterior margin between rostrum and lateral projections concave; lateral projections broadly triangular, each terminating in small spine; posterior margin roundly truncate; lateroventral distal angle with strong blunt spine near proximal margin of first antennal segment. Rostrum (Fig. 9A) bluntly or sharply triangular, dorsally arched, strongly produced, extending beyond distal margin of ocular acicles, fringed by short marginal setae; with distinct rounded dorsal longitudinal ridge having row of short setae laterally, ending smoothly or in 1 minute subterminal spine. Branchiostegites (Fig. 2D) unarmed except for 1–3 spines on dorsodistal angle, distal margin setose; anterodorsal plate calcified, narrow.

*Ocular peduncles* ca. 0.4 length of shield, constricted medially and noticeably broadened distally, glabrous except for scattered short dorsodistal setae; corneas strongly dilated, diameter 0.5–0.6 total peduncular length (including the cornea). Ocular acicles small, triangular, each armed with distal or dorsodistal spine often directed anterodorsally.

*Antennular peduncles* when fully extended overreaching distal margins of corneas by 0.2 length of penultimate peduncular segments. Ultimate and penultimate segments glabrous or at most with scattered short setae. Basal segment with ventromesial tuft of setae distally; lateral face with distal subrectangular lobe, small medial spine, and setose lobe proximally.

*Antennal peduncles* overreaching distal corneal margins by ca. 0.5 length of fifth segment. Fifth and fourth segments unarmed except for scattered short setae and laterodistal tuft of long setae. Third segment with spine at ventrodistal angle. Second segment with dorsolateral distal angle produced, terminating in small simple or less frequently, bifid spine; mesial margin rounded, setose, and small spine on dorsomesial angle. First segment unarmed except for moderately long setae on lateral face. Antennal acicle length variable with growth, reaching from distal margin of optic calathus to slightly exceeding distal margin of cornea, slender, terminating in sharp spine, with long setae distally, at most with 1 or 2 minute proximal tubercles on mesial margin. Antennal flagellum long, reaching to distal end of cheliped fingers, articles with 1 or 2 short setae (< 1 article in length) and usually with 1 or 2 long setae every 12 articles or so.

*Mouthparts* not markedly different from those described for *Paguropsis typica* (e.g., Fig. 4A–F). Maxilliped 3 with exopod ca. 4.2 times as long as broad.

*Chelipeds* (Figs 1E, 2C, 5C, 8C) subequal, similar in armature and setation; dorsal surfaces of chelae and carpi densely covered with tufts of long, bristle-like setae obscuring spination below, often with areas of short dense plumose setae on dorsal faces of dactyl, fixed finger, and palm; ventral surfaces of palms smooth except for 2 submedian longitudinal rows of well-spaced low tubercles each with tuft of long bristle-like setae. Dactyl and fixed finger with narrow hiatus proximally, forming spoon-like shape in ventral view when closed; each finger terminating in small curved corneous claw and subdistal blunt calcareous tooth ventral to claw, both claws and teeth interlocking when fingers closed; cutting edge of dactyl with terminal row of small, fused corneous teeth on distal one-third, and row of unequal calcareous teeth on proximal two-thirds; cutting edge of fixed finger with row of blunt calcareous teeth decreasing in size distally. Dactyl ca. 1.4 times as long as palm; dorsal surface somewhat convex, armed with small spines proximally and patch of dense, short plumose setae proximally and extending to mesial face; dorsomesial margin rounded; ventral face with well-spaced tufts of long bristle-like setae, lacking spines. Fixed finger with dorsal, lateral, and ventral surfaces similar to dactyl in armature. Palm ca. 0.6 times as long as carpus, dorsal surface covered with numerous small spines arranged in irregular longitudinal rows and accompanied by tufts of long, setae, strength and number of spines increasing with growth; dorsomesial margin with 2 or 3 rows of strong, well-spaced spines and tufts of long setae; dorsolateral margin rounded, not delimited, with irregular rows of small tubercles or spines, each accompanied by tuft of long setae. Carpus ca. 0.6 times length of merus; dorsal and dorsolateral surfaces with well-spaced spines often bifid or trifid and accompanied by tufts of long bristle-like setae; dorsomesial margin with row of strong spines accompanied by tufts of bristle-like setae, and dorsodistal spine; dorsolateral margin rounded; mesial surface with short transverse rows of bristle-like setae, otherwise smooth; ventral surface smooth except for fringe of long setae on ventrodistal margin extending onto mesial surface. Merus nearly as long as chela, subtriangular in cross-section; dorsal margin with row of protuberances accompanied by tufts of long setae, ventromesial and ventrolateral margins each with irregular row of strong spines with tufts of long setae; lateral and mesial surfaces with tufts of long and short setae. Ischium with row of small spines on ventrolateral margin. Basis with ventromesial row of long setae.

**Figure 10.**
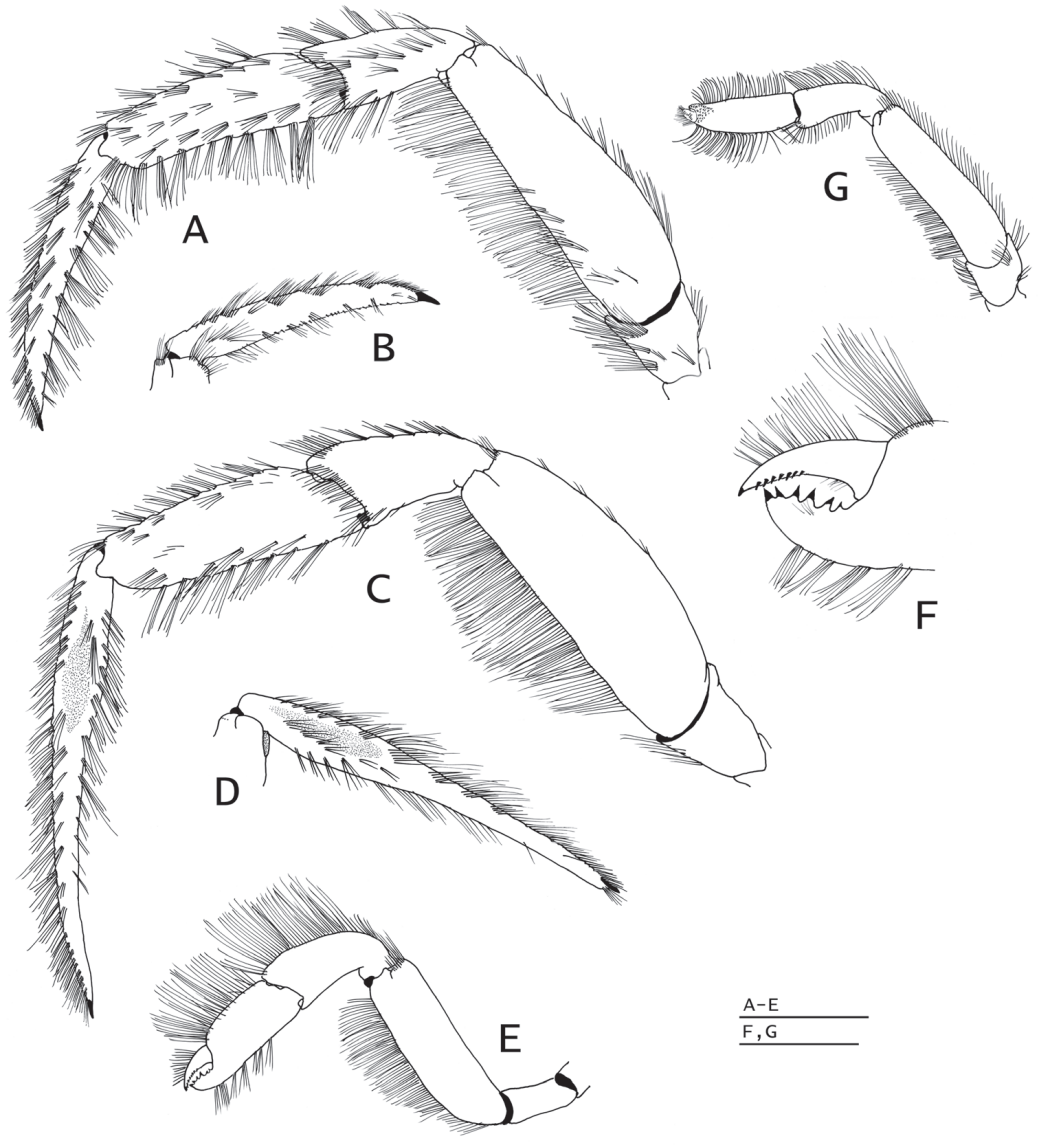
*Paguropsis andersoni* (Alcock, 1899), lectotype male, 18.3 mm, Laccadive Sea, Indian Ocean, HM Indian Marine Survey Steamer *Investigator* (USNM 42719). **A** left pereopod 2, lateral **B** dactyl of same, mesial **C** left pereopod 3, lateral **D** dactyl of same, mesial **E** left pereopod 4, lateral **F** dactyl and fixed finger of same, lateral **G** left pereopod 5, lateral. Scale bars: 10 mm (**A–E, G**), 3 mm (**F**).

*Pereopods 2 and 3* (Fig. 10A–D) similar in armature and setation, distinctly dissimilar in length, with pereopod 2 shorter than pereopod 3. Dactyls ca. 1.5 (pereopod 2) or 2.2 (pereopod 3) times as long as propodi; with dorsal and ventral margins, lateral and mesial surfaces, with numerous tufts of long, bristle-like setae; dactyl of pereopod 2 weakly curved, lateral surface convex, ventromesial distal margin armed with 10–19 minute corneous spinules; dactyl of pereopod 3 relatively broad on proximal one-third, becoming slender distally, terminating in sharp corneous claw, 1.5–1.6 times as long as dactyl of pereopod 2, lateral and mesial surfaces with shallow but distinct concavity (often weakly calcified) on proximal one-third, ventral margin lacking spines or spinules. Propodi ca. 1.2 times as long as carpi; dorsal and ventral surfaces with tufts of long setae. Carpi unarmed except for tufts of setae dorsally and distolateral fringe of long setae. Meri unarmed except for fringe of long setae ventrally and ventrolaterally (pereopod 2) or ventrally (pereopod 3). Ischia armed with row of small spines and setae (pereopod 2) or unarmed except for row of setae (pereopod 3). Coxae with ventromesial row of setae; coxae of pereopods 3 narrowly separated by ca. 0.2 ventral length of 1 coxa. Sternite XI (of pereopods 3; Fig. 5D) having anterior lobe flat to slightly concave, glabrous or with scattered short setae distally; posterior lobes strongly compressed laterally, each with transverse fringe of setae.

*Pereopod 4* (Fig. 10E, F) with chela short, 1.1 times as long as carpus and 2.5 times as long as high. Dactyl and fixed finger leaving wide gap when closed, each terminating in sharp, inwardly curved corneous claw crossing at tips when closed. Dactyl curved inwardly, dorsal margin with row of setae; cutting edge of dactyl with ventrolateral distal row of 6–8 small corneous-tipped spines (in addition to corneous claw). Fixed finger curving inward, cutting edge with 4 or 5 strong corneous-tipped spines (in addition to corneous claw) arranged like bear claw; lateral face usually with 1–4 minute scale-like corneous spines basally. Palm straight or slightly curved, 1.6–1.8 times as long as high; with dense fringe of long setae on dorsal margin, and tufts of setae on ventral margin continued on fixed finger; carpus unarmed except for fringe of long setae dorsally and scarce setae ventrally; merus long, ca. 0.6 times as long as meri of pereopods 2 and 3. Sternite XII broad, with dense fringe of long dense setae (Fig. 5D).

*Pereopod 5* (Fig. 10G) with chela ca. 0.6 times as long as merus, with long, brush-like setae on dorsomesial and ventromesial surfaces; merus and carpus each with dorsal and ventral row of long setae. Dactyl with rasp on ventral face. Propodal rasp consisting of minute, ovate scales, occupying 0.2 length of propodus. Ischium with setae dorsally and ventrally. Coxa with fringe of long bristle-like setae on rounded ventromesial distal angle.

*Male* gonopod 1 (Fig. 9C) with inferior lamella armed on distal margin with posterior row of slender, semitransparent hook-like spines, and up to 4 or 5 anterior irregular rows of small, straight or slightly curved corneous spines. Gonopod 2 with distal segment strongly twisted distally, densely setose; usually with unpaired, biramous or uniramous, reduced pleopods 3–5 on left side or less frequently on right side; often lacking pleopods 4 or 5 or lacking altogether pleopods 3–5 (see “Variations”).

*Female* usually with pleopods 2–5 on left side or less frequently on right side, as follows: pleopods 2–4 biramous, well developed, and reduced biramous or uniramous and vestigial pleopod 5 (see “Variations”). Brood pouch large, subquadrate, distal margin scalloped and fringed with setae.

*Uropodal exopods* (Fig. 9B) slender, broadly curved, terminating in strong spine, anterior margin with fringe of long setae and row of well-spaced corneous-tipped spines; endopods short, strongly curved, anterior margin with long setae and 1 or 2 irregular rows of corneous-tipped spines; protopods with strong, curved proximal spine.

*Telson* (Fig. 9B) subrectangular, wider than long; posterior lobes separated by shallow median cleft, terminal margins unarmed except for fringe of long setae.

##### Genetic data.

See Table 1.

##### Color

(Fig. 8C, 28B). Shield and calcified dorsal portions of posterior carapace orange. Ocular peduncles orange dorsally, white otherwise; corneas black. Ocular acicles light orange. Antennular peduncles and flagella light orange. Antennal peduncles light orange, with color fading to cream or whitish proximally on first to fourth segments. Chelipeds red or orange with white spines and tubercles and yellow bristle-like setae; chelae orange; carpi red; meri dorsal margin red extending to lateral and mesial faces subdistally, dorsodistal margin and most of lateral and mesial surfaces light orange. Pereopods 2 and 3 as follows: dactyl red with white dorsal margin; propodi red with white dorsal margin and scattered small white spots ventrolaterally; carpi red with large white portions on lateral face medially and distally; meri mostly white with reddish dorsal and ventral margins and on lateral face distally; ischium reddish. Pereopods 4 and 5 red or orange.

In explaining the etymology of his genus name *Chlaenopagurus* where this species was originally placed, Alcock (1899) mentioned that the purple coloration of the polyps that he interpreted to be a colonial zoanthid used by this species, was similar to the *chlaina* (Gr.), a mantle used in Homeric times for protection against the weather.

##### Distribution.

Western Pacific: from Philippines and Indonesia (Arafura Sea). Indian Ocean: from Gulf of Martaban, Andaman Sea (Alcock 1905); India, including eastern (Sarojini and Nagabhushanam 1972) and western (Thomas 1989) coasts; Seychelles Islands; eastern Africa, off Kenya; off Madagascar on Mozambique Channel; and eastern coast of South Africa. Depth: 30 to 548.6 m.

##### Habitat and symbiont.

Found with indeterminate species of acontiate anemone (see “Remarks” under genus).

##### Variations.

The following morphological features increase with size: length of antennal acicle; density and strength of spines on chelipeds; and density of tufts of setae on chelipeds and ambulatory legs. Larger males have more numerous subdistal spines or rows of spines on the distal margin of gonopod 1 (Fig. 9C).

Of the four females examined, one has pleopods 2–5 on the left side, and the other on the right side; both have pleopod 5 reduced. Of the 29 males examined, 53.8% had one or more unpaired pleopods 3–5 on the left side, 15.4% on the right side, 7.7% did not have any unpaired pleopods on either side, and 23.1% had paired pleopods 3–5. As in males of *P. typica*, the male pleopods 3–5 are reduced, uni- or biramous.

##### Affinities.

As previously mentioned under the “Remarks” of *Paguropsis typica*, *P. andersoni* can be distinguished primarily from that species and other congenerics by the distinct longitudinal concavity present on the lateroproximal surface of the dactyls of pereopods 3 (second ambulatory legs). In addition, the coxae of pereopods 3 (Fig. 5D) are noticeably more narrowly separated (by ca. 0.2 ventral length of 1 coxa) from each other than in other congeners, and the posterior lobes of sternite XI are noticeably compressed. Other characters setting *P. andersoni* apart, albeit subject to some variability related to size, include the more numerous and stronger spination of the chelipeds, particularly on the dorsal surface of carpus, chela, ventromesial and ventrolateral margins of merus; the dense tufts of setae on chelipeds and meri to dactyls of ambulatory legs; and pereopod 4 with a shorter chela relative to the carpus length.

Coloration is clearly distinct in *Paguropsis andersoni* when compared to other congeners as well as with all other species discussed herein (see Figs 8, 18, 28). The overall color of *P. andersoni* (Figs 8C, 28B) does bear some general similarity with that of *P. typica* (Figs 8A, B, 28A); however, the ocular peduncles, chelae, and ambulatory legs in each of these two species have a different pattern. In *P. andersoni* the ocular peduncles are light orange dorsally, and white otherwise (vs. light orange overall with a darker orange band in *P. typica*), the chelae are mostly orange (vs. mostly whitish in *P. typica*), and the propodus and dactyl of pereopods 2 and 3 are mostly solid red except for white dorsal margins of dactyls (vs. reddish mottled with white spots, and a median longitudinal white line on the lateral surface of the propodus, in *P. typica*).

##### Remarks.

Alcock (1905) concluded, without providing any explanation, that his genus and species described earlier as *Chlaenopagurus andersoni* Alcock, 1899, were the same as Henderson’s (1888) genus and species *Paguropsis typica*, and thus placed his name in the synonymy of the latter. Alcock’s synonymy has stood since that time. However, detailed study of types and specimens deposited in various museums, as well as recently collected material of *Paguropsis*, has shown that Alcock’s name actually represents a valid and morphologically distinct species, and as such is resurrected herein with a lectotype selected from the syntype series. Alcock (1905: 30) described the coloration of *P. typica* and its cnidarian symbiont, as follows “The colour of the crab is red: the coenosarc of the polyp-colony is bluish, the polyps themselves are dark purple”, actually is applicable as well to *P. andersoni*.

When Alcock (1899) described *Chlaenopagurus andersoni* he did not designate a holotype among his numerous specimens, and thus all are syntypes, several of which were distributed to major museums such as the USNM, BMNH, and ZMUC. A lectotype is herein selected for Alcock’s taxon from the syntypes exchanged with the USNM. The labels with the syntype specimens sent to USNM lacked a station number, but based on all other information accompanying these syntypes and comparing them with Alcock’s (1902) detailed station data, it seems clear that they came from *Investigator* Survey station 258.

Several studies have reported new specimens collected since Henderson’s (1888) description, as *Paguropsis typica*. These are as follows: Alcock (1905), from the Gulf of Martaban (Andaman Sea); Balss (1927), from the “Indian Ocean”; Thompson (1943, spelled both *P. typicus* and *P. typica*), from Zanzibar; Barnard (1962), from East Africa (Natal); and Sarojini and Nagabhushanam (1972), from Waltair (eastern India, Bay of Bengal). Based on information therein, and in light of the finding during this study that Henderson’s (1888) *P. typica* has not been found outside the western Pacific, these reports are herein referred to *P. andersoni*. The lists or catalogues of hermit crabs from southern Africa by Kensley (1981) and Emmerson (2016) that have included *P. typica* in the fauna from that region have been based on Barnard’s (1962) original male specimen (SAMC MB-A019489) which is shown here to actually be *P. andersoni*.

As mentioned under the “Remarks” for *Paguropsis typica*, a number of reports (Balss 1924, 1956, Gordan 1956) that have used that name actually represent, in part, *P. andersoni*, as does the distribution map used by Schäfer et al. (1983).

In a summary of the hermit crabs from the Indian Museum, Kamalaveni (1950) based his discussion of *Paguropsis typica* (spelled *P. typicus*) exclusively on the material used by Alcock (1899) in his description of *Chlaenopagurus andersoni*, a taxon shown here to be a valid species of *Paguropsis*. Thus, Kamalaveni’s report pertains entirely to *P. andersoni*.

#### Paguropsis confusa sp. n.

Taxon classificationAnimaliaDecapodaDiogenidae

http://zoobank.org/D5200836-E6C3-4033-A9FB-680981F9D5FC

**Figure 11.**
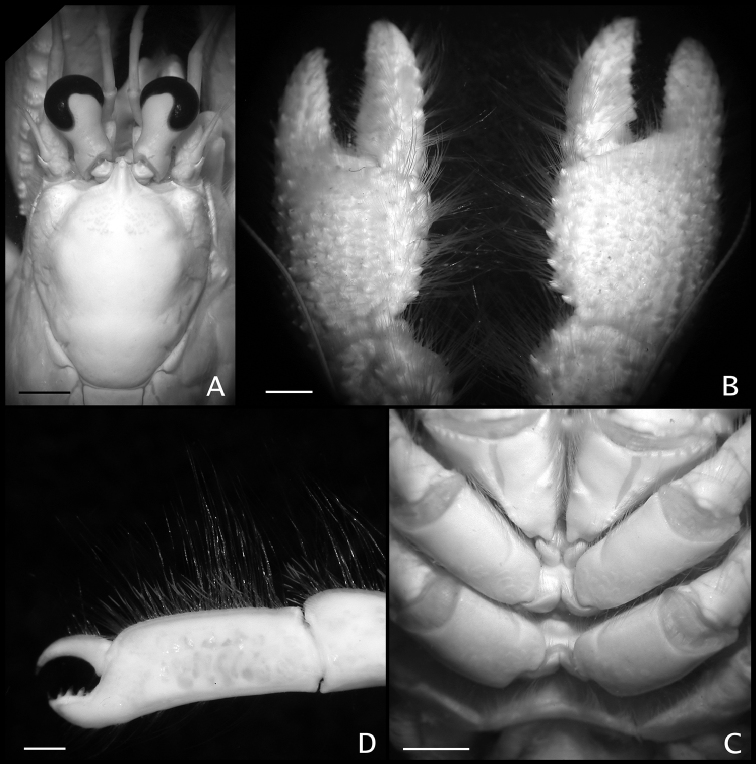
*Paguropsis confusa* sp. n., holotype male, 13.6 mm, Philippines, PANGLAO 2005, sta CP 2331 (MNHN 50015). **A** shield, cephalic appendages, and anterior portion of posterior carapace, dorsal **B** chelae, dorsal **C** coxae of pereopods 1–3, and sternites IX–XI, ventral **D** chela of left pereopod 4, lateral. Scale bars: 3 mm (**A–C**), 1 mm (**D**).

**Figure 12.**
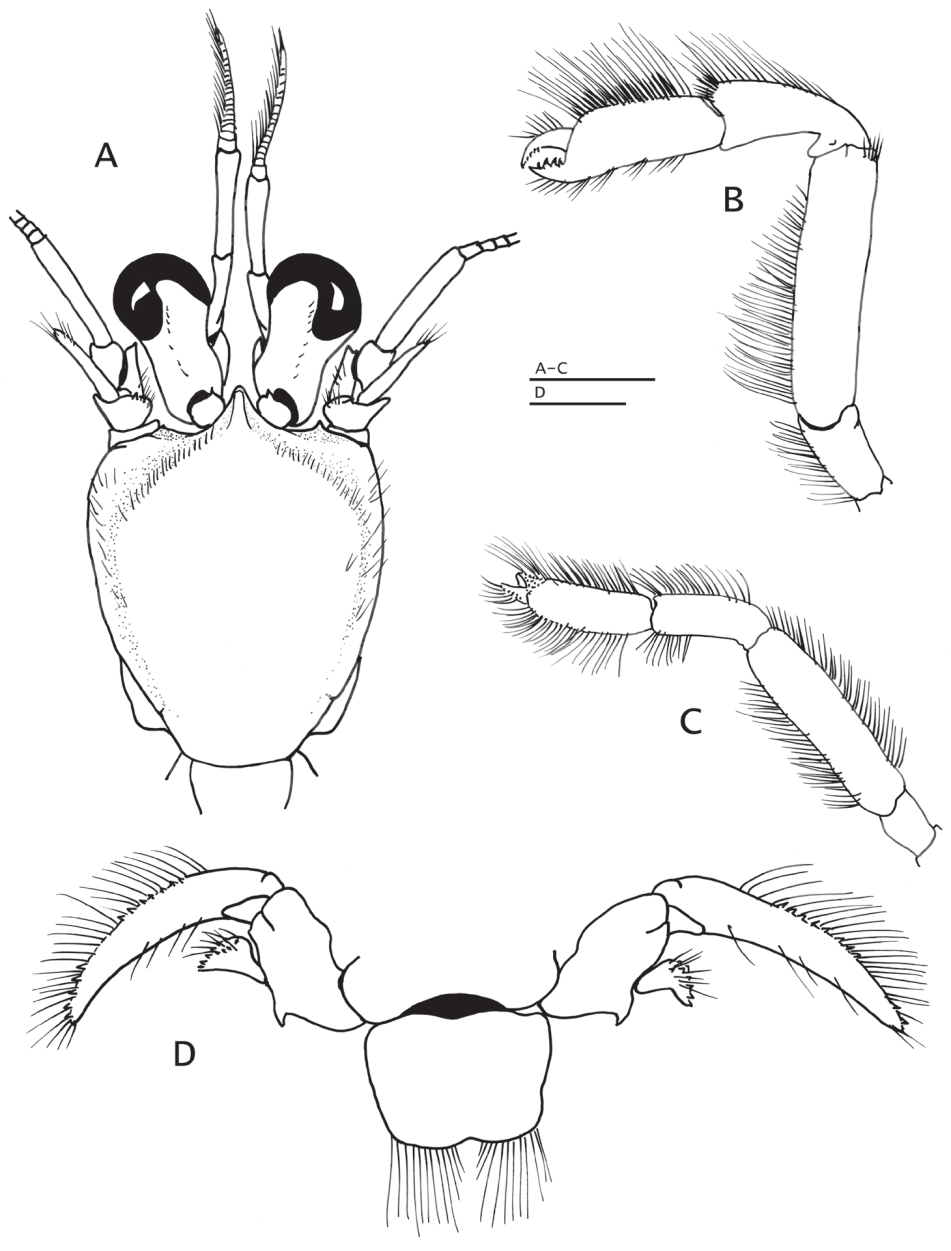
*Paguropsis confusa* sp. n., holotype male, 13.6 mm, Philippines, PANGLAO 2005, sta CP 2331 (MNHN 50015). **A** shield and cephalic appendages, dorsal **B** left pereopod 4, lateral **C** left pereopod 5, lateral **D** uropods and telson, dorsal. Scale bars: 5 mm (**A–C**), 2 mm (**D**).

**Figure 13.**
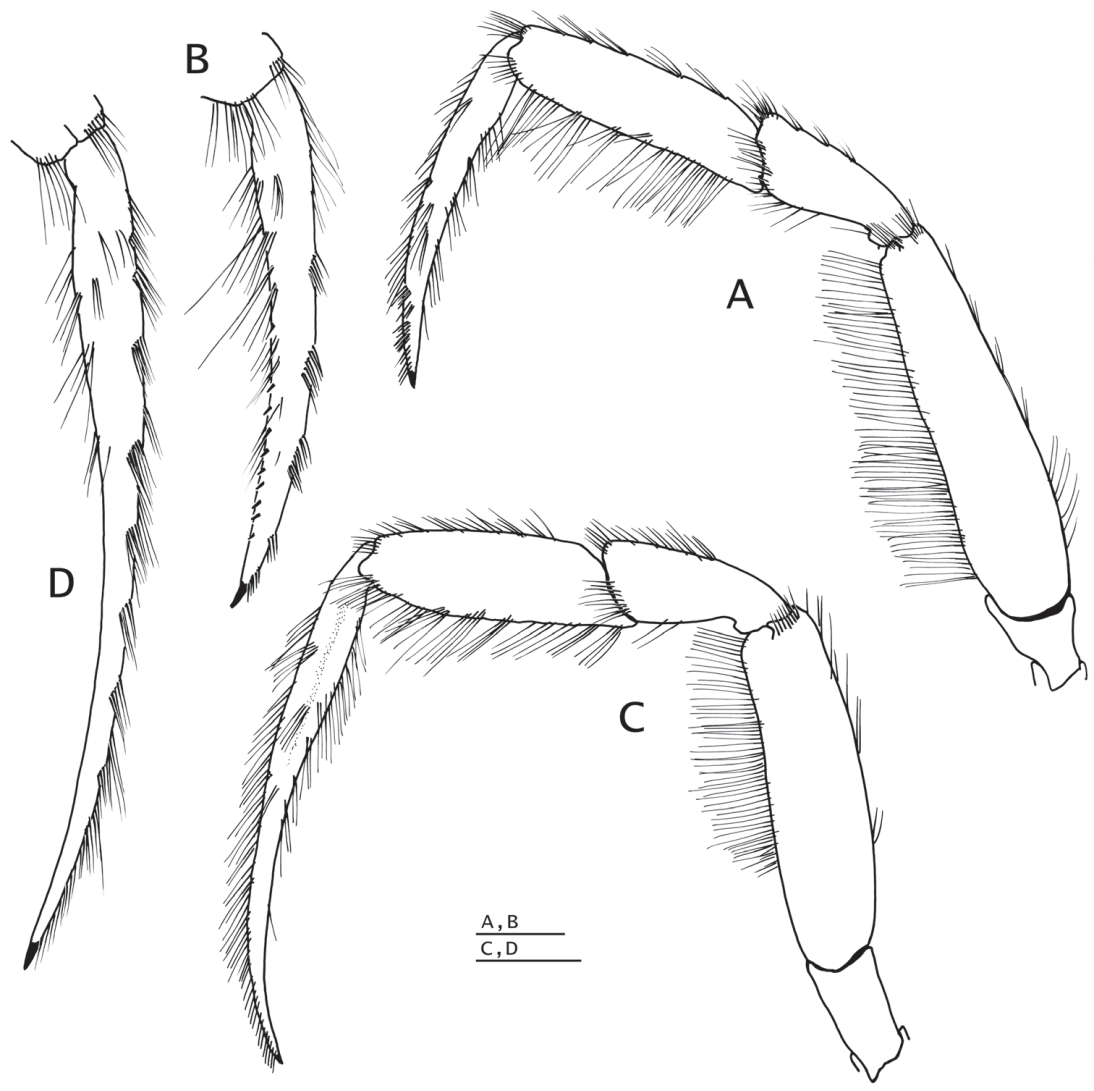
*Paguropsis confusa* sp. n., Philippines, holotype male 13.6 mm, PANGLAO 2005, sta CP 2331 (MNHN 50015). **A** left pereopod 2, lateral **B** dactyl of same, mesial **C** left pereopod 3, lateral **D** dactyl of same, mesial. Scale bars: 5 mm (**A, B**), 3 mm (**C, B**).

**Figure 14.**
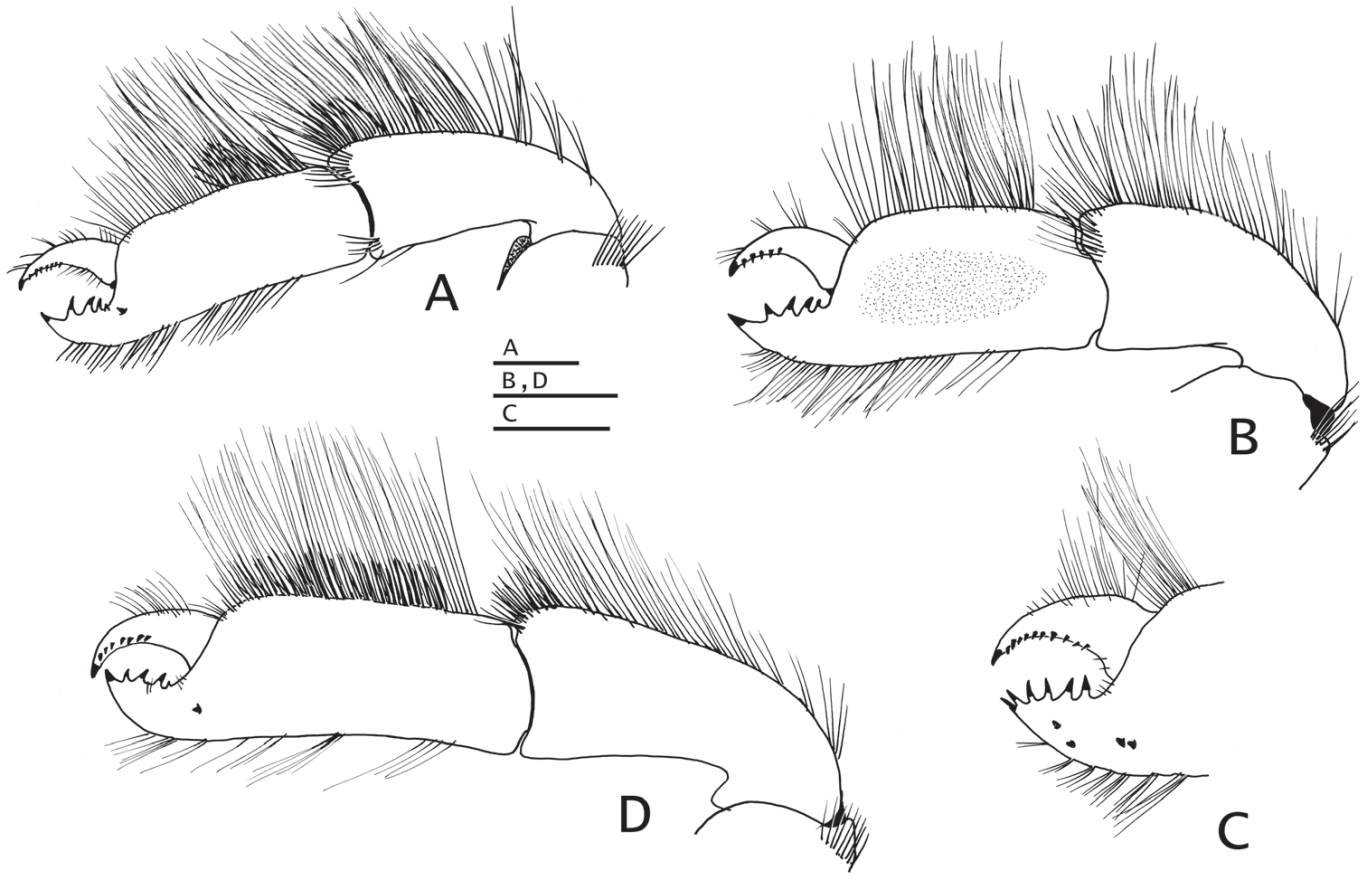
**A** *Paguropsis typica* Henderson, 1888, male, 7.9 mm, MUSORSTOM 3, sta CP 90 (MNHN-IU-2014–9411) **B, C** *P. andersoni* (Alcock, 1899): B ovig female, 11.2 mm, [Locality uncertain], INVMAR, sta 17 (MNHN-IU-2014–9412), C male, 15.2 mm, Indonesia, KARUBAR, sta CP 46 (USNM 1442005) **D** *P. confusa* sp. n., holotype male, 13.6 mm, Philippines, PANGLAO 2005, sta CP 2331 (MNHN 50015). **A, B, D** Left chela and carpus of pereopod 4, lateral **C** dactyl and fixed finger. Scale bars: 1 mm (**A**), 2 mm (**B, D, C**).

Figs 8D , 11 , 12 , 13 , 14D , 28C, D , Table 1

##### Type material.

Holotype, male 13.6 mm, Philippines, Bohol Sea, Maribojoc Bay, PANGLAO 2005, NO *DA-BFAR*, sta CP 2331, 09°39'N, 123°48'E, 256–268 m, 22 May 2005 (MNHN 50015).

##### Paratypes.

*Philippines*: MUSORSTOM 1, NO *Vauban*: N of Lubang, sta CC 12, 14°00'N, 120°17'E, 187–210 m, 20 Mar 1976: 5 males 9.7–11.2 mm, 3 females 7.9–9.7 mm, 2 ovig females 9.2, 10.4 mm (MNHN-IU-2014–9397). MUSORSTOM 3, NO *Coriolis*: W of Luçon, sta CP 90, 14°00'N, 120°19'E, 195 m, 31 May 1985: 9 males 6.9–10.3 mm, 1 female 9.7 mm (USNM 1441901); N Cebu Island, sta CP 143, 11°29'N, 124°11'E, 205–214 m, 7 Jun 1985: 1 male 20.9 mm (MNHN-IU-2014–9396); NE of Cebu Island, sta CP 145, 11°01'N, 124°04'E, 214–246 m, 7 Jun 1985: 4 males 9.8–13.5 mm, 2 ovig females 11.0, 11.7 mm (MNHN-IU-2014–9398). LUMIWAN 2008, NO *DA-BFAR*: N of Lubang, sta CP 2867, 14°02'N, 120°12'E, 189–93 m, 23 Mar 2008: sex indet, not examined, color photograph (Fig. 28D) (MNHN).

*South China Sea*: INVMAR, [off Vietnam], sta 69, Cr. 4/63, 15°55'44"N, 15°57'54"N, 109°8'30"E –109°36'30"E, 260–315 m, 16 Sep 1963: 3 males 7.6–11.8 mm (MNHN-IU–2014–9419, = MNHN Pg 2314). Hong Kong: Cr. 4163, sta 66, Tr. 56, coll. Hong Kong Fisheries Research Station, [no other data]: 1 female 6.4 mm (MNHN-IU-2013–5658).

*Indonesia*: CORINDON 2, NO *Coriolis*: Kalimantan, Makassar Straits, sta CH 208, 00°15'S, 117°52'E, 150 m, 31 Oct 1980: 1 male 10.3 mm (MNHN-IU-2014–9377).

*Western Indian Ocean*: MIRIKY: Madagascar, between Nosy-bé and Banc du Leven, sta CP 3188, 12°31'S, 48°22'E, 298–301 m, 27 Jun 2009, colls. P Bouchet, N Puillandre & B Richer de Forges: 2 specimens not examined, identified and sexed from color photographs, 1 female (#50), 1 ovig female (#51) (MNHN). MAINBAZA, NO *Vizconde de Eza*: Mozambique Channel, Maputo transect, sta CP 3134, 25°11'S, 35°14'E, 303–403 m, 10 Apr 2009, colls. P Bouchet, J Rosado & E Strong: sex indet. (specimen #23), not examined, color photograph (Fig. 8D) (MNHN). DST/NRF ACEP: off Durban, KwaZulu-Natal, South Africa, RY *Angra Pequena*, sta R45 Echinoderm Extravaganza, 29°52.80'S, 31°11.76'E, 215 m, 23 Jun 2016, ROV: specimen not collected, color photograph *in situ* (Fig. 28C).

[*Locality uncertain*]: INVMAR: sta 8, 5 Dec 1963 [no other data]: 1 male 10.3 mm (MNHN-IU-2014–9424, = MNHN-Pg 2312); sta 26, [no other data]: 4 males 5.5–11.8 mm, 1 female 7.1 mm (MNHN-IU-2014–9421, = MNHN Pg 2319); sta 28, [no other data]: 3 ovig females 8.1–11.3 mm (MNHN-IU-2014–9420, = MNHN Pg 2318); sta 64, 16 Sep 1963: 3 males 6.2–9.7 mm, 2 females 5.2, 7.3 mm (MNHN-IU-2014–9417, = MNHN Pg 2317).

##### Description.

*Shield* (Figs 8D, 11A, 12A) subtriangular, ca. 1.3 times as long as broad; dorsal surface glabrous except for anterolateral setae and transverse fringe of short setae on sloping anterior margins of gastric region; anterior margin between rostrum and lateral projections concave; lateral projections broadly triangular, each terminating in small spine; posterior margin roundly truncate; lateroventral distal angle produced into strong blunt spine adjacent to proximal margin of first antennal segment. Rostrum (Figs 8D, 11A, 12A) acutely subtriangular, arched dorsally, strongly produced and extending to distal margin of ocular acicles; with distinct rounded dorsal longitudinal ridge having few short setae laterally, and 1 minute subterminal spine. Branchiostegite with 1 spine on dorsodistal angle of anterodorsal plate, and setose distal margin.

*Ocular peduncles* ca. 0.4 length of shield, constricted medially and broadening distally, glabrous except for dorsal longitudinal row of short setae; corneas strongly dilated, diameter 0.5–0.6 total peduncular length (including the cornea). Ocular acicles small, triangular, each armed with distal spine directed anteriorly.

Antennular peduncles when fully extended overreaching distal margins of corneas by full length of ultimate peduncular segment; ultimate and penultimate segments glabrous or at most with scattered short setae; basal segment with ventromesial tuft of setae distally; lateral face with distal subrectangular lobe, small medial spine, and setose lobe proximally.

*Antennal peduncles* overreaching distal corneal margins by ca. 0.3 length of ultimate segments. Fifth and fourth segments unarmed, nearly glabrous except for scattered short setae. Third segment with setose spine at ventrodistal angle. Second segment with dorsolateral distal angle produced, terminating in small simple or bifid spine; mesial margin rounded, setose, dorsomesial distal angle with small spine. First segment unarmed except for setae on lateral face. Antennal acicle relatively short, reaching at most to distal margin of optic calathus, slender, nearly straight and terminating in sharp spine, with few long setae distally. Antennal flagellum long, reaching to distal end of cheliped fingers, with few, scattered short setae less than 1 flagellar article in length.

*Mouthparts* not markedly different from those described for *Paguropsis typica* (see Fig. 4A–F). Maxilliped 3 with exopod ca. 4.1 times as long as broad.

*Chelipeds* (Figs 8D, 11B) subequal, similar in armature and setation; dorsal surfaces of chelae and carpi covered with moderately dense tufts or short rows of long, bristle-like setae not hiding ornamentation beneath; ventral surfaces of palms smooth except for 2 submedian longitudinal rows of well-spaced low tubercles each with tuft of long bristle-like setae. Dactyl and fixed finger with narrow hiatus proximally when closed, forming spoon-like shape in ventral view when closed; each terminating in small curved corneous claw and subdistal blunt calcareous tooth ventral to claw, both claws and teeth interlocking when fingers closed; cutting edge of dactyl with terminal row of small, fused corneous teeth on distal one-third, and row of unequal calcareous teeth on proximal two-thirds; cutting edge of fixed finger with row of blunt calcareous teeth decreasing in size distally. Dactyl ca. 1.2 times as long as palm; dorsal surface convex, with numerous tufts of long bristle-like setae, and few small blunt spines proximally; dorsomesial margin rounded, usually with patch of short dense plumose setae medially; ventral face with well-spaced tufts of long bristle-like setae inserted at bases of small tubercles, lacking spines. Fixed finger with dorsal, lateral, and ventral surfaces similar to dactyl in armature. Palm as long as carpus, dorsal surface covered with numerous small spines arranged in more or less irregular longitudinal rows of spines with tufts of long setae; dorsomesial margin with row of 6–8 spines with tufts of long setae; dorsolateral margin rounded, not delimited, with irregular rows of small tubercles or spines, each accompanied by long setae. Carpus 0.5–0.6 times length of merus; dorsal and dorsolateral surfaces with well-spaced spines or short transverse rows of 2 or 3 small spines each bearing tufts of long setae; dorsolateral margin rounded; dorsomesial margin with row of strong spines each bearing tufts of long setae; mesial surface with short transverse rows of bristle-like setae, otherwise smooth; ventral surface smooth except for fringe of long setae on ventrodistal margin extending on to mesial surface. Merus nearly as long as chela, subtriangular in cross-section; dorsal margin with row of low protuberances each bearing transverse row of 2 or 3 small tubercles and bearing tuft of long setae; ventromesial and ventrolateral margins each with irregular row of spines with tufts of long setae; lateral and mesial surfaces with tufts of long and short setae. Ischium with row of small spines on ventrolateral margin. Basis with ventromesial row of long setae. Coxa with well-marked longitudinal fissure (Fig. 11C) on ventral surface.

*Pereopods 2 and 3* (Figs 8D, 13A–D) similar in armature and setation, distinctly dissimilar in length, with pereopod 2 distinctly shorter than pereopod 3. Dactyls ca. 1.4 (pereopod 2) or 2.3 (pereopod 3) times as long as propodi, broadly curved, each terminating in sharp corneous claw; dorsolateral and dorsomesial margins with short oblique rows of bristle-like setae; dactyl of pereopod 3 becoming slender distally, ca. 1.4 times as long as dactyl of pereopod 2; lateral surface with proximal third having shallow or weakly distinct longitudinal concavity; ventromesial margin armed with 10–15 minute corneous spinules (pereopod 2) or unarmed (pereopod 3), and with tufts of long bristle-like setae. Propodi ca. 1.4 (pereopod 2) or 1.1 (pereopod 3) times as long as carpi; dorsal surfaces with short tufts of setae, ventral surfaces with tufts of long setae. Carpi unarmed except for tufts of setae dorsally and distolateral fringe of long setae. Meri with fringe of long setae on ventral margins; ventral margin of merus of pereopod 2 with or without row of small blunt spines (frequently lacking or fewer spines in specimens SL ≤ 11.0 mm) hidden by setae. Ischia armed with row of few small spines and long setae (pereopod 2) or unarmed and scattered setae (pereopod 3). Coxae with ventromesial margin sparsely setose; coxae of pereopods 3 (Fig. 11C) narrowly separated by 0.2 ventral length of 1 coxa. Sternite XI (Fig. 11C) having anterior lobe flat or weakly concave medially, glabrous; posterior lobes compressed, each with transverse fringe of setae.

*Pereopod 4* (Figs 12B, 14D) with chela as long as carpus and 3.2–3.6 times as long as high, palm 1.8–2.0 times as long as high. Dactyl and fixed finger leaving wide gap when closed, each terminating in sharp, inwardly curved corneous claw crossing when closed. Dactyl strongly curved inward, dorsal margin with row of short setae; cutting edge with ventrolateral distal row of up to 8 small corneous-tipped spines (in addition to corneous claw). Fixed finger curving inward, cutting edge with 3 or 4 strong corneous-tipped spines (in addition to corneous claw) arranged like bear claw; lateral face usually with 1–4 minute scale-like corneous spines near base of finger. Palm straight, ca. 2.1 as long as high; dorsal margin with 2 interspersed fringes of setae, 1 of short thick setae and 1 of long thin setae; ventral margin with tufts of setae continued sparsely on fixed finger. Carpus unarmed except for fringe of long setae on dorsal margin, and fringe of short thick setae on dorsodistal angle of lateral face; with scattered short setae ventrally. Merus 0.5–0.6 times as long as meri of pereopod 2 and pereopod 3, respectively. Sternite XII (Fig. 11C) weakly divided medially, each side with fringe of long dense setae.

*Pereopod 5* (Fig. 12C) with chela ca. 0.6 times as long as merus, with long, brush-like setae on dorsal and ventral surfaces; merus and carpus each with dorsal and ventral row of long setae. Dactyl with propodal rasp on ventral face. Propodal rasp consisting of minute, ovate scales, occupying 0.2 length of propodus. Ischium with setae dorsally and ventrally. Coxa with fringe of long bristle-like setae on rounded ventromesial distal angle.

*Male* gonopod 1 with inferior lamella armed on distal margin with posterior row of slender, semitransparent hook-like spines, and 2–4 anterior irregular rows of small, straight or slightly curved corneous spines. Gonopod 2 with distal segment strongly twisted distally, densely setose; usually with unpaired, reduced pleopods 3–5 on left side or less frequently on right side, as follows: biramous pleopods 3 and 4, and uniramous, vestigial pleopod 5 (see “Variations”).

*Female* with unpaired pleopods 2–5 on left side or less frequently on right side, as follows: pleopods 2–4 biramous, well developed, and reduced biramous or uniramous and vestigial pleopod 5 (see “Variations”). Brood pouch large, subquadrate, distal margin scalloped and fringed with setae.

*Uropodal exopods* (Fig. 12D) slender, broadly curved, terminating in strong spine, anterior margin with fringe of long setae and row of well-spaced corneous-tipped spines; endopods short, strongly curved, anterior margin with long setae and 1 or 2 irregular rows of corneous-tipped spines; protopods with strong, ventrally curved proximal spine.

*Telson* (Fig. 12D) subrectangular, wider than long; posterior lobes separated by weak shallow median cleft, terminal margins unarmed except for fringe of long setae.

##### Genetic data.

See Table 1.

##### Color

(Figs 8D, 28C, D). Shield light orange except for pink medial area just posterior to rostrum. Ocular peduncles light orange except for white proximal margins and white distal margins bordering black corneas; ocular acicles mostly light orange except for white distal margins and white distal spine. Antennules light orange, flagella of similar but darker color than peduncle. Antennal peduncles light orange, and similar but lighter toned and somewhat transparent flagella. Chelipeds with white spines and tubercles; chelae white except for light orange proximally on dorsal surface of palms and near bases of dactyls and fixed fingers; carpi orange; meri with reddish dorsal margins, and two short dark red stripes on distolateral and distomesial margins. Pereopods 2 and 3 with white except as follows: dactyls each with orange stripe on lateral face interrupted at proximal one-fifth; propodi each with two broad orange bands (fading dorsally and ventrally), one band on lateral face proximally and another distally; carpi each with orange portion dorsodistally, ventrally, and proximally; meri each with light orange dorsal and ventral margins, and short orange band laterodistally; ischium with light orange dorsal margin.

##### Etymology.

The specific name is derived from the Latin feminine singular of *confuso*, meaning confusion or disorder, and in reference to the state of morphological confusion that had prevented the unmasking of this and other new species under the name *P. typica*.

##### Distribution.

Western Pacific: from Philippines, South China Sea, and Indonesia (Kalimantan) on the Macassar Strait. Western Indian Ocean: Mozambique Channel to off Durban, South Africa. Depth: 150 to 403 m.

##### Habitat and symbiont.

Found with indeterminate species of acontiate anemone (see “Remarks” under genus).

##### Variations.

Among the 31 males and 16 females examined, 99% of males had pleopods 3–5 on the left side, and 37.5% of females had pleopods 2–5 on the left side. There is no other appreciable morphological variation other than that incorporated in the description.

##### Affinities.

This new species is superficially similar to *P. andersoni*, from which it differs drastically in coloration and several other characters. Generally, *P. confusa* sp. n. has a delicate morphology, in particular the less strongly armed chelipeds and slenderer pereopods 2 and 3 than in *P. andersoni* (see Figs 10A–D, 13A–D). The proximal one-third of the lateroproximal surface of the dactyl of pereopod 3 is weakly or indistinctly concave, whereas the lateroproximal surface is has a distinctly marked concavity in *P. andersoni*. The coxae of pereopods 3 are narrowly separated from each other in both species (by 0.2 ventral length of 1 coxa), but the posterior lobes of sternite XI (Figs 5D, 11C) are noticeably less compressed in *P. confusa* sp. n. than in *P. andersoni*. The discovery of the drastically different coloration patterns of these two species made possible, in part, the morphological separation of the two (compare Figs 8C, D, 28C, D).

#### Paguropsis gigas sp. n.

Taxon classificationAnimaliaDecapodaDiogenidae

http://zoobank.org/81B16E94-F229-40C9-93AE-4CBA72D252C7

**Figure 15.**
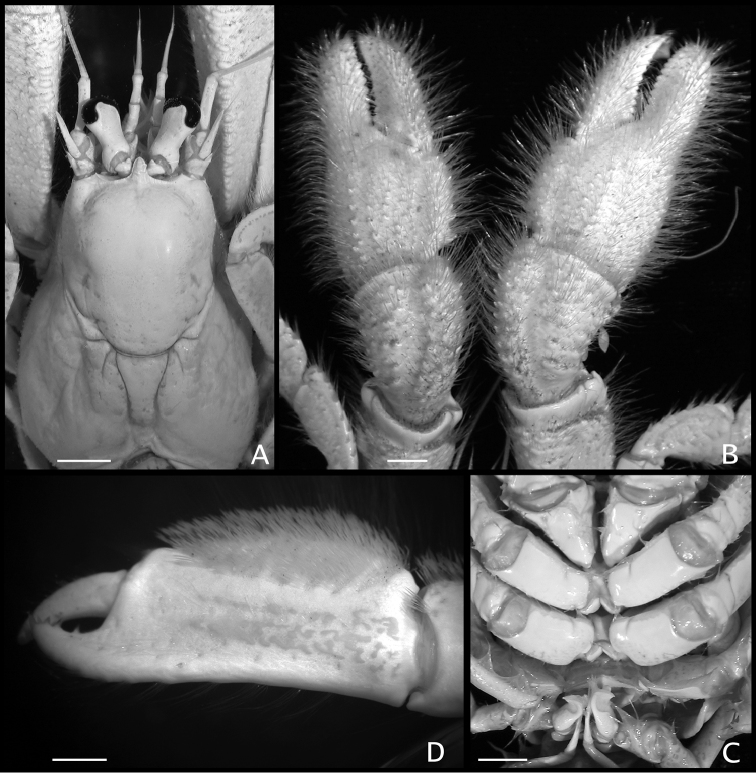
*Paguropsis gigas* sp. n., holotype male, 23.0 mm, South China Sea, TAIWAN, sta DW 4105 (NTOU A01445). **A** shield, cephalic appendages, and posterior carapace, dorsal **B** chelae and carpi of chelipeds, dorsal **C** coxae of pereopods 1–5, and sternites IX–XIII, ventral **D** chela of left pereopod 4, lateral. Scale bars: 6 mm (**A–C**), 2 mm (**D**).

**Figure 16.**
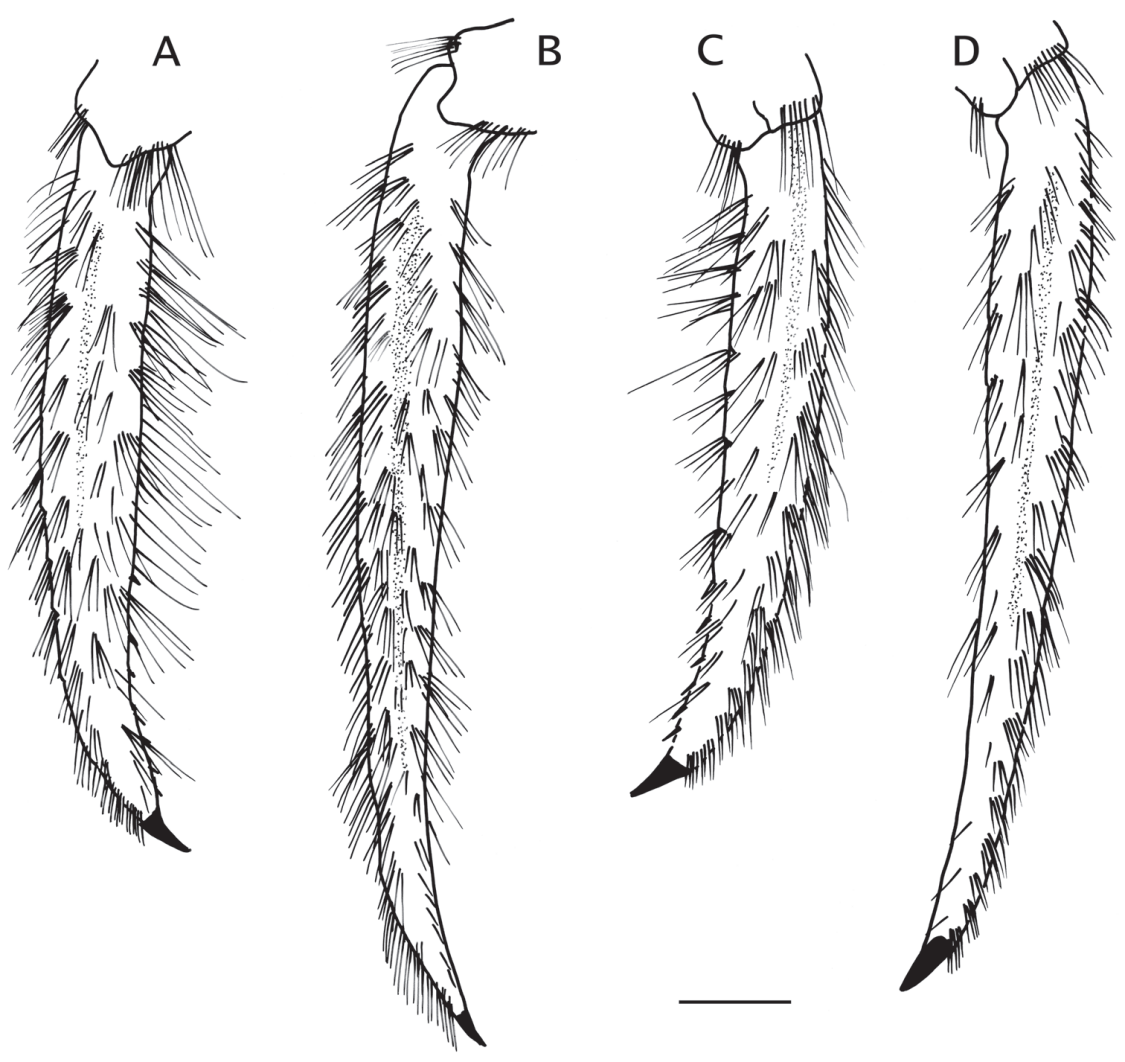
*Paguropsis gigas* sp. n., holotype male, 23.0 mm, South China Sea, TAIWAN, sta DW 4105 (NTOU A01445). **A, B** dactyl of right pereopod 2 (**A**) and pereopod 3, mesial (**B**) **C, D** dactyl of left pereopod 2 (**C**) and pereopod 3 (**D**), mesial. Scale bar: 5 mm.

**Figure 17.**
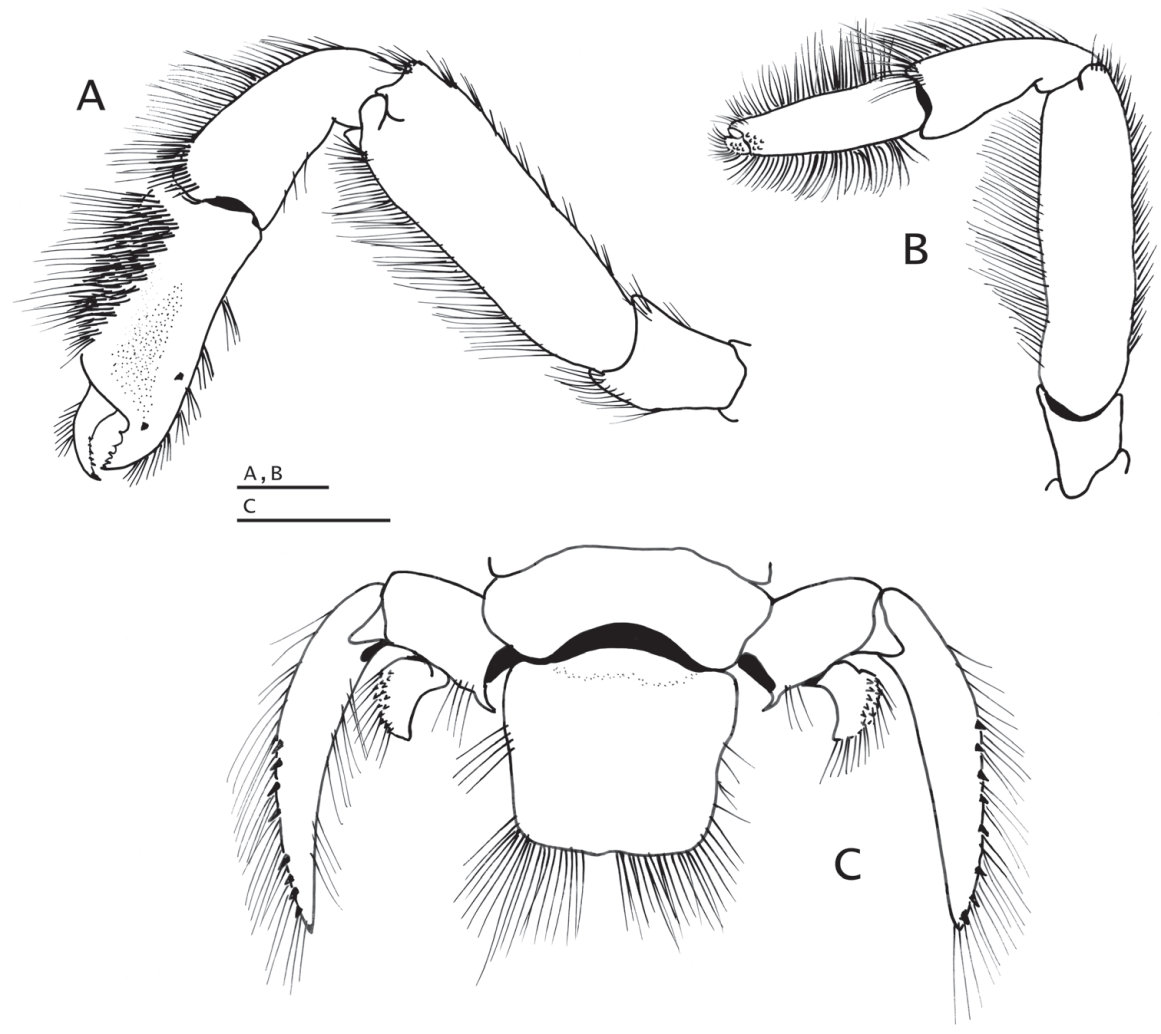
*Paguropsis gigas* sp. n., holotype male, 23.0 mm, South China Sea, TAIWAN, sta DW 4105 (NTOU A01445). **A** left pereopod 4, lateral **B** left pereopod 5, lateral **C** uropods and telson, dorsal. Scale bars: 1 mm (**A, B**), 3 mm (**C**).

**Figure 18.**
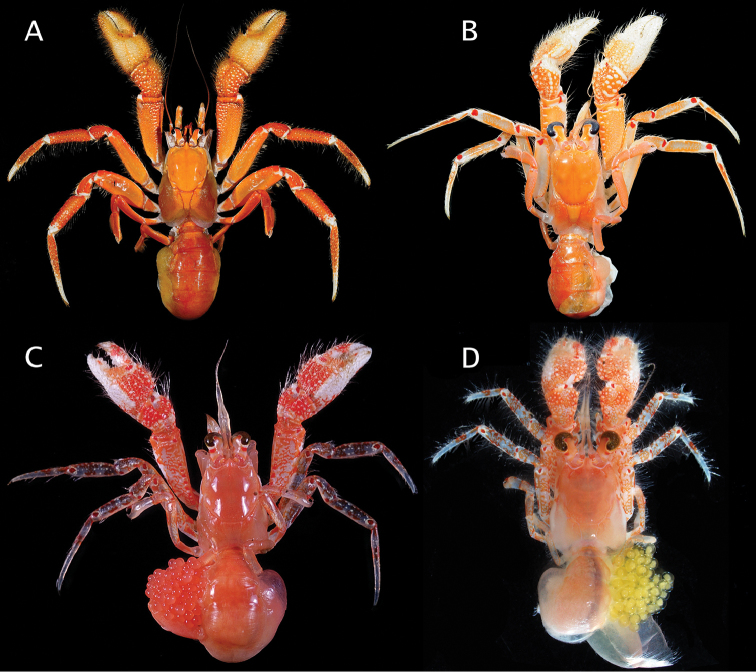
Habitus: **A** *Paguropsis gigas* sp. n., holotype male, 23.0 mm, South China Sea, NANHAI 2014, sta DW 4105 (NTOU A01445) **B** *Paguropsis lacinia* sp. n., paratype female, 9.8 mm, Papua New Guinea, PNG, sta CP 4254 (MNHN-IU-2013-2288) **C** *Paguropsina pistillata* gen et sp. n., ovig female, 4.2 mm, Philippines, PANGLAO, sta T37 [#08] (LKCNHM ZRC) (photograph: T-Y Chan) **D** *Paguropsina inermis* gen et sp. n., female, 2.5 mm, Japan, Ogasawara Islands, (CBM-ZC 14206) (photograph: T Komai).

Figs 15 , 16 , 17 , 18A , Table 1

##### Type material.

Holotype: male 23.0 mm, South China Sea, NANHAI 2014, cruise OR 5, sta DW 4105, 13°57.8902'N, 115°25.5073'E, 297–565 m, 3 Jan 2014 (NTOU A01445).

##### Paratype.

1 ovig female 20.5 mm, same sta data as holotype (NTOU A01446).

##### Description.

*Shield* (Figs 15A, 18A) subtriangular, ca. 1.3 times as long as broad; dorsal surface glabrous except for setae anterolaterally and transverse fringe of short setae on sloping anterior margins of gastric region; anterior margin between rostrum and lateral projections concave; lateral projections broadly triangular, each terminating in small spine; posterior margin roundly truncate; lateroventral distal angle produced into strong blunt spine adjacent to proximal margin of first antennal segment. Rostrum (Fig. 15A) acutely triangular, arched dorsally, strongly produced and extending to distal margin of ocular acicles; with distinct rounded dorsal longitudinal ridge having few short setae laterally, and ending in blunt subterminal spine. Branchiostegite unarmed except for 1 spine on dorsodistal angle of anterodorsal plate, and setose distal margin.

*Ocular peduncles* ca. 0.4 length of shield, constricted medially, glabrous except for dorsal longitudinal row of short setae; corneas strongly dilated, diameter 0.5 total peduncular length (including the cornea). Ocular acicles small, triangular, each terminating in blunt, setose distal spine directed anteriorly.

*Antennular peduncles* when fully extended overreaching distal margins of corneas by nearly full length of ultimate peduncular segment; ultimate and penultimate segments glabrous or at most with scattered short setae; basal segment with ventromesial tuft of setae distally; lateral face with distal subrectangular lobe, and setose lobe proximally.

*Antennal peduncles* overreaching distal corneal margins by ca. 0.2 length of ultimate segments. Fifth and fourth segments unarmed, nearly glabrous except for scattered short setae. Third segment with setose spine at ventrodistal angle. Second segment with dorsolateral distal angle produced, terminating in small simple or bifid spine; mesial margin rounded, setose, and small spine on dorsomesial angle. First segment unarmed except for moderately long setae on lateral face. Antennal acicle almost reaching distal margin of cornea, slender, nearly straight and terminating in sharp spine, with long setae dorsomesially and distally. Antennal flagellum reaching to midpoint of chelae, with few short setae less than one article in length.

*Mouthparts* not markedly different from those described for *Paguropsis typica* (see Fig. 4A–F). Maxilliped 3 with exopod 4.0 times as long as broad.

*Chelipeds* (Figs 15B, 18A) subequal, similar in armature and setation; dorsal surfaces of chelae and carpi covered with dense tufts or short rows of long, bristle-like setae nearly obscuring armature below; ventral surfaces of palms with well-spaced tufts of long bristle-like setae, otherwise smooth except for few low tubercles or blunt spines ventrolaterally and ventromesially. Fingers with narrow hiatus proximally, forming spoon-like shape in ventral view when closed; each finger terminating in small curved corneous claw and subdistal blunt calcareous tooth ventral to claw, both claws and teeth interlocking when fingers closed; cutting edge of dactyl with terminal row of small, fused corneous teeth on distal one-third, and row of blunt calcareous teeth on proximal two-thirds and decreasing in size distally; cutting edge of fixed finger with row of blunt calcareous teeth decreasing in size distally. Dactyl 1.4–1.7 times as long as palm; dorsal surface convex, with numerous tufts of long bristle-like setae, and few small blunt spines or tubercles on rounded mesial surface; ventral surface with less dense tufts of bristle-like setae, lacking spines. Fixed finger with dorsal, lateral, and ventral surfaces similar to dactyl in armature. Palm ca. 0.7 times as long as carpus, dorsal surface convex, covered with numerous small blunt to sharp spines arranged in more or less longitudinal rows each with tufts of long setae; dorsomesial margin with 2–4 irregular rows of spines each with tufts of long setae; dorsolateral margin rounded, not delimited, with irregular rows of small tubercles or spines each with long setae. Carpus 0.5–0.6 times length of merus; dorsal and dorsolateral surfaces with well-spaced spines or short transverse rows of 2 or 3 small spines each bearing tufts of long setae, with longitudinal smooth area medially; dorsolateral margin rounded; dorsomesial margin with irregular rows of spines each bearing tufts of long setae; mesial surface with short transverse rows of bristle-like setae on dorsal half, otherwise smooth; ventral surface smooth, with fringe of long setae on ventrodistal margin. Merus subtriangular in cross-section, nearly as long as chela; dorsal margin with row of low protuberances each bearing transverse row of 2 or 3 small tubercles and bearing tuft of long setae; ventromesial and ventrolateral margins each with irregular row of spines with tufts of long setae; lateral and mesial surfaces with tufts of long and short setae; ventral margin smooth except for moderately dense bristle-like setae. Ischium with row of small spines on ventrolateral margin. Basis with ventromesial row of long setae. Coxa with short, weakly marked longitudinal fissure (Fig. 15C) near distal margin.

*Pereopods 2 and 3* (Fig. 16A–D) similar in armature and setation, distinctly dissimilar in length, with pereopod 2 shorter than pereopod 3 (particularly dactyls). Dactyls ca. 1.4 (pereopod 2) or 1.8 (pereopod 3) times as long as propodi; nearly straight except for slightly incurved distal portion, terminating in sharp corneous claw; lateral and mesial faces with shallow longitudinal concavity; all surfaces covered with tufts of bristle-like setae often arranged in oblique rows; ventromesial margin with distal row of minute corneous spinules (pereopod 2) or lacking armature (pereopod 3) except for setae. Propodi 1.4 (pereopod 2) or 1.2 (pereopod 3) times as long as carpi; dorsolateral and ventrolateral surfaces with tufts or rows of tufts of long bristle-like setae; mesial face with scattered setae. Carpi unarmed except for scattered setae laterally and mesially, tufts of short setae dorsally, and distolateral fringe of long setae. Meri with fringe of long setae on ventral margins; ventral margin of merus of pereopod 2 with row of small blunt spines hidden by setae. Ischia unarmed except scattered setae on lateral face of pereopod 2. Coxae with ventromesial margin sparsely setose; coxae of pereopods 3 (Fig. 15C) narrowly separated by 0.2 ventral length of 1 coxa. Sternite XI (Fig. 15C) having anterior lobe flat or weakly concave; posterior lobes each with transverse fringe of setae.

*Pereopod 4* (Figs 15D, 17A) with chela as long as or slightly shorter than carpus, ca. 2.2 times as long as high. Dactyl and fixed finger leaving wide gap when closed, each terminating in sharp, inwardly curved corneous claws crossing when closed. Dactyl strongly curved, dorsal margin with row of short setae; cutting edge with ventrolateral distal row of 4 or 5 small corneous-tipped spines (in addition to corneous claw). Fixed finger curving inward, cutting edge with 4 strong corneous-tipped spines (in addition to corneous claw) arranged like bear claw; lateral face usually with 1–4 minute scale-like corneous spines near base of finger. Palm straight, broad, ca. 1.6 times as long as high, lateral face weakly concave medially; dorsal face with long simple setae in addition to prominent dense patch of thin capsulate setae arranged in oblique fringes and occupying oval area from dorsal margin to midlength of lateral face; ventral margin with sparse tufts of short setae continuing on fixed finger. Carpus unarmed except for long setae on dorsal margin, and short oblique fringes of thin capsulate setae on dorsodistal angle of lateral face, and scattered short setae ventrally. Merus 0.5 or 0.6 times as long as meri of pereopod 2 and pereopod 3, respectively. Sternite XII with fringe of long dense setae (Fig. 15C).

*Pereopod 5* (Fig. 17B) with chela as long as carpus and 0.7 times as long as merus, with long, brush-like setae on dorsal and ventral surfaces; merus and carpus each with dorsal and ventral row of long setae. Dactyl with rasp on ventral face. Propodal rasp consisting of minute, ovate scales, occupying 0.2 length of propodus. Ischium with setae dorsally and ventrally. Coxa with fringe of long bristle-like setae on rounded ventromesial distal angle.

*Male* gonopod 1 with inferior lamella armed on distal margin with posterior row of slender, semitransparent hook-like spines, and 2–4 anterior irregular rows of small, straight or slightly curved corneous spines. Gonopod 2 with distal segment strongly twisted distally, densely setose. In only known male, left side with biramous, reduced pleopods 3 and 4, and uniramous vestigial pleopod 5; right side with uniramous vestigial pleopod 3 and lacking pleopods 4 and 5.

*Female* (only one specimen known) with unpaired left pleopods 2–4 well developed, lacking pleopod 5. Brood pouch large, subquadrate, distal margin strongly scalloped and fringed with setae.

*Uropodal exopods* (Fig. 17C) slender, broadly curved, terminating in strong spine, anterior margin with fringe of long setae and row of well-spaced corneous-tipped spines; endopods short, strongly curved, anterior margin with long setae and 1 or 2 irregular rows of corneous-tipped spines; protopods with strong, ventrally curved proximal spine.

*Telson* (Fig. 17C) slightly subrectangular, broader than long; posterior lobes obscurely divided medially, terminal margins unarmed except for fringe of long setae.

##### Genetic data.

See Table 1.

##### Color

(Fig. 18A). Shield light orange except for white anterior margins. Ocular peduncles orange dorsally except for white near cornea and proximally, otherwise white; corneas black; ocular acicles light orange except for white distal spine and margins. Antennules orange, flagella of similar but darker color than peduncle. Antennal peduncles with fifth segment light orange on dorsal and lateral surfaces, otherwise white; acicle with tinge of light orange distally; flagella light orange. Chelipeds with yellow bristle-like setae; chelae very light orange to cream; meri and carpi orange except for white spines and tubercles, and white portion of ventrolateral and ventromesial distal margins of meri. Pereopods 2 and 3 generally orange except for white on proximal margins of ischia, distal margins of meri, carpi and propodi, and distal 0.3 of dactyls; meri each also with small white patch proximally on lateral faces. Pereopods 4 and 5 orange.

##### Etymology.

The specific epithet is from the Latin *gigas*, meaning giant, used as a noun in apposition, and in reference to the large size attained by individuals of this new species.

##### Distribution.

Western Pacific: known so far only from the South China Sea. Depth: 297 to 565 m.

##### Habitat and symbiont.

Found with indeterminate species of acontiate anemone (see “Remarks” under genus).

##### Variations.

With only two specimens known, no variations can be evaluated.

##### Affinities.

*Paguropsis gigas* sp. n. shares with *P. lacinia* sp. n. the presence of a prominent patch of dense, capsulate setae on the dorsolateral face and dorsal margin of the palm of the chelae of pereopod 4. The shape and arrangement of the setae on the patch, however, is quite different in both species. In *P. gigas* sp. n., the capsulate setae are relatively short and arranged in a series of oblique fringes that occupy and oval area from the dorsal margin to midlength of the lateral face of the palm (Figs 15D, 17A). In *P. lacinia* sp. n., the capsulate setae are narrow and long, not arranged in rows, and occupy only one-third of the lateral surface of the palm (Figs 21B, C, 22F). The ultrastructure of these setae has not been studied, but they appear to be hollow and filled with a light brown fluid (at least in preserved specimens). The function, if any, of these setae is unknown, although conceivably they could be used for feeding or grooming. The presence of this unusual patch in these two species might suggest a close phylogenetic relationship. However, in other morphological characters these two species differ substantially, and thus the presence in both of a patch appears to reflect homoplasy. The two species differ also as follows: growth patterns, with specimens of *P. gigas* sp. n. reaching a much larger size than those of *P. lacinia* sp. n.; stronger spination of chelipeds, and denser setation of chelipeds and pereopods 2 and 3 in *P. gigas* sp. n. than in *P. lacinia* sp. n.; dactyls more robust and wider in *P. gigas* sp. n. than in *P. lacinia* sp. n., 7–9 times as long as broad in the former vs. 10–16 times as long as broad in the latter; and once again, as in other species of *Paguropsis*, drastically different coloration patterns (Fig. 18A, B).

##### Remarks.

Among the species discussed in this revision, *Paguropsis gigas* sp. n. and *P. andersoni* are similar in that they grow to the largest size, the former reaching a shield length of 23.0 mm, the latter to a shield length of 20.6 mm. Morphologically they are also generally similar, both having strong, dense spination and bristle-like setation on the chelipeds, and numerous tufts of bristle-like setae on pereopods 2 and 3. The lateral surfaces of the dactyls of pereopods 2 and 3 are concave in both species, although only moderately so and along the proximal half or more of the segment in *P. gigas* sp. n., whereas the concavity is strongly marked along the proximal one-third in *P. andersoni*. Despite these similarities, *P. gigas* sp. n. differs markedly from *P. andersoni*, the former having a prominent dense patch of thin capsulate setae arranged in oblique fringes on the dorsal margin and dorsolateral face of the palm of the chelate pereopod 4, whereas in the latter there is no patch of setae and only a fringe of long setae on the dorsal margin of the palm. Furthermore, in *P. gigas* sp. n. the palm of pereopod 4 is often more noticeably lateromesially flattened than in *P. andersoni*, although there is some variation in this character in both species. The coloration of the ocular peduncles, chelipeds, and pereopods is also clearly different in these two species (compare Figs 8C, 18A).

#### Paguropsis lacinia sp. n.

Taxon classificationAnimaliaDecapodaDiogenidae

http://zoobank.org/C96210D0-F9C8-4739-B50A-81035FF522E3

**Figure 19.**
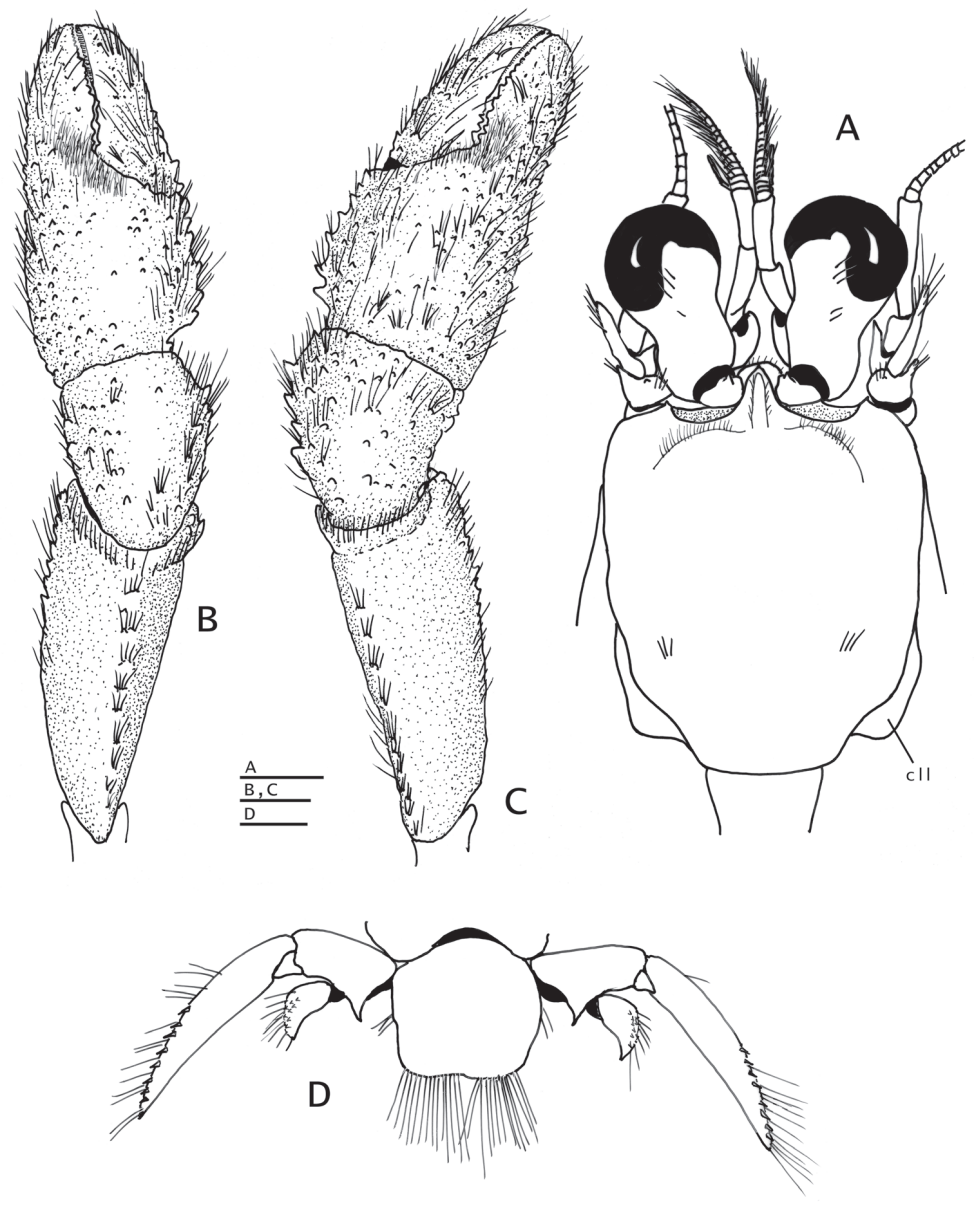
*Paguropsis lacinia* sp. n., holotype female, 8.4 mm, Solomon Islands, SALOMON 2, sta CP 2201 (USNM 1442006): **A** shield and cephalic appendages, dorsal **B** left cheliped, dorsal **C** right cheliped, dorsal **D** uropods and telson, dorsal. Abbreviations: cll, carapace lateral lobe. Scale bars: 2 mm (**A**), 2 mm (**B, C**), 1 mm (**D**).

**Figure 20.**
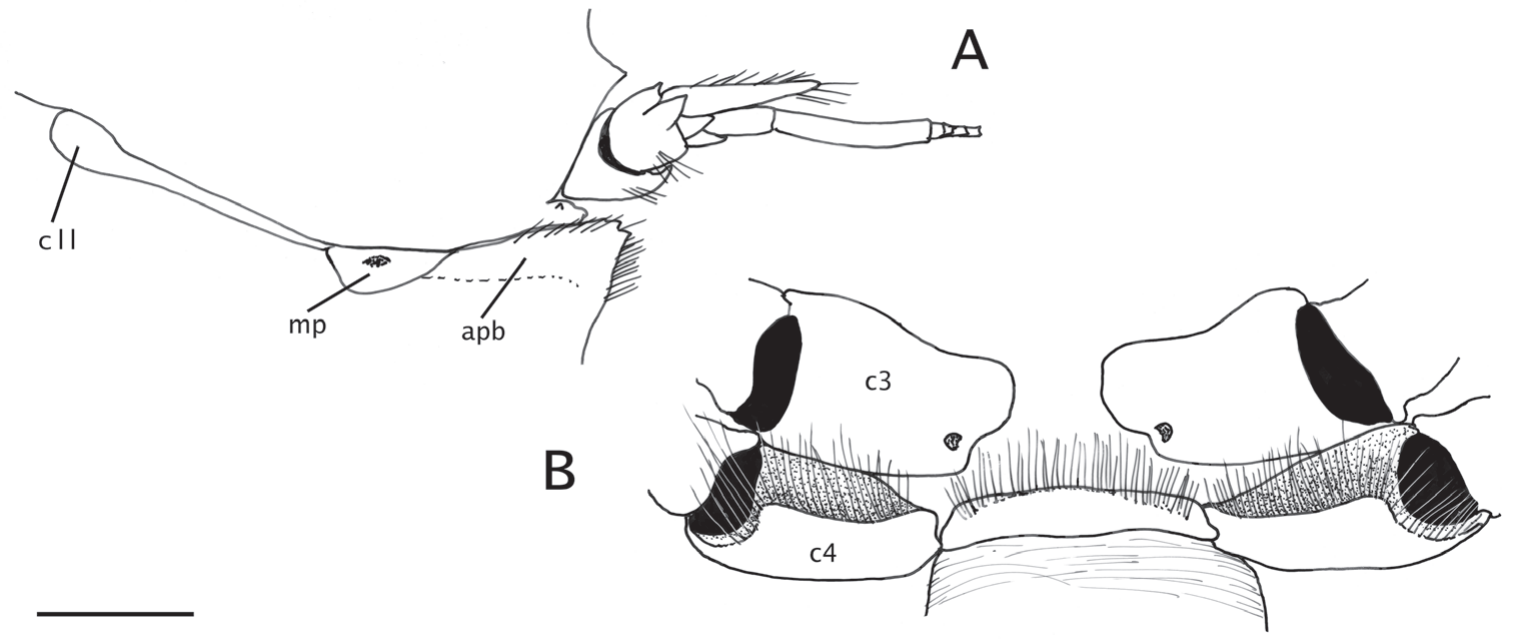
*Paguropsis lacinia* sp. n. holotype female, 8.4 mm, Solomon Islands, SALOMON 2, sta CP 2201 (USNM 1442006). **A** right antennal peduncle, branchiostegite, and anterodorsal portion of posterior carapace, lateral **B** coxae of pereopods 3 and 4 and sternites XI and XII. Abbreviations: apb, anterodorsal plate of branchiostegite; cll, carapace lateral lobe; mp, median plate; C3, C4, coxae of pereopods 3 and 4. Scale bar: 2 mm.

**Figure 21.**
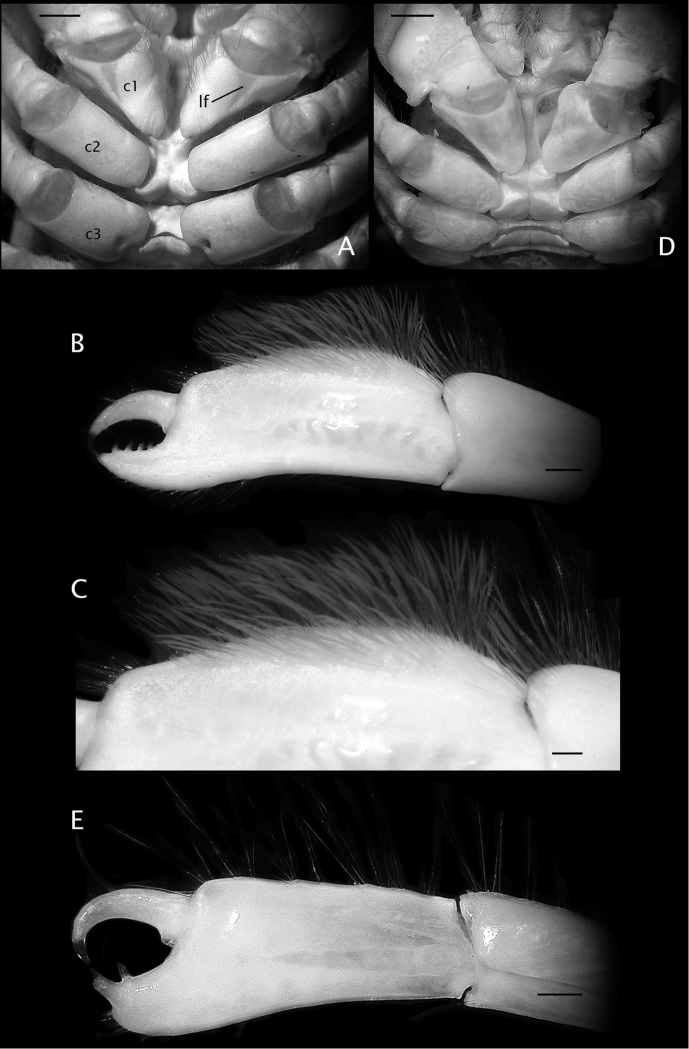
**A–C** *Paguropsis lacinia* sp. n., holotype female, 8.4 mm SALOMON 2, CP 2201 (USNM 1442006) **D, E** *Paguropsina pistillata* gen. et sp. n., holotype male, 4.4 mm New Caledonia, EBISCO, CP 2499 (USNM 1442008). **A, D** coxae of pereopods 1–3, and sternites IX–XI **B, E** chela of pereopod 4 **C** fringe of capsulate setae on dorsal surface of palm of pereopod 4. Abbreviations: C1–3, coxae of pereopods 1–3; lf, longitudinal fissure. Scale bars: 2 mm (**A**), 0.25 mm (**B, D**), 0.5 mm (**C**), 2 mm (**D**).

**Figure 22.**
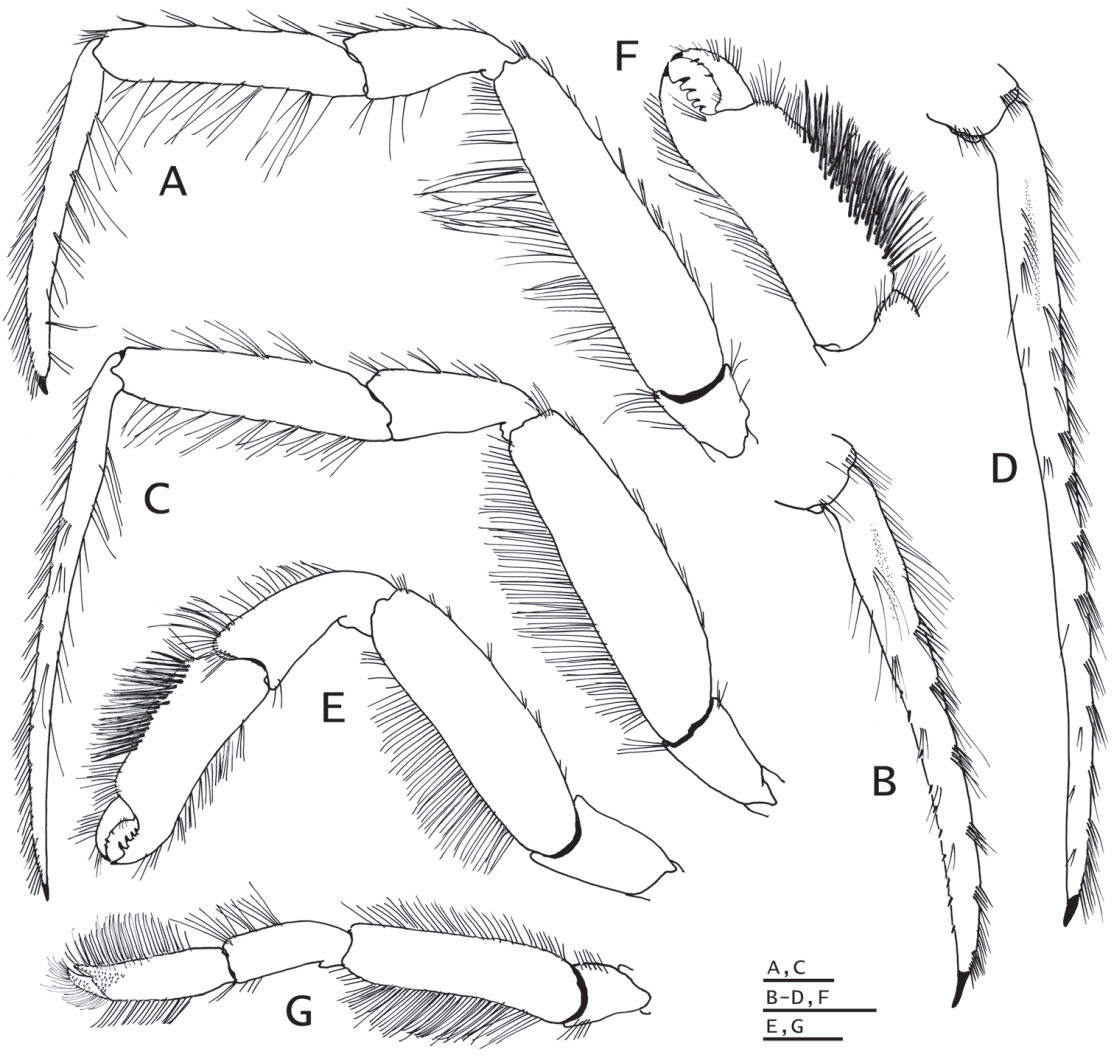
*Paguropsis lacinia* sp. n. holotype female, 8.4 mm, Solomon Islands, SALOMON 2, sta CP 2201 (USNM 1442006). **A** left pereopod 2, lateral **B** dactyl of same, mesial **C** left pereopod 3, lateral **D** dactyl of same, mesial **E** left pereopod 4, lateral **F** chela of same, lateral **G** left pereopod 5, lateral. Scale bars: 2 mm.

Figs 18B , 19 , 20 , 21A–C , 22 , Table 1

##### Type material.

Holotype: female 8.4 mm, Solomon Islands, SALOMON 2, NO *Alis*, NW of Isabel Island, sta CP 2201, 07°43.5'S, 158°29.9'E, 307–310 m, 25 Oct 2004 (USNM 1442006).

##### Paratypes.

*Papua New Guinea*: MADEEP, NO *Alis*: NW of Kavieng, sta CP 4254, 02°28'S, 150°42'E, 273–324 m, 24 Apr 2014: 1 female 9.8 mm, color photograph (Fig. 18B) (MNHN-IU-2013–2288).

*Salomon Islands*: SALOMON 1, NO *Alis*: N Buena Vista Island, sta DW 1765, 08°43'S, 160°07'E, 325–380 m, 27 Sep 2001: 1 female 6.1 mm (MNHN-IU-2014–9370); NW of San Cristobal, sta CP 1831, 10°12'S, 161°19'E, 135–325 m, 5 Oct 2001: 1 ovig female 10.3 mm (MNHN-IU-2014–9367).

*Tonga Islands*: BORDAU 2, NO *Alis*: NW of Tongatapu, sta CP 1643, 21°05'S, 175°22'W, 487 m, 22 Jun 2000: 1 male 7.3 mm, 1 female 7.2 mm (MNHN-IU-2014–9366).

*New Caledonia*: NORFOLK 1, NO *Alis*: Norfolk Ridge, Crypthelia Bank, sta CP 1731, 23°21'S, 168°16'E, 310–788 m, 27 Jun 2001: 1 male 8.5 mm (MNHN-IU-2014–9360). NORFOLK 2, NO *Alis*: Munida Bank, sta DW 2142, 23°01'S, 168°17'E, 550 m, 3 Nov 2003: 2 males 6.7, 6.8 mm (MNHN-IU-2014–9389). EBISCO, NO *Alis*: W of Bellona, sta CP 2551, 21°06'S, 158°35'E, 637–650 m, 11 Oct 2005: 1 male 7.8 mm (USNM 1442007).

##### Description.

*Shield* (Figs 18B, 19A) weakly subtriangular (lateral margins subparallel on anterior two-thirds), 1.2 times as long as broad; dorsal surface glabrous except for tufts of setae anterolaterally and transverse fringe of short setae on sloping anterior margins of gastric region; anterior margin between rostrum and lateral projections concave; lateral projections broadly triangular, each terminating in small spine; posterior margin relatively wide, roundly truncate; lateroventral distal angle with strong spine adjacent to proximal margin of first antennal segment. Rostrum acutely triangular, arched, and curving ventrally, extending nearly to distal margin of ocular acicles, terminating in small spine; with distinct rounded dorsal longitudinal ridge with fringe of short setae laterally, and terminating subdistally in rounded tip. Branchiostegite (Fig. 20A) unarmed except for 1 or 2 minute tubercles on dorsodistal angle of anterodorsal plate, and setose distal margin.

*Ocular peduncles* stout, constricted medially and broadening distally, ca. 0.5 length of shield, glabrous except for row of few short dorsodistal setae; corneas strongly dilated, diameter 0.5–0.6 total peduncular length (including the cornea). Ocular acicles small, triangular, armed with distal or dorsodistal spine often directed anteriorly or anterodorsally.

*Antennular peduncles* when fully extended overreaching distal margins of corneas by nearly full length of ultimate peduncular segments. Ultimate and penultimate segments glabrous or at most with scattered short setae. Basal segment with ventromesial setae distally; lateral face with distal subrectangular lobe with or without small tubercles, small medial spine, and setose lobe proximally.

*Antennal peduncles* reaching or at most slightly exceeding distal margin of corneas. Fifth and fourth segments unarmed except for scattered short setae and laterodistal tuft of long setae. Third segment with strong spine at ventrodistal angle. Second segment with dorsolateral distal angle produced, terminating in small simple spine; mesial margin rounded, setose, and small spine on dorsomesial angle. First segment unarmed. Antennal acicle reaching to proximal margin of cornea, slender, terminating in sharp spine, with long setae distally, at most with 1 or 2 minute proximal tubercles on mesial margin. Antennal flagellum long, reaching to distal end of cheliped fingers, articles with 1 or 2 short setae (< 1 article in length) and usually with 1 or 2 long setae every 12 articles or so.

*Mouthparts* not markedly different from those described for *Paguropsis typica* (see Fig. 4A–F). Maxilliped 3 with exopod ca. 4.2 times as long as broad.

*Chelipeds* (Figs 18B, 19B, C) subequal, similar in armature and setation; dorsal surfaces of chelae and carpi moderately covered with short bristle-like setae not obscuring armature below, setation somewhat denser near base of fixed finger and distal portion of palm; ventral surfaces of chelae and carpi with scant setae or tufts of setae, and scattered small tubercles. Dactyl and fixed finger with narrow hiatus proximally when closed, forming spoon-like shape in ventral view when closed; each terminating in small curved corneous claw and subdistal blunt calcareous tooth ventral to claw, both claws and teeth interlocking when fingers closed; cutting edge of dactyl with terminal row of small, fused corneous teeth on distal one-third, and row of unequal calcareous teeth on proximal two-thirds; cutting edge of fixed finger with row of blunt calcareous teeth decreasing in size distally. Dactyl 1.4 times as long as palm; dorsal surface convex, armed with small spines or tubercles; dorsomesial margin rounded, armed with small spines or tubercles; ventral face with well-spaced tufts of long bristle-like setae, lacking spines. Fixed finger with dorsal, lateral and ventral surfaces similar to dactyl in armature. Palm slightly shorter than carpus, dorsal surface with scattered small tubercles medially, dorsolateral margin rounded, not delimited, with irregular rows of small spines or tubercles, dorsomesial margin with row of strong spines. Carpus ca. 0.6 times length of merus; dorsal and dorsolateral surfaces with well-spaced small spines or tubercles; dorsomesial margin with row of strong spines or tubercles, and small dorsodistal spine; dorsolateral margin rounded, not delimited; mesial surface smooth, unarmed except for setae on distal margin; ventral surface unarmed, ventrodistal margin with fringe of sparse long setae. Merus nearly as long as chela, subtriangular in cross-section; dorsal margin with row of protuberances accompanied by tufts of short setae, ventromesial and ventrolateral margins each with irregular row of spines with setae; lateral and mesial surfaces unarmed except for scattered short setae. Ischium with row of small spines on ventrolateral margin. Basis with ventromesial row of setae. Coxa with well-marked longitudinal fissure (Fig. 21A) on ventral surface.

*Pereopods 2 and 3* (Figs 18B, 22A–D) similar in armature and setation, distinctly dissimilar in length, with pereopod 2 shorter than 3. Dactyls ca. 1.7 (pereopod 2) or 2.4 (pereopod 3) times as long as propodi; dactyl of pereopod 2 broadly curved, terminating in sharp corneous claw, with ventromesial distal row of usually ten minute short spinules; dactyl of pereopod 3 slender, nearly straight in lateral view, 1.5–1.6 times as long as dactyl of pereopod 2, ventromesial margin unarmed; dorsal and ventral margins with tufts of moderately long setae. Propodi 1.3–1.4 times as long as carpi; dorsal and ventral surfaces with tufts of long setae. Carpi unarmed except for tufts of setae dorsally, dorsodistal angle blunt or with obscure small tubercle. Meri unarmed except for fringe of long setae ventrally. Ischia unarmed except for scattered short setae. Coxae with ventromesial row of setae. Coxae of pereopods 3 (Figs 20B, 21A) separated by ca. 0.3 ventral length of 1 coxa. Anterior lobe of sternite XI (between pereopods 3; Fig. 21A) flat or slightly concave, unarmed, posterior lobes weakly arched, sloping, each with transverse fringe of sparse setae.

*Pereopod 4* with chela (Fig. 21B) ca. 1.2 times as long as carpus and 1.7 times as long as high, palm 1.8–1.9 as long as high. Dactyl and fixed finger leaving wide gap when closed, each terminating in sharp, inwardly curved corneous claw crossing at tips when closed. Dactyl strongly curved inward, dorsal margin sparsely setose; cutting edge with ventrolateral row of usually 3 small corneous-tipped spines (in addition to corneous claw). Fixed finger curving inward, cutting edge with 4 strong corneous-tipped spines (in addition to corneous claw) arranged like bear claw; lateral face usually with 1 or 2 minute scale-like corneous spines near base of finger. Palm straight or slightly curved, dorsal face with prominent patch (Fig. 21B, C) of dense long, narrow capsulate setae extending for one-third of lateral surface. Carpus unarmed except for fringe of long setae dorsally and scattered setae ventrally. Merus ca. 0.7 times as long as meri of pereopods 2 and 3. Sternite XII (Fig. 20B) narrow, undivided or obscurely divided medially, with transverse fringe of long setae.

*Pereopod 5* (Fig. 22G) with chela as long or slightly longer than carpus, and 0.7 times as long as merus. Chela with long, brush-like setae on dorsomesial and ventromesial face, Carpus with sparse tufts of setae on dorsal and ventral margins. Dactyl with rasp on ventral face. Propodal rasp weakly developed and consisting of minute, ovate scales, occupying 0.3 length of propodus. Ischium with setae dorsally and ventrally. Coxa with ventrodistal setae.

*Male* gonopod 1 with inferior lamella armed on distal margin with posterior row of slender, semitransparent hook-like spines, and 1 or 2 irregular rows of small straight or slightly curved corneous spines. Gonopod 2 with distal segment strongly twisted distally, densely setose. Pleon with left unpaired, reduced, biramous pleopods 3 and 4, lacking pleopod 5.

*Female* with left side having unpaired pleopods 2–4, and reduced biramous pleopod 5 (no unpaired pleopods 2–5 on right side). Brood pouch large, subquadrate, distal margin scalloped and fringed with setae.

*Uropodal exopods* (Fig. 19D) slender, nearly straight or broadly rounded, terminating in strong spine, anterior margin with fringe of long well-spaced setae and row of well-spaced corneous-tipped spines; endopods short, strongly curved, anterior margin with long setae and 1 or 2 irregular rows of corneous-tipped spines; protopods with strong, curved proximal spine.

*Telson* (Fig. 19D) subquadrate; posterior lobes separated by obsolete median cleft, terminal margins unarmed except for fringe of long setae.

##### Genetic data.

See Table 1.

##### Color

(Fig. 18B). Shield and calcified portion of posteromedian carapace evenly yellowish orange. Ocular peduncles yellowish orange except for white portions adjacent to corneas; corneas black; ocular acicles yellowish orange. Antennules and antennae light orange or pink, flagella of similar but darker color than peduncle but lighter. Chelipeds generally yellowish orange with white spines or tubercles; chelae proximally of lighter tone than rest of chelipeds, fading to nearly white or cream on dactyls and fixed fingers; carpi of darkest orange tone, ventromesial angle with reddish portion; meri light orange, with very short reddish stripe on distolateral and distomesial margin. Pereopods 2 and 3 with meri, carpi and propodi each with red spot distally on lateral face; dactyl light orange fading to white distally, and white dorsal margin; propodi and carpi light orange except for white distally around red spot; meri with light orange dorsal and ventral margins, otherwise white to cream except for red spot. Pereopod 4 more or less evenly orange except for white dactyl and fixed finger of chelae.

##### Etymology.

The species name is from the Latin *lacinia*, a fringe, and refers to the characteristic setae of this new species on the dorsal margin of the palm of the chelate pereopod 4.

##### Distribution.

Western Pacific: from off northern Papua New Guinea, Solomon Islands, Tonga Islands, and New Caledonia. Depth: 135 to 788 m.

##### Habitat and symbiont.

Found with indeterminate species of acontiate anemone (see “Remarks” under genus).

##### Variations.

No appreciable morphological variations were observed other than those incorporated in the description.

##### Affinities.

See “Affinities” under *Paguropsis gigas* sp. n.

##### Remarks.

The most prominent, distinctive morphological character of this species is the presence of a dense patch of capsulate, simple setae on the dorsal margin of the palms of pereopods 4 (Figs 21B, C, 22F), although a patch of capsulate setae is also present on the palm of pereopod 4 in *Paguropsis gigas* sp. n.. As noted under *P. gigas* sp. n., the extent and arrangement of the setae is different in these two species. The function, if any, of the patch is unknown. Furthermore, the unique coloration of *P. lacinia* sp. n., with pereopods 2 and 3 having meri, carpi and propodi each with a red spot distally on the lateral face (Fig. 18B), clearly distinguishes this species from other congeners.

#### Genus Paguropsina gen. n.

Taxon classificationAnimaliaDecapodaDiogenidae

http://zoobank.org/B40AEE97-2A4C-4EF5-9F04-47C5757FAF66

##### Diagnosis.

**Figure 23.**
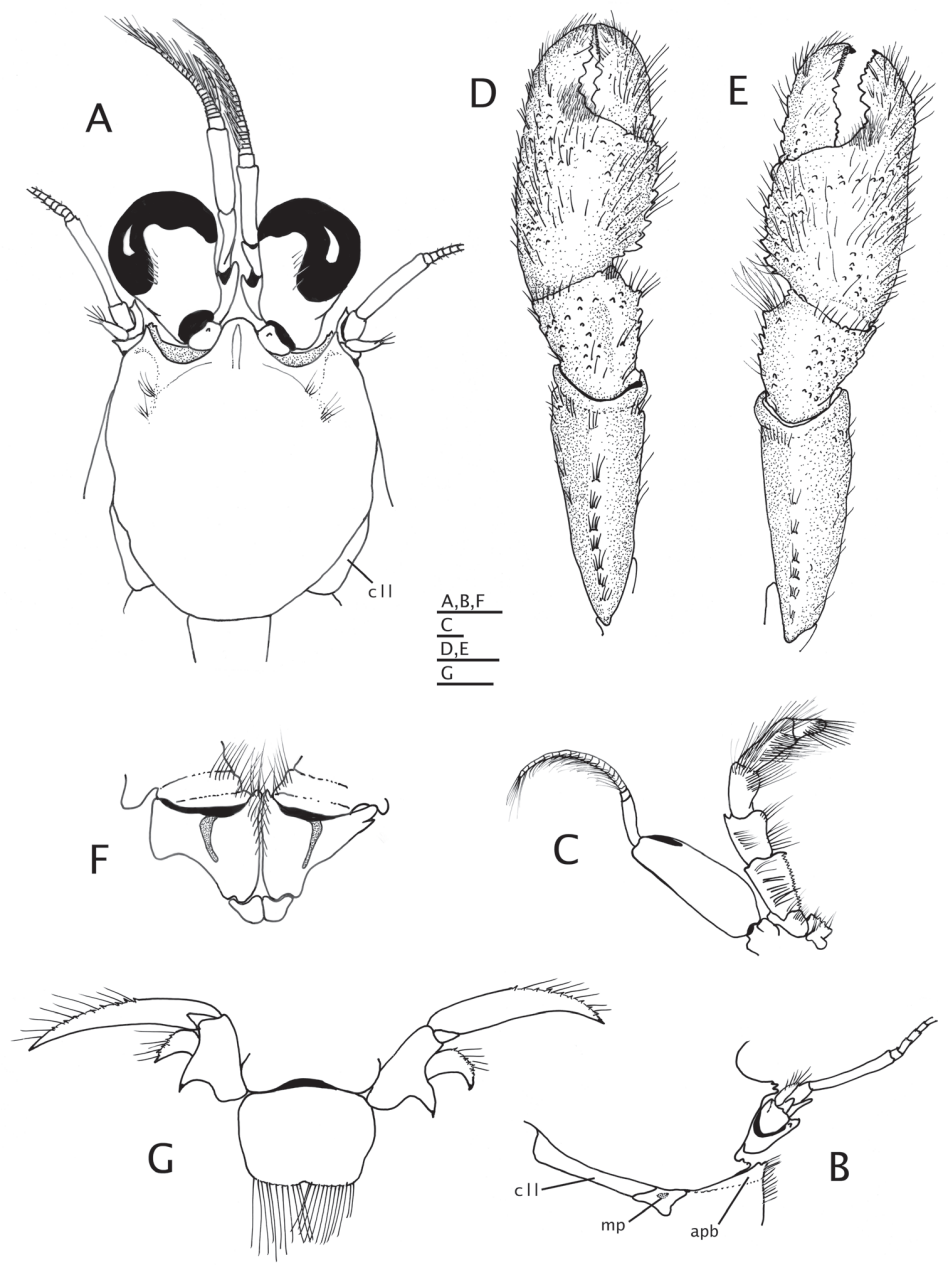
*Paguropsina pistillata* gen. et sp. n., holotype male, 4.4 mm New Caledonia, EBISCO, CP 2499 (USNM 1442008). **A** shield and cephalic appendages, dorsal **B** right antennal peduncle, branchiostegite, and anterodorsal portion of posterior carapace, lateral **C** left maxilliped 3, internal **D** left cheliped, dorsal **E** right cheliped, dorsal **F** coxae of chelipeds, ventral **G** uropods and telson, dorsal. Abbreviations: apb, anterodorsal plate of branchiostegite; cll, carapace lateral lobe; mp, median plate. Scale bars: 1 mm (**A, B**), 2 mm (**D, E, G**), 0.5 mm (**C, F**).

Thirteen pairs of quadriserial gills [no pleurobranchs on thoracomere VIII (last)], gills consisting of series of twin lamellae each deeply divided distally into finger-like extensions (e.g., Fig. 25E). Shield well calcified, subovate; dorsal surface somewhat vaulted. Rostrum prominent, subtriangular, arched, and dorsally ridged. Lateral projections of shield each terminating in short vertical keel-like ridge usually armed with few small spines. Branchiostegite with dorsal margin (e.g., Fig. 23B) divided into two calcified plates: one anterodorsal plate poorly delimited ventrally, followed by small, subtriangular plate with distinct central pit. Posterior carapace with well calcified posteromedian plate, and well calcified lateral lobe on each side adjacent to shield. Ocular peduncles stout, half length of shield; corneas strongly dilated (diameter typically ca. 0.8 times length of ocular peduncle, including cornea); ocular acicles relatively small, subtriangular, armed with small or often minuscule dorsodistal spine. Antennal peduncles slender, delicate, not exceeding distal margins of corneas; acicles short, not reaching level of cornea. Mouthparts: maxillule with well-developed and strongly recurved external lobe of endopod; maxilliped 1 with exopodal flagellum, endopod medially bent at nearly right angle, with distinctly developed epipod; maxilliped 3 ischium with well-developed crista dentata, lacking accessory tooth, exopod broad, ca. 2.4 times as long as broad. Epistome unarmed. Chelipeds symmetrical or nearly so, subequal in size, armed with scarce to moderately dense setation and numerous well-spaced small spines or tubercles; coxae each with ventral surface having uncalcified median longitudinal fissure starting on distal margin and incompletely covering length of ventral surface. Pereopods 2 and 3 long and slender; dactyl of pereopod 3 distinctly longer than dactyl of pereopod 2. Sternite XI (between pereopods 3; e.g., Fig. 21D) wide, separating coxae of pereopods 3 by length of 1 coxa; anterior lobe flat, consisting of narrow rod-like plate (typically 8–10 times as broad as long), posterior lobes weakly divided medially by shallow groove into two subrectangular (wider than long), glabrous lobes. Pereopod 4 chelate, extending to subdorsal position to manipulate carcinoecium, lacking rasp-like surfaces; dactyl with cutting edge unarmed; fixed finger with cutting edge unarmed or with 1 corneous spinules; coxae (e.g., Fig. 27B) with anteroventral margin sharply delimited, keel-like. Sternite XII (between pereopods 4; e.g., Fig. 21D, 27B) broad, ridge-like, weakly divided medially, with fringe of setae. Pereopod 5 chelate, with weakly developed propodal rasp. Pleon curling under but not dextrally or sinistrally twisted; pleonal somite 1 not fused to last thoracic somite, with partly calcified tergite and pleura. Male with well-developed paired gonopods 1 and 2, and reduced (uniramous or biramous) pleopod 3–5 on left side only, when present. Female with paired gonopores; with paired uniramous pleopods 1 modified as gonopods; left side with unpaired, well developed, biramous pleopods 2–4 (ovigerous), rarely with vestigial pleopod 5; right side with no unpaired pleopods 2–5; brood pouch large, covering pleopods 2–4 and entire egg mass. Uropods and telson symmetrical; exopods long, slender; endopod small, curved. Telson subrectangular, lacking or with obscure lateral indentations; posterior margin weakly divided into nearly straight or very broadly rounded lobes.

##### Type species.

*Paguropsina pistillata* gen. et sp. n. Gender: feminine.

##### Species included.

In addition to the type species, the genus includes *P. inermis* gen. et sp. n.

##### Etymology.

The generic name is derived from the genus name *Paguropsis*, and using the Latin feminine suffix –*ina*, in reference to the relatively small size of individuals of the two species of this new genus.

##### Distribution.

Subtropical to tropical western Pacific. Depth: 52 to 849 m.

##### Remarks.

The two new species included in this genus have relatively small specimens ranging in shield length from 1.8 to 6.1 mm, with subovate shields, and a general delicate morphology with slender antennal peduncles and pereopods 2 and 3, weakly armed chelipeds having short, simple setation not obscuring the surface features of the segments, stout ocular peduncles, and wide corneas. Aside from the general, subtle appearance, species of *Paguropsina* gen. n. can be separated from those of *Paguropsis* primarily by four characters that are drastically different from those in species of *Paguropsis*. These are: the shape of the gills (lamellae deeply divided distally into finger-like extensions vs. distally divided into filamentous or stub-like extensions in *Paguropsis*); the shape of the exopod of the maxilliped 3 (broad, 2.4 times as long as broad vs. slender, 4 or more times as long as broad in *Paguropsis*); the width of sternite XI (ca. length of one coxa of pereopods 3 vs. less than half length of one coxa in *Paguropsis*); and armature of the cutting edges of dactyl and fixed finger of chela of pereopod 4 (cutting edges of dactyl and fixed finger unarmed or with one distinct corneous spinule vs. cutting edge of dactyl with row of small corneous spines and cutting edge of fixed finger with sharp spines arranged like bear claw in *Paguropsis*). In several other respects species of *Paguropsina* gen. n. and *Paguropsis* also differ albeit the differences are more subtle. The lateral projections of the shield each terminate in a short vertical keel-like ridge in species of *Paguropsina* gen. n., whereas the lateral projections terminate in a small spines without a keel in *Paguropsis* species. Additionally, it appears based on the material examined of both new species herein described under this new genus, that the location of pleopods tends to be fixed on the left side [see “Variations” under each species), whereas in species of *Paguropsis* the pleopods can frequently be present on either side.

#### Paguropsina pistillata

Taxon classificationAnimaliaDecapodaDiogenidae

gen. et sp. n.

http://zoobank.org/DB4BE2DF-B0DF-489C-A3E4-E763465D34A6

**Figure 24.**
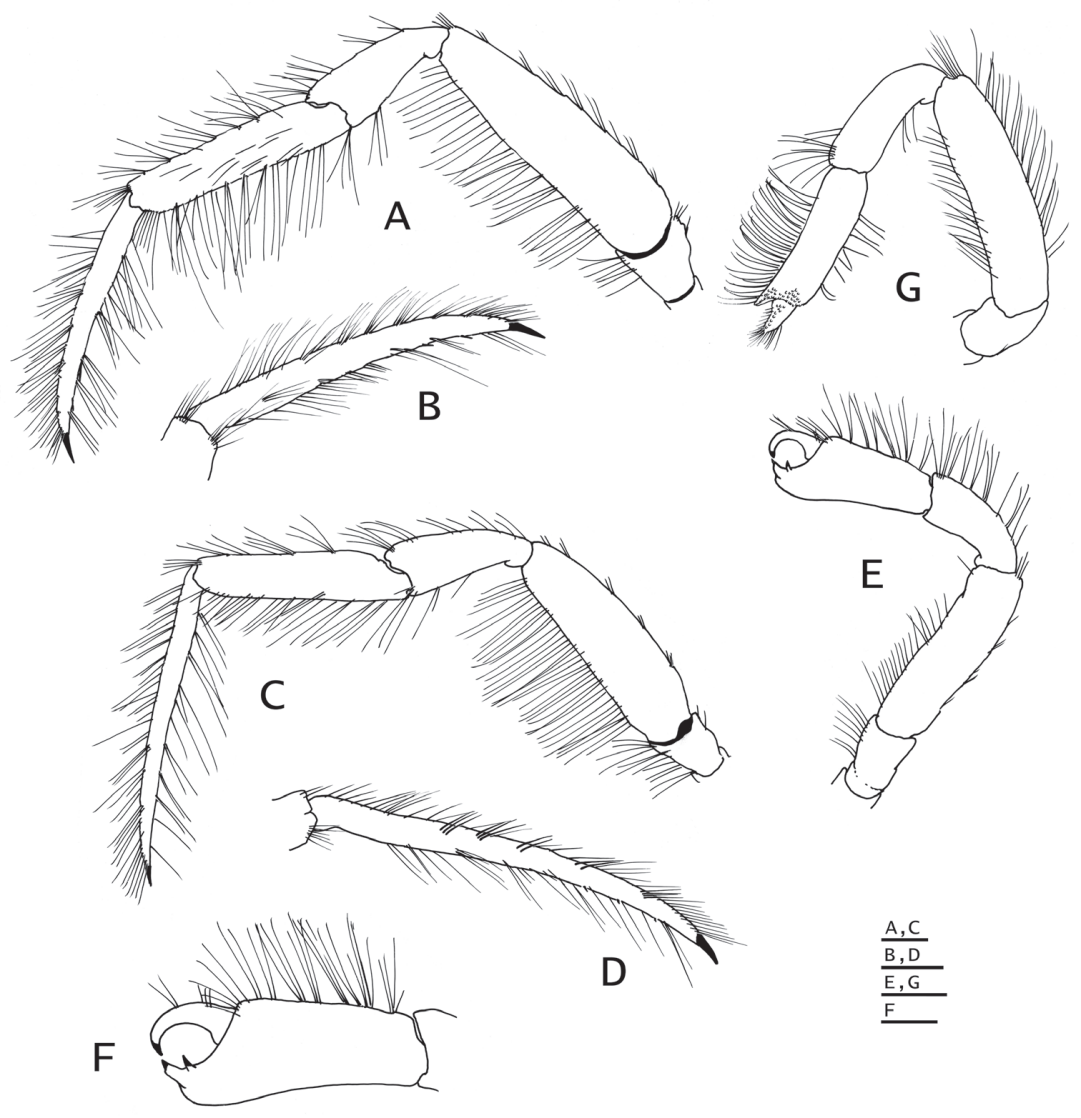
*Paguropsina pistillata* gen. et sp. n., holotype male, 4.4 mm New Caledonia, EBISCO, CP 2499 (USNM 1442008). **A** left pereopod 2, lateral **B** dactyl of same, mesial **C** left pereopod 3, lateral **D** dactyl of same, mesial **E** left pereopod 4, lateral **F** chela of same, lateral **G** left pereopod 5, lateral. Scale bars: 1 mm (**A–E, G**), 0.5 mm (**F**).

Figs 18C , 21D, E , 23 , 24 , Table 1

##### Type material.

Holotype: 1 male 4.4 mm, New Caledonia, EBISCO, NO *Alis*, sta CP 2499, Capel Bank, 24°53'0"S, 159°52'0"E, 286–529 m, 7 Oct 2005 (USNM 1442008).

##### Paratypes.

*Philippines*: PANGLAO 2004, W Pamilacan I. Cervera shoal, sta T37, 09°28'N, 123°51'E, 134–190 m, 4 Jul 2004: ovig female 4.2 mm, color photograph (Fig. 18C) (LKCNHM ZRC).

*Indonesia*: Danish Kei Islands Expedition: sta 49, 05°37'10"S, 132°24'E, 245 m, 3 May 1922: 1 ovig female 4.4 mm (ZMUC-CRU–007046).

*Salomon Islands*: SALOMON 1, NO *Alis*: N Malaita, sta DW 1778, 08°19'S, 160°34'E, 157–253 m, 29 Sep 2001: 1 male 2.7 mm (MNHN-IU-2014–9368); NW San Cristobal, sta CP 1831, 10°12'S, 161°19'E, 135–325 m, 5 Oct 2001: 1 male 4.0 mm, 1 ovig female 3.1 mm (ex MNHN-IU-2013–5582, USNM 1441980); E of Guadalcanal, sta CP 1857, 09°40'S, 160°49'E, 720–849 m, 7 Oct 2001: 1 female 3.4 mm (MNHN-IU-2014–9369).

*New Caledonia*: MUSORSTOM 6, NO *Alis*: Loyalty Islands, [sta number lost]: 1 male 3.1 mm (USNM 1441979). LIFOU, NO *Alis*: Lifou, Santal Bay, SE of Récif Shelter, sta DW 1648, 20°54'S, 167°03'E, 150–200 m, 7/19 Nov 2000: 2 ovig females 3.1, 3.4 mm (MNHN-IU-20149375). NORFOLK 1, NO *Alis*: Norfolk Ridge, Brachiopode Bank, sta DW 1657, 23°26'S, 167°50'E, 305–332 m, 19 Jun 2001: 1 female 3.7 mm (MNHN-IU-2014–9363); Norfolk Ridge, Kaimon-Maru Bank, sta DW 1679, 24°45'S, 168°10'E, 298–324 m, 22 Jun 2001: 1 female 4.0 mm (MNHN-IU-2014–9354). EBISCO, NO *Alis*: Capel Bank, sta CP 2492, 24°44'S, 159°41'E, 285 m, 6 Oct 2005: 74 males 1.8–2.3 mm, 65 females 1.9–3.4 mm, 23 ovig females 3.0–4.0 mm (MNHN-IU-2014–9401); Capel Bank, sta CP 2493, 24°44'0"S, 159°43'0"E, 285–545 m, 6 Oct 2005: 1 male 4.1 mm (USNM 1442021), 56 males 2.5–4.3 mm, 15 females 1.9–3.7 mm, 48 ovig females 1.9–3.4 mm (MNHN-IU-2014–9402); Capel Bank, sta CP 2499, 24°53'S, 159°52'E, 286–529 m, 07 Oct 2005: 2 males 2.4, 3.3 mm (MNHN-IU-2013–5659); Capel Bank, sta CP 2505, 24°45'S, 159°43'E, 328–463 m, 7 Oct 2005: 1 male 4.0 mm, 1 ovig female 3.6 mm (MNHN-IU-2014–9391); Capel Bank, sta CP 2507, 24°43'0"S, 159°43'0"E, 286 m, 7 Oct 2005: 1 male 4. 0 mm (USNM 1442011); Capel Bank, sta DW 2508, 24°41'0"S, 159°43'0"E, 304–350 m, 7 Oct 2005: 1 male, 3.2 mm (USNM 1442012); Kelso Bank, sta DW 2513 24°6'0"S, 159°42'0"E, 280–500 m, 8 Oct 2005: 1 female 3.0 mm (USNM 1442025); Kelso Bank, sta CP 2519, 24°8'0"S, 159°42'0"E, 310–463 m, 8 Oct 2005: 2 males 3.0, 3.1 mm (USNM 1442023); Nova Sud Bank, sta CP 2524, 24°6'0"S, 159°42'0"E, 315–325 m, 9 Oct 2005: 1 ovig female 3.0 mm (USNM 1441985); N of Nova Bank, sta DW 2538, 22°20'S, 159°25'E, 318–323 m, 10 Oct 2005: 1 ovig female 3.3 mm (MNHN-IU-2014–9392); Chesterfield Plateau, sta CP 2591, 19°4'0"S, 158°28'0"E, 244–258 m. 17 Oct 2005: 1 female ovig 3.7 mm (USNM 1442010); Chesterfield Plateau, sta CP 2592, 19°42'0"S, 158°30'0"E, 273–281 m, 17 Oct 2005: 1 male 4.1 mm (USNM 1442024); Chesterfield Plateau, sta CP 2593, 19°43'0"S, 158°32'0"E, 300–323 m, 17 Oct 2005: 1 male 6.0 mm (USNM 1442022).

*Chesterfield Islands, Coral Sea*: MUSORSTOM 5, NO *Coriolis*: Lord Howe Ridge, Capel Bank, sta CP 269, 24°47'S, 159°37'E, 250–270 m, 9 Oct 1986: 4 males 2.5–3.3 mm, 1 ovig female 3.1 mm (MNHN-IU-2014–9409); Lord Howe Ridge, Capel Bank, sta DW 260, 25°29'S, 159°44'E, 285 m, 8 Oct 1986: 1 male 3.6 mm (MNHN-IU-2014–9407); Lord Howe Ridge, Capel Bank, sta DW 274, 24°45'S, 159°41'E, 285 m, 9 Oct 1986: 1 female 2.2 mm (MNHN-IU-2014–9408); Lord Howe Ridge, Capel Bank, sta CP 275, 24°46.60'S, 150°40.30'E, 285 m, 9 Oct 1986: 1 male 3.7 mm (USNM 1441989); Lord Howe Ridge, Argo Bank, sta DC 291, 23°07.70'S, 159°28.40'E, 300 m, 11 Oct 1986:1 male 3.9 mm (USNM 1442009); Lord Howe Ridge, Nova Bank, sta CP 312, 22°17'S, 159°25'E, 315–320 m, 12 Oct 1986: 1 male 2.8 mm (MNHN-IU-2014–9406).

##### Description.

*Shield* (Figs 18C, 23A) 0.9 to 1.1 longer than broad; dorsal surface glabrous or with scattered setae on sloping lateral surfaces; anterior margins between rostrum and lateral projections concave; posterior margin broadly rounded; lateroventral distal angle produced into small blunt spine-like projection (often with 2 minute terminal tubercles) adjacent to proximal margin of first antennal segment. Rostrum roundly subtriangular, relatively broad, weakly arched and curved ventrally, reaching to distal margin of ocular acicles; with rounded and glabrous dorsal longitudinal ridge. Lateral projections each terminating in short vertical keel-like ridge with 2 or 3 small blunt spines. Gastric region weakly elevated anteriorly. Branchiostegite (Fig. 23B) with anterodorsal plate unarmed or with small blunt distal spine; distal margin setose.

*Ocular peduncles* strongly broadened distally, ca. 0.5 length of shield; corneas strongly dilated, diameter ca. 0.8 of total peduncular length (including the cornea). Ocular acicles small, obtusely triangular, armed with minute subterminal blunt spine directed anterodorsally.

*Antennular peduncles* when fully extended overreaching distal margins of corneas by entire or nearly entire length of ultimate peduncular segments. Ultimate and penultimate segments glabrous or at most with scattered short setae. Basal segment with lateral face having distal subrectangular lobe, minute medial spine, and setose lobe proximally.

*Antennal peduncles* reaching nearly to distal corneal margins. Fifth segment slender, glabrous or with scattered setae. Fourth segment with scattered setae. Third segment with short ventrodistal spine. Second segment with dorsolateral distal angle not noticeably produced, terminating in short spine; mesial margin rounded, setose, dorsomesial distal angle blunt, unarmed. First segment (Fig. 23A) hardly visible in dorsal view or hidden by shield, unarmed. Antennal acicle short, only reaching to distal margin of fourth peduncular segment or mid-point of ocular peduncle, unarmed, terminating bluntly and with few short distal setae. Antennal flagellum short, delicate, not exceeding distal margin of chelae, with few short setae and 1 or 2 long setae every 4–6 flagellar articles.

*Mouthparts*. Mandible with stout palp. Maxillule with recurved external lobe of endopod nearly as long as entire endopod. Maxilla with endopod not exceeding distal end of scaphognathite. Maxilliped 1 with endopod bent medially nearly at right angle, reaching distal end of exopod; epipod elongated. Maxilliped 2 without distinguishing characters. Maxilliped 3 (Fig. 23C) with exopod ca. 2.4 times as long as broad; merus with 3–5 small spines on ventral margin, and usually 2 small spines on ventromesial distal angle; ischium having crista dentata armed with 15–18 small subequal (except for larger distal and proximal) corneous-tipped teeth; basis with row of small spines on mesial margin; coxa with ventromesial angle strongly produced ventrally, with 2–4 small spines and fringe of setae. Sternite VIII narrow, with small setose lobe on each side of midline.

*Chelipeds* (Figs 18C, 23D, E) subequal, similar in armament and setation; dorsal surfaces of chelae and carpi with weakly dense short setation mostly arranged in tufts; ventral surfaces of palms smooth except for scattered setae or tufts of setae. Dactyl and fixed finger with narrow hiatus proximally when closed, forming spoon-like shape in ventral view when closed; each terminating in small curved corneous claw and subdistal blunt calcareous tooth ventral to claw, both claws and teeth interlocking when fingers closed; cutting edge of dactyl with terminal row of small, fused corneous teeth on distal one-third, and row of unequal calcareous teeth on proximal two-thirds; cutting edge of fixed finger with row of blunt calcareous teeth decreasing in size distally. Dactyl as long as palm; dorsal surface convex, weakly pitted and mostly unarmed except for short setae; mesial margin rounded, with few small tubercles; ventromesial face concave. Palm as long as carpus, dorsal surface with scattered small tubercles on dorsolateral and dorsomesial margins, mostly unarmed medially, usually with dense patch of short plumose setae medially near base of fixed finger; dorsolateral margin rounded, not delimited, dorsomesial margin with row of strong spines. Carpus ca. 0.6 times length of merus; dorsal and dorsolateral surfaces with scattered small spines or tubercles; dorsomesial margin with row of weak to moderately strong spines or tubercles, and small blunt distal spine; dorsolateral margin rounded; mesial surface smooth, unarmed except for setae on distal margin; ventral surface smooth except for row of setae on distal margin. Merus nearly as long as chela, subtriangular in cross-section; dorsal margin with row of low protuberances accompanied by tufts of short setae, ventromesial and ventrolateral margins each with irregular row of weak spines or tubercles with setae; lateral and mesial surfaces unarmed except for scattered short setae. Ischium with lateral surface rounded, unarmed, ventromesial margin with row of small spines. Basis with ventromesial row of setae. Coxa with well-marked longitudinal fissure (Fig. 23F) on ventral surface.

*Pereopods 2 and 3* (Fig. 24A–D) slender, similar in armature and setation, slightly dissimilar in length, with pereopod 2 shorter than pereopod 3. Dactyls ca. 1.4 (pereopod 2) or 1.7 (pereopod 3) times as long as propodi, mostly straight in lateral view except for weak distal curvature, terminating in sharp corneous claw; dorsal and ventral margins each with moderately dense simple setae or tufts of setae (some on dorsal margin occasionally bristle-like); ventromesial margins each with 2 or 3 obscure, minute corneous spinules distally; dactyl of pereopod 3 slender, nearly straight in lateral view, 1.1 times as long as dactyl of second pereopod. Propodi ca. 1.2 times as long as carpi; dorsal margin mostly with tufts of long setae, ventral margin with long simple setae or tufts of setae, lateral and mesial faces with scattered short setae. Carpi unarmed except for tufts of setae dorsally and scattered setae ventrally, dorsodistal angle blunt or with obscure small tubercle. Meri unarmed except for fringe of long setae ventrally. Ischia unarmed except for scattered short setae. Coxae of pereopods 3 (Fig. 21D) widely separated by full ventral length of 1 coxa, with few ventromesial setae. Sternite XI (between pereopods 3; Fig. 21D) with undivided anterior lobe consisting of narrow rod-like plate 8 times as broad as long; posterior lobes wider than long, glabrous.

*Pereopod 4* (Figs 21E, 24E, F) with chela club-like, almost 1.1 times as long as carpus and 2.4–3.1 as long as high; palm 1.7–2.2 as long as high. Dactyl strongly curved, hook-like, crossing fixed finger at tip when fingers closed, terminating in distal or subdistal sharp corneous claw; cutting edge unarmed or rarely with few minutely obscure corneous spinules. Fixed finger broad, bulging ventrally at base, glabrous, terminating in sharp corneous claw; cutting edge with 1 distinct sharp corneous-tipped spine (often slightly offset laterally from cutting edge). Palm and carpus with long simple setae or tufts of setae on dorsal margins. Sternite XII (between pereopods 4; Fig. 21D) with fringe of setae more dense laterally than medially.

*Pereopod 5* (Fig. 24G) with chela nearly 0.7 times as long as merus, with long, brush-like setae on dorsal and ventral surfaces. Dactyl with propodal rasp on ventral face. Propodal rasp consisting of minute ovate scales extending for ca. 0.1 length of propodus. Ischium with setae dorsally and ventrally. Coxa with ventrodistal setae.

*Male* gonopod 1 with inferior lamella armed on distal margin with posterior row of slender, semitransparent hook-like spines, and 1 or 2 irregular rows of small straight or slightly curved corneous spines. Gonopod 2 with distal segment strongly twisted distally, densely setose. Left unpaired pleopods 3–5 reduced when present, pleopod 3 biramous, pleopods 4 and 5 uniramous; no pleopods 3–5 on right side (see “Variations”).

*Female* with left unpaired, well-developed, biramous pleopods 2–4 (ovigerous), rarely with vestigial pleopod 5; lacking or rarely having unpaired pleopods 2–5 on right side (see “Variations”). Brood pouch large, oblong, distal margin weakly scalloped and fringed with sparse short setae.

*Uropodal exopods* (Fig. 23G) slender, broadly curved, terminating in strong, usually corneous-tipped spine, anterior margin with fringe of long well-spaced setae and row of well- spaced corneous-tipped spines; endopods relatively short, curved, anterior margin with long setae and 1 or 2 irregular rows of corneous-tipped spines; protopods with strong, curved proximal spine.

*Telson* (Fig. 23G) subrectangular, broader than long; posterior lobes separated by shallow median cleft, terminal margins unarmed except for fringe of long setae.

##### Genetic data.

See Table 1.

##### Color

(Fig. 18C). Shield light orange-red except for white anterior margins. Ocular acicles light orange with white-tipped distal spine and small reddish spot mesially. Antennular and antennal peduncles light orange fading to transparent on distal segments. Ocular peduncles light orange proximally, whitish distally except for orange-red median portion of optic calathus; with dark orange-red band medially; corneas black except for somewhat yellowish external membrane. Chelipeds with carpus and chela with mostly light orange to red background, and white spines or tubercles; dactyl white except for light red portion medially, and small orange spot basally; fixed finger white except for small orange spot mesially at base of larger teeth of cutting edge, and orange portion basally, white coloration continued posteriorly on most of lateral face of palm; carpus with small white portion basally and distally; merus orange mottled with white, with small dorsodistal, laterodistal and mesiodistal dark orange or reddish spot. Pereopods 2 and 3 with dactyls semi-transparent except for median and basal red bands; carpi and meri mottled with semi-transparent white and light orange-red spots or blotches; meri with light orange background and mottled with white and red spots. Pereopod 4 light with dactyl light orange with white tip; chela mostly white except for light orange dorsal margin; carpus mostly white with light orange distal, ventral and proximal margins; merus white with light orange band medially. Pereopod 5 light orange.

##### Etymology.

The species name derives from the Latin *pistillum*, a club-shaped pounder used in a mortar, and refers to the characteristic shape of the chela of pereopod 4.

##### Distribution.

Western Pacific: from the Philippines, Indonesia (Arafura Sea), Solomon Islands, and New Caledonia. Depth: 135 to 849 m.

##### Habitat and symbiont.

Found with undetermined species of acontiate anemone (see “Remarks” under genus *Paguropsis*).

##### Variations.

Of the 152 males examined, 133 (87.5%) have unpaired pleopods 3–5 (reduced, uni- or biramous) on the left side, and the remaining lack unpaired pleopods on either side. Of the 166 females examined, 99% have unpaired pleopods 3–5 on the left side.

##### Remarks.

This new species and *Paguropsina inermis* gen. et sp. n. are very similar. However, the two can be immediately separated by the difference in armature of the cutting edge of the fixed finger on the chela of pereopod 4. In *P. pistillata* gen. et sp. n. the cutting edge is armed with one distinct, sharp corneous-tipped spine that is often slightly offset laterally from the cutting edge, whereas the cutting edge in *P. inermis* gen. et sp. n. is unarmed. Coloration also differs in these two congeners (Fig. 18C, D), primarily on the pattern of the chelipeds (pereopod 1) and pereopods 2 and 3. The chela is orange to red with white spines, and the fixed finger and distolateral half of the palm are white in *P. pistillata* gen. et sp. n., whereas the chela is orange with irregular pattern and shapes of small white spots and white spines in *P. inermis* gen. et sp. n. Pereopods 2 and 3 have dactyls that are semi-transparent except for median and basal red bands, and the propodi, carpi and meri are mottled with semi-transparent white and light orange-red spots or blotches in *P. pistillata* gen. et sp. n., whereas pereopods 2 and 3 are light to dark orange or reddish mixed with irregularly-shaped white areas, the dactyls are white distally and proximally, and red medially, and propodi, carpi and meri are white distally with a small red spot distolaterally in *P. inermis* gen. et sp. n.

#### Paguropsina inermis

Taxon classificationAnimaliaDecapodaDiogenidae

gen. et sp. n.

http://zoobank.org/9DCB897F-C251-448D-9528-A9559F88AE08

**Figure 25.**
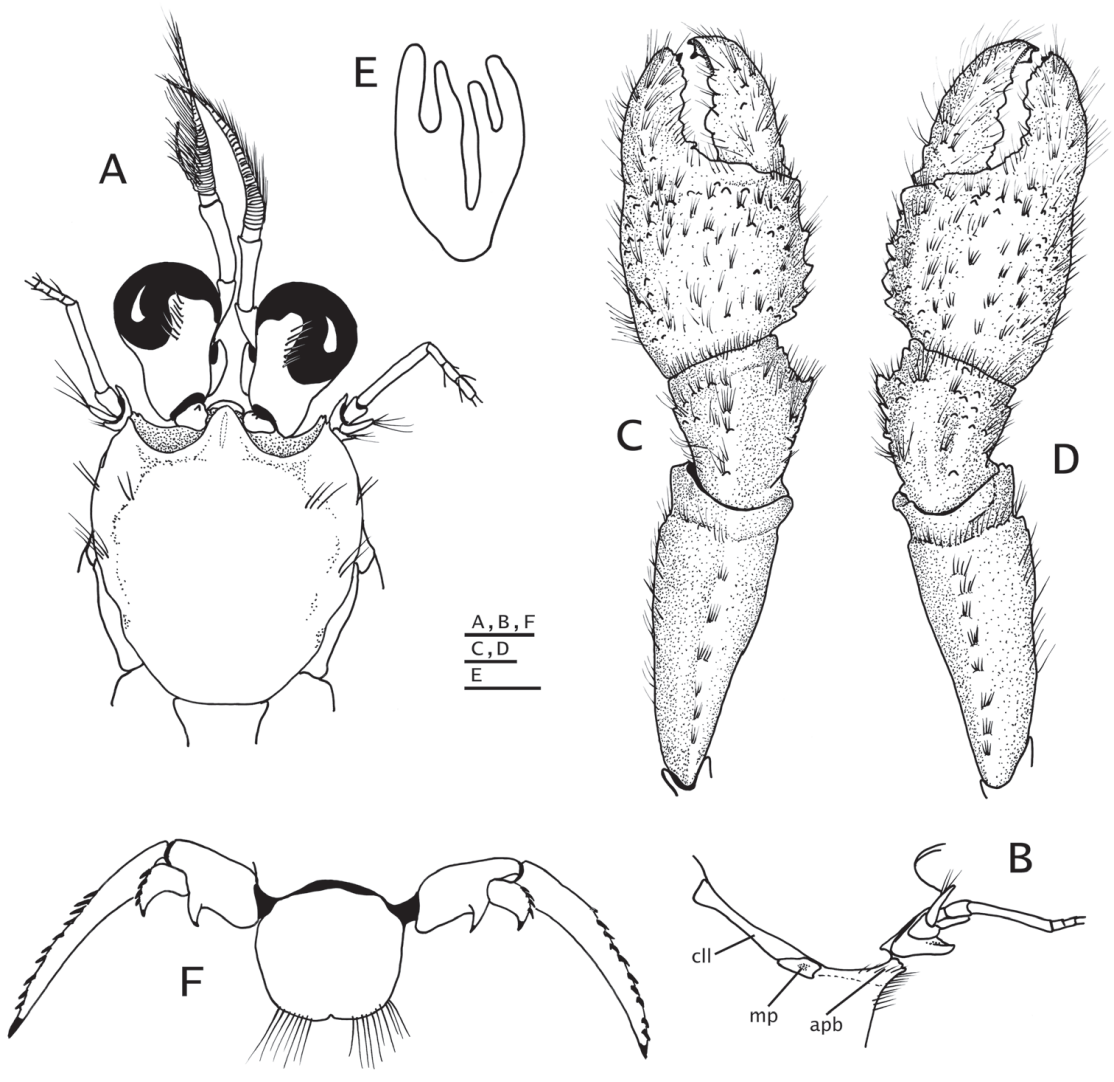
*Paguropsina inermis* gen. et sp. n., holotype male 3.7 mm, New Caledonia, NORFOLK 2, sta CP2119, (MNHN-IU-2014–9386): **A** shield and cephalic appendages **B** right antennal peduncle, branchiostegite, and anterodorsal portion of posterior carapace, lateral **C** left cheliped, dorsal **D** right cheliped, dorsal **E** gill lamella **F** uropods and telson, dorsal. Abbreviations as in Fig. 20. Scale bars: 1 mm (**A–D**), 0.2 mm (**E**), 0.5 mm (**F**).

**Figure 26.**
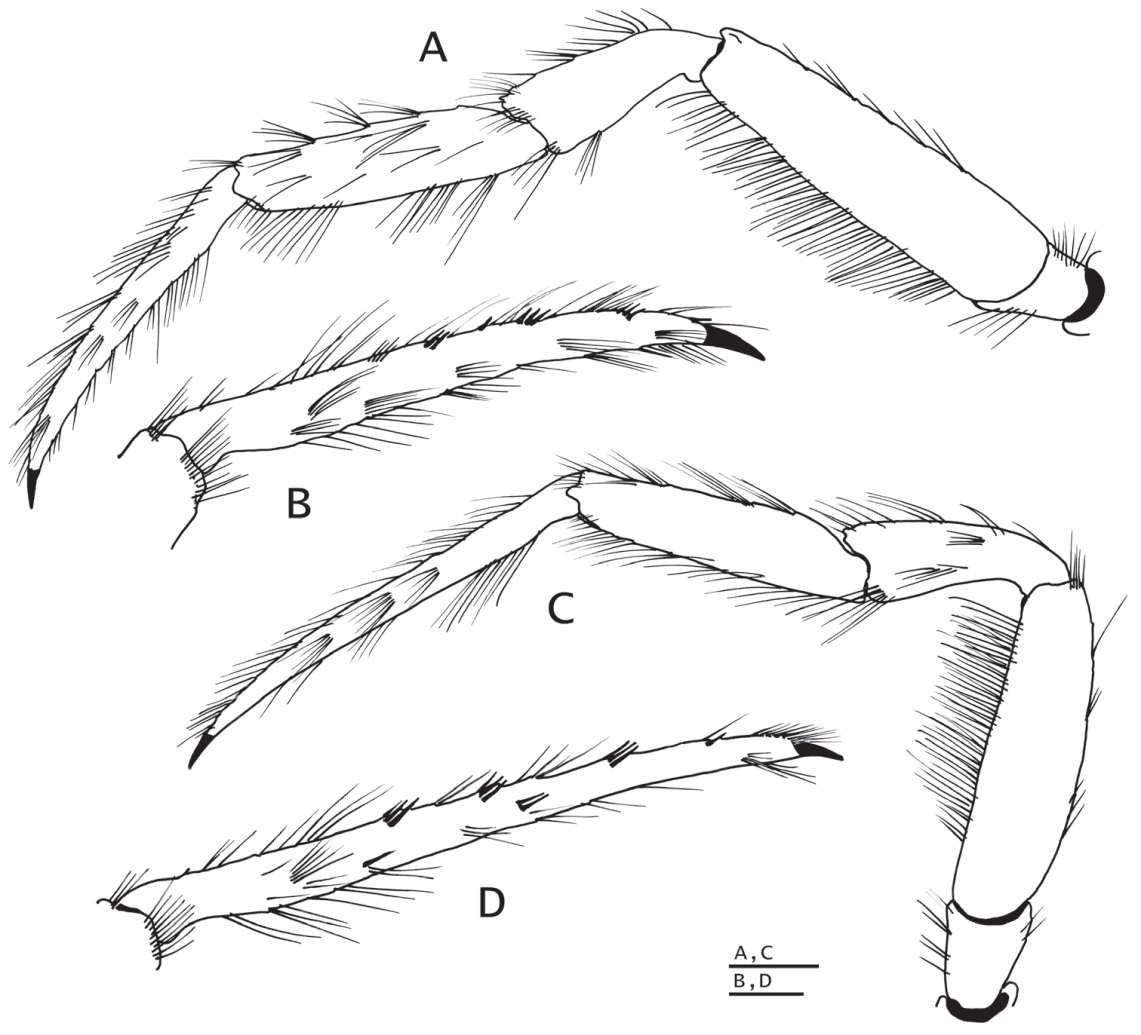
*Paguropsina inermis* gen. et sp. n., holotype male 3.7 mm, New Caledonia, NORFOLK 2, sta CP2119, (MNHN-IU-2014–9386): **A** left pereopod 2, lateral **B** dactyl of same, mesial **C** left pereopod 3, lateral **D** dactyl of same, mesial. Scale bars: 1 mm (**A, C**), 0.5 mm (**B, D**).

**Figure 27.**
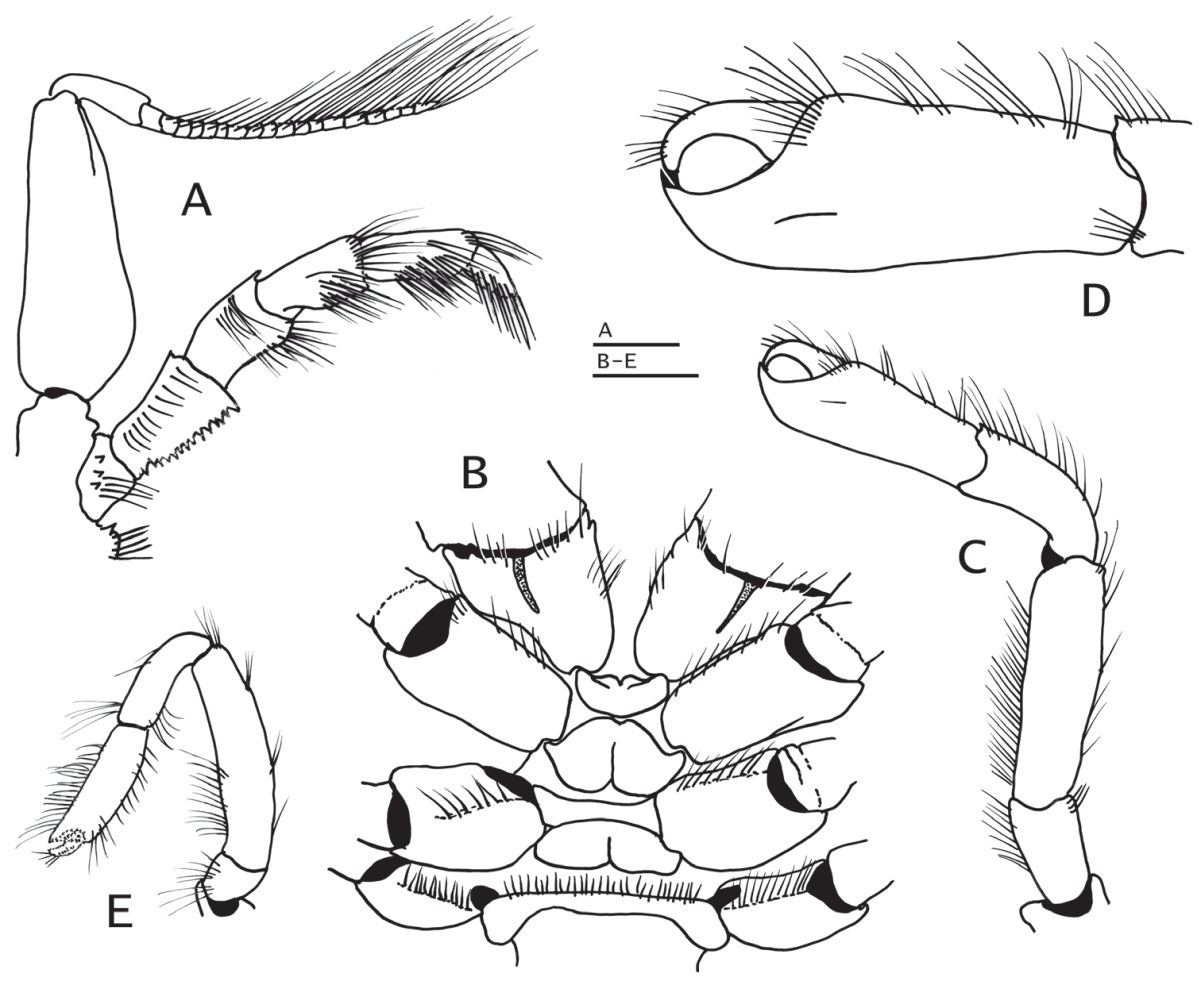
*Paguropsina inermis* gen. et sp. n., holotype male 3.7 mm, New Caledonia, NORFOLK 2, sta CP2119, (MNHN-IU-2014–9386): **A** left maxilliped 3, internal **B** coxae of pereopods 1–4, and sternites IX–XII **C** left pereopod 4, lateral **D** chela of same, lateral **E** left pereopod 5, lateral. Scale bars: 0.5 mm (**A, D**), 1 mm (**B, C, E**).

Figs 18D , 25 , 26 , 27 , Table 1

##### Type material.

Holotype: male 3.7 mm, New Caledonia, NORFOLK 2, Antigonia Bank, sta CP 2119, 23°23'S, 168°02'E, 300 m, 1 Nov 2003 (MNHN-IU-2014–9386).

##### Paratypes.

*Japan*: Ogasawara Islands: TRV *Shin’yo-maru* 1997 research cruise, sta 17: off Chichi-jima Island, 27°24.58'N, 142°10.21'E, 210–212 m, 16 Oct 1997, coll. T Komai: 1 ovig female 2.9 mm (CBM-ZC 14204). RV *Koyo*, 2009 research cruise, sta 21: NW of Otouto-jima Island, 27°13.09'N, 142°09.19'E, 136 m, 15 Jul 2009, coll. T Komai: 1 ovig female 3.8 mm (CBM-ZC 14205); sta 28, E of Nishi-jima Island, 27°07.05'N, 142°10.68'E, 52 m, 15 Jul 2009, coll. T Komai: 1 female 2.5 mm, color photograph (Fig. 18D) (CBM-ZC 14206); sta 34, W of Minami-jima Island, 27°02.34'N, 142°07.52'E, 139–140 m, coll. T Komai: 1 female 2.6 mm (CBM-ZC 14207).

*South China Sea*: ZHONGSHA 2015, ORI cruise 1113: sta CP 4160, 20°48.88'N, 116°43.153'E, 251 m, 30 Jul 2015: 1 ovig female 4.5 mm (NTOU A01447).

*Philippines*: MUSORSTOM 1, NO *Vauban*: N Lubang, sta CP63, 14°00'N, 120°16'E, 191–195 m, 27 Mar 1976: 1 female 6.1 mm (MNHN-IU-2014–9372). PANGLAO 2004: W of Pamilacan Island, Cervera shoal, sand on echinoderms bed, sta T37, 09°28'N, 123°51'E, 134–190 m, 4 Jul 2004: 1 ovig female 4.2 mm (LKCNHM ZRC 2018.0172). PANGLAO 2005, NO “*DA-BFAR*”: Bohol Sea, off Balicasag Island, sta DW 2402, 09°31'N, 123°42'E, 101–118 m, rock/sand/corals, 31 May 2005: 3 males 2.7–3.9 mm (LKCNHM ZRC 2018.0173).

*Indonesia*: KARUBAR, NO *Baruna Jaya 1*: Kai Islands, sta DW18, 05°18'S, 133°01'E, 205–212 m, 24 Oct 1991: 2 males 3.1, 3.2 mm, 1 female 3.5 mm (MNHN-IU-2014–9380).

*Fiji Islands*: BORDAU 1, NO *Alis*: Lau Ridge, Yangasa Cluster, sta DW1497, 18°44'S, 178°25'W, 335–350 m, 12 Mar 1999: 1 male 2.5 mm, 1 female 3.0 mm (MNHN-IU-2013–19456).

*Tonga Islands*: BORDAU 2, NO *Alis*: Vava’u group, sta DW1583, 18°37'S, 174°03'W, 327–360 m, 13 Jun 2000: 1 female 3.0 mm (MNHN-IU-2013–19457).

*New Caledonia*: BATHUS 3, NO *Alis*: Norfolk Ridge, W of Mont Jumeau, sta CP 805, 23°41'S, 168°01'E, 278–310 m, 27 Nov 1993: 1 male 3.4 mm (MNHN-IU-2014–9378). LITHIST, NO *Alis*: Norfolk Ridge, W of Jumeau Bank, sta CP 17, 23°41'S, 168°01'E, 247–281 m, 12 Aug 1999: 1 male 5.7 mm (MNHN-IU-2014–9379). NORFOLK 1, NO *Alis*: Norfolk Ridge, Brachiopode Bank, sta DW 1651, 23°26'S, 167°50'E, 276–350 m, 19 Jun 2001: 4 males 3.1–4.6 mm (MNHN-IU-2014–9352), 2 females 2.8, 3.6 mm (MNHN-IU-2014–9362); Norfolk Ridge, Brachiopode Bank, sta DW 1652, 23°27'S, 167°51'E, 290–378 m, 19 Jun 2001: 1 male 4.6 mm (MNHN-IU-2014–9353); Norfolk Ridge, Brachiopode Bank, sta DW1653, 23°26'S, 167°51'E, 328–340 m, 19 Jun 2001: 4 males 3.6–4.8 mm, 1 female 3.7 mm (MNHN-IU-2014–9355), 1 male 4.2 mm, 1 3.6 mm (MNHN-IU-2014–9381); Norfolk Ridge, Brachiopode Bank, sta DW 1657, 23°26'S, 167°50'E, 305–332 m, 19 Jun 2001: 1 female 4.8 mm (USNM 1441977); Norfolk Ridge, Brachiopode Bank, sta DW 1658, 23°27'S, 167°50'E, 320–336 m, 19 Jun 200: 1 female 3.4 mm (MNHN-IU-2013–19455); Norfolk Ridge, W of Jumeau Bank, sta CP 1669, 23°41'S, 168°01'E, 302–325 m, 21 Jun 2001: 1 male 4.3 mm, 1 female 3.9 mm (MNHN-IU-2014–9351); Norfolk Ridge, W of Jumeau Bank, sta CP1671, 23°42'S, 168°01'E, 320–397 m, 21 Jun 2001: 1 male 5.1 mm (MNHN-IU-2014–9359); Norfolk Ridge, Kaimon-Maru Bank, sta CP 1682, 24°42'S, 168°09'E, 331–379 m, 22 Jun 2001: 1 male 4.5 mm (MNHN-IU-2014–9357); Norfolk Ridge, Kaimon-Maru Bank, sta CP1683, 24°44'S, 168°07'E, 248–272 m, 22 Jun 2001: 1 male 4.5 mm (USNM 1441892); Norfolk Ridge, Crypthelia Bank, sta DW 1724, 23°19'S, 168°15'E, 200–291 m, 27 Jun 2001: 1 female 3.0 mm (MNHN-IU-2014–9358). NORFOLK 2, NO *Alis*: Brachiopode Bank, sta DW 2023, 23°27'S, 167°51'E, 282–297 m, 21 Oct 2003: 1 male 4.3 mm (MNHN-IU-2014–9388); Brachiopode Bank, sta DW 2024, 23°28'S, 167°51'E, 370–371 m, 21 Oct 2003: 1 ovig female 4.5 mm (MNHN-IU-2014–9383); Kaimon-Maru Bank, sta CP 2094, 24°44'S, 168°10'E, 286–300 m, 29 Oct 2003: 1 ovig female 3.9 mm (MNHN-IU-2014–9382), 1 male 4.3 mm (MNHN-IU-2014–9385); Antigonia Bank, sta CP 2119, 23°23'S, 168°02'E, 300 m, 1 Nov 2003: 2 ovig females 3.7, 3.8 mm (USNM 1441978); Crypthelia Bank, sta DW 2124, 23°18'S, 168°15'E, 260–270 m, 2 Nov 2003: 1 ovig female 3.6 mm (MNHN-IU-2014–9390).

##### Description.

*Shield* (Figs 18D, 25A) subovate, about as long as broad; dorsal surface glabrous except for scattered setae on sloping lateral surfaces; anterior margins between rostrum and lateral projections concave; posterior margin broadly rounded; lateroventral distal angle produced into small blunt spine (often with 2 minute terminal tubercles) adjacent to proximal margin of first antennal segment. Rostrum roundly subtriangular, relatively broad, weakly arched and curved ventrally, reaching to distal margin of ocular acicles; with rounded, glabrous dorsomedian longitudinal ridge. Lateral projections each terminating in short vertical keel-like ridge with 2 or 3 small blunt spines distally. Gastric region weakly elevated anteriorly. Branchiostegite (Fig. 25B) with anterodorsal plate unarmed except for 1–3 small blunt distal spines, and setose distal margin.

*Ocular peduncles* strongly broadened distally, ca. 0.5 length of shield; corneas strongly dilated, diameter ca. 0.7 of total peduncular length (including the cornea). Ocular acicles small, obtusely triangular, armed with minute subterminal blunt spine directed anterodorsally.

*Antennular peduncles* when fully extended overreaching distal margins of corneas by entire or nearly entire length of ultimate peduncular segments; ultimate and penultimate segments glabrous or at most with scattered short setae; basal segment with lateral face having distal subrectangular lobe, minute medial spine, and setose lobe proximally.

*Antennal peduncles* reaching nearly to distal margin of corneas. Fifth segment slender, glabrous or with scattered setae. Fourth segment with few scattered setae. Third segment with short ventrodistal spine. Second segment with dorsolateral distal angle not noticeably produced, terminating in short spine; mesial margin rounded, setose, dorsomesial distal angle blunt, unarmed. First segment (Fig. 25A) hardly visible in dorsal view or hidden by shield, unarmed. Antennal acicle short, only reaching to distal margin of fourth peduncular segment or mid-point of ocular peduncle, unarmed, terminating bluntly and with few short distal setae. Antennal flagellum short, delicate, not exceeding distal margin of chelae, with few setae 1 to 2 flagellar articles in length.

*Mouthparts* similar to those described for the type species *Paguropsis pistillata* sp. n. Maxilliped 3 (Fig. 27A) with exopod 2.7 times as long as broad.

*Chelipeds* (Figs 18D, 25C, D) subequal, similar in armament and setation; dorsal surfaces of chelae and carpi with weakly dense short setation mostly arranged in tufts; ventral surfaces of palms smooth except for scattered setae or tufts of setae. Dactyl and fixed finger with narrow hiatus proximally when closed, forming spoon-like shape in ventral view when closed; each terminating in small curved corneous claw and subdistal blunt calcareous tooth ventral to claw, both claws and teeth interlocking when fingers closed; cutting edge of dactyl with terminal row of small, fused corneous teeth on distal one-third, and row of unequal, strong calcareous teeth on proximal two-thirds; cutting edge of fixed finger with row of blunt, irregular calcareous teeth on proximal two-thirds, and row of partially fused, subequal, small calcareous teeth on distal third. Dactyl as long as palm; dorsal surface convex, weakly pitted and mostly unarmed except for scattered low tubercles with short setae; mesial margin rounded, with few small tubercles; ventromesial face concave. Palm as long as carpus, dorsal surface with scattered small tubercles on dorsolateral and dorsomesial margins, mostly unarmed medially except for well-spaced tufts of short to moderately long setae; dorsolateral margin rounded, not delimited, dorsomesial margin with row of strong spines. Carpus ca. 0.6 times length of merus; dorsal and dorsolateral surfaces with scattered small spines or tubercles; dorsomesial margin with row of weak to moderately strong spines or tubercles, and small blunt distal spine; dorsolateral margin rounded; mesial surface smooth, unarmed except for setae on distal margin; ventral surface smooth except for few long setae on distal margin. Merus slightly shorter to nearly as long as chela, subtriangular in cross-section; dorsal margin with row of low protuberances accompanied by tufts of short setae, ventromesial and ventrolateral margins each with irregular row of weak spines or tubercles with setae; lateral and mesial surfaces unarmed except for scattered short setae. Ischium with lateral surface rounded, unarmed, and row of small spines on ventromesial margin. Basis with ventromesial row of setae. Coxa with well-marked longitudinal fissure (Fig. 27B) on ventral surface.

*Pereopods 2 and 3* (Fig. 26A–D) similar in armature and setation, slightly dissimilar in length, with pereopod 2 shorter than pereopod 3. Dactyls ca. 1.5 (pereopod 2) or 1.8 (pereopod 3) times as long as propodi, mostly straight in lateral view except for weak distal curvature, terminating in sharp corneous claw; dorsal margins each with moderately dense, long simple setae; ventral margins each variably armed, with row of short or obscure spines, or with row of long, slender corneous spines; dorsomesial margins and mesial faces each usually with several tufts or short transverse rows of long, slender corneous spines (more numerous on pereopod 3); dactyl of pereopod 3 slender, ca. 1.2 times as long as dactyl of second pereopod. Propodi ca. 1.1 times as long as carpi; dorsal margin with few tufts of long setae, ventral margin with long simple setae or tufts of setae, lateral and mesial faces with scattered short setae. Carpi unarmed except for tufts of setae dorsally and scattered setae ventrally, dorsodistal angle with small blunt spine or obscure small tubercle. Meri unarmed except for few long setae dorsally and fringe of long setae ventrally. Ischia unarmed except for scattered short setae. Coxae of pereopods 3 (Fig. 27B) widely separated by ventral length of 1 coxa, with few ventromesial setae. Sternite XI (between pereopods 3; Fig. 27B) with undivided anterior lobe consisting of narrow rod-like plate 8–10 times as broad as long.

*Pereopod 4* (Fig. 27C, D) with chela club-like, ca. 1.3 times as long as carpus and ca. 2.5 times as long as high; palm ca. 1.7 times as long as high. Dactyl strongly curved, hook-like, crossing fixed finger at tip when fingers closed, terminating in distal or subdistal sharp corneous claw; dorsal margin with scattered setae distally; cutting edge unarmed. Fixed finger broad basally, slightly bulging at base, glabrous, terminating in sharp corneous claw; cutting edge unarmed. Palm smooth, unarmed except for few setae on dorsal margin and distolateral margin next to base of dactyl. Sternite XII (between pereopods 4; Fig. 27B) with transverse fringe of setae all across.

*Pereopod 5* (Fig. 27E) with chela 0.7 times as long as merus, with long, brush-like setae on dorsal and ventral surfaces. Dactyl with propodal rasp on ventral face. Propodal rasp consisting of minute ovate scales extending for 0.1 length of propodus. Ischium with setae dorsally and ventrally. Coxa with ventrodistal setae.

*Male* gonopod 1 with inferior lamella armed on distal margin with posterior row of slender, semitransparent hook-like, corneous spines. Gonopod 2 with distal segment strongly twisted distally, densely setose. Left unpaired pleopods 3–5 reduced when present (see Variations); when present, pleopod 3 biramous, pleopods 4 and 5 uniramous; no pleopods 3–5 on right side.

*Female* with left unpaired, well-developed, biramous pleopods 2–4 (ovigerous), rarely with vestigial pleopod 5; usually without unpaired pleopods 2–5 on right side (see “Variations”). Brood pouch large, oblong, distal margin weakly scalloped and fringed with sparse short setae.

*Uropodal exopods* (Fig. 25F) slender, broadly curved, terminating in strong, usually corneous-tipped spine, anterior margin with fringe of long, well-spaced setae and row of well-spaced corneous-tipped spines; endopods relatively short, curved, anterior margin with long setae and 1 or 2 irregular rows of corneous-tipped spines; protopods with strong, curved proximal spine.

*Telson* (Fig. 25F) subrectangular, slightly broader than long; posterior lobes usually broadly rounded, separated by shallow median cleft, terminal margins unarmed except for fringe of long setae.

##### Genetic data.

See Table 1.

##### Color

(Fig. 18D). Shield overall light orange overall, with darker orange and pair of white spots marking anterior margin of gastric region. Ocular acicles white. Ocular peduncles whitish basally, light orange distally, and with dark orange portion distolaterally and on optic calathus; corneas black except for somewhat yellowish external membrane. Antennular and antennal peduncles white to light orange. Chelipeds with meri, carpi, and chelae orange with irregular pattern and shapes of small white spots, and white spines; dactyls each with red spot basally; carpi each with red spot distodorsally; meri each with small red spots, one on dorsal margin distally, and three on distal margin. Pereopods 2 and 3 light to dark orange or reddish mixed with irregularly shaped white areas; dactyls white on distal one-third; carpi and meri each white distally and with small red spot distolaterally. Pereopod 4 with dactyl light orange dorsally, white distally and along cutting edge; palm and fixed finger mostly white except for orange medially on ventral and dorsal margins; carpus white to light orange, with darker orange on dorsal margin; merus distally light orange with darker orange on ventral margin, and white ventroproximally. Pereopod 5 mostly white with some light orange around margins of segments.

##### Etymology.

The species name derives from the Latin *inermis*, meaning unarmed, and refers to the lack of armature on the cutting edge and lateral face of the fixed finger of pereopod 4, the main characteristic setting of this species.

##### Distribution.

Western Pacific: Japan (Ogasawara Islands), South China Sea, Philippines, Indonesia (Banda Sea), Fiji Islands, Tonga Islands, and New Caledonia. Depth: 101 to 397 m.

##### Habitat and symbiont.

Found with indeterminate species of acontiate anemone (see “Remarks” under genus *Paguropsis*).

##### Variations.

As noted above, the armature of the dactyls of pereopods 2 and 3 is noticeably variable among the numerous specimens examined of this new species. The dorsomesial and ventromesial margins vary in armature from having bristle-like setae and obscure or altogether lacking any clearly visible corneous spines (e.g., in the holotype, Fig. 26B, D) to the margins armed with long, slender, corneous spines. When corneous spines are present, they can be arranged in dorsomesial and ventromesial rows of single spines (pereopod 2), or in rows of 1–3 clustered spines (pereopod 3). The presence or strength of the armature of the dactyls do not appear to be related to size of the specimens.

In the 25 males examined, the presence of left unpaired pleopods 3–5 is variable, as follows: 77% have reduced, biramous left pleopod 3; 55% have a reduced, uniramous pleopod 4; and 11% have a reduced, uniramous pleopod 5; 22% lack pleopods 3–5 altogether; no male specimens were found to have pleopods 3–5 on right side. Virtually all 22 females examined had pleopods 2–4 only on the left side and lacked pleopod 5. However, 10% of the females had a uniramous, vestigial pleopod 5. Only one ovigerous female (3.8 mm, CBM-ZC 14205) from Ogasawara Islands, Japan, was found to have right unpaired pleopods 2–4.

##### Remarks.

See *Paguropsina pistillata* gen. et sp. n.

### Key to species of *Paguropsis* and *Paguropsina* gen. n.

**Table:** 

1	Exopod of maxilliped 3 slender, 4 or more times as long as broad (e.g., Fig. 4F); width of sternite XI less than 0.5 length of 1 coxa of pereopod 3 (e.g., Fig. 5B); cutting edge of fixed finger of chela of pereopod 4 armed with sharp spines arranged like bear claw (e.g., Fig. 14); gills with lamellae distally divided into filamentous or stub-like extensions (e.g., Fig. 3A)	***Paguropsis* Henderson, 1888)**...**2**
–	Exopod of maxilliped 3 broad, 2.4 times as long as broad (e.g., Fig. 23C); width of sternite XI same as length of 1 coxa 1 of pereopods 3 (e.g., Fig. 21D); cutting edge of fixed finger of chela of pereopod 4 unarmed or at most with 1 distinct corneous spinule (often slightly offset laterally from cutting edge; Figs 24F, 27D); gills with lamellae deeply divided distally into finger-like extensions (e.g., Fig. 25E)	***Paguropsina* gen. n**....**6**
2	Lateroproximal surface of dactyl of pereopod 3 with longitudinal concavity; sternite XI separating coxae of pereopods 3 by ca. 0.2 length of 1 coxa, posterior lobes of sternite XI strongly compressed	**3**
–	Lateroproximal surface of dactyl of pereopod 3 convex, without longitudinal concavity; sternite XI separating coxae of pereopods 3 by distinctly more than 0.2 length of 1 coxa, posterior lobes of sternite XI not compressed	**4**
3	Lateroproximal surface of dactyl of pereopod 3 with distinct and usually weakly calcified longitudinal concavity (Figs 1E, 10C); background coloration mostly orange-red (Figs 8C, 28B)	***Paguropsis andersoni* (Alcock, 1899)**
–	Lateroproximal surface of dactyl of pereopod 3 with weak, calcified longitudinal concavity; background coloration mostly white with orange patches on pereopods 2 and 3 (Figs 8D, 28C, D)	***Paguropsis confusa* sp. n.**
4	Chela of pereopod 4 lacking dense patch of capsulate setae on dorsal margin of palm (only with fringe of long setae); coloration as in Figs 8A, B, 28A	***Paguropsis typica* Henderson, 1888**
–	Chela of pereopod 4 with dense patch of capsulate setae on dorsal margin of the palm; coloration not as above	**5**
5	Patch of capsulate setae on dorsal margin of palm of chela of pereopod 4 arranged in a series of oblique rows or fringes of setae, patch occupying area from dorsal margin to nearly midlength of lateral face of palm (Fig. 15D); dorsal surfaces of chelae and carpi covered with dense tufts or short rows of long, bristle-like setae nearly obscuring armature below; dactyls of pereopods 2 and 3 relatively wide (7–9 times as long as broad); coloration: Fig. 18A	***Paguropsis gigas* sp. n.**
–	Patch of capsulate setae on dorsal margin of palm of chela of pereopod 4 not arranged in rows or fringes of setae, patch occupying area from dorsal margin to nearly one-fourth of lateral surface of the palm (Fig. 21B, C); dorsal surfaces of chelae and carpi moderately covered with short bristle-like setae not obscuring armature below; dactyls of pereopods 2 and 3 relatively narrow (10–16 times as long as broad); coloration: Fig. 18B	***Paguropsis lacinia* sp. n.**
6	Cutting edge of fixed finger of chela of pereopod 4 armed with 1 distinct corneous-tipped spine often slightly offset laterally from cutting edge (Fig. 24F); coloration: Fig. 18C	***Paguropsina pistillata* gen. et sp. n**
–	Cutting edge of fixed finger of chela of pereopod 4 unarmed (Fig. 27D); coloration: Fig. 18D	***Paguropsina inermis* gen. et sp. n**

## Discussion

### Biogeographic summary

The results of this revision provide a vision of the overall biogeographic distribution of the seven species herein discussed of *Paguropsis* and *Paguropsina* gen. n. This vision, however, is preliminary as it is evident that many of the vast marine shelf areas and deep-sea habitats of the Indo-West Pacific where species of these two genera live, still remain to be sampled. Species of *Paguropsis* are distributed in the tropical and subtropical regions of the Indo-West Pacific, in continental shelf to upper slope depths of 30 to 1125 m. Of the five species of *Paguropsis*, only two have been found to occur outside the western Pacific: *P. andersoni*, distributed widely across the Indian Ocean, from off southeastern Africa, both coasts of India, Andaman Sea, to the extreme southeastern coast of Indonesia in the Arafura Sea; and *P. confusa* gen. et sp. n., found from the southwestern Indian Ocean and the western Pacific from the Philippines and Makassar Straits, Indonesia (Kalimantan). The other three species of *Paguropsis*, *P. typica*, *P. gigas* sp. n., and *P. lacinia* sp. n., have been found to be distributed exclusively in the western Pacific region. Of these, *P. typica* occurs the farthest north (off Daito Islands, Ryukyu Islands, Japan) and the farthest south (off eastern Australia), and reaches the Fiji Islands to the east; *P. gigas* sp. n. is so far known only from the South China Sea; and *P. lacinia* sp. n. has the easternmost distribution in the South Pacific, distributed from the Solomon Islands to the Tonga Islands. The two species of *Paguropsina* gen. n. are exclusively distributed in the western Pacific, in depths ranging from 52 to 849 m. Of these, *P. pistillata* gen. et sp. n. occurs from the Philippines to the New Caledonia region; and *P. inermis* gen. et sp. n. from Ogasawara Islands, Japan and off Taiwan in the South China Sea, to the Tonga Islands. It is noteworthy that despite extensive sampling during the last three decades in French Polynesia (Tuamotu Archipelago), Marquesas, Society and Austral Islands (e.g., Bouchet 2009; Poupin 1991, 1998, 2010; Richer de Forges 2002; Richer de Forges et al. 1999), no species of *Paguropsis* or *Paguropsina* gen. n. have yet been found there.

### Morphology and evolution

The unusual body symmetry and variable presence and position of unpaired pleopods 3–5 in males and unpaired pleopods 2–5 in females, and in the case of females also a brood pouch, indistinctly on the left or right side of *Paguropsis typica*, has been highlighted by various carcinologists since the time of Henderson’s (1888) description of his remarkable species (e.g., Stebbing 1893, Alcock 1905, Boas 1926, Russell 1962). Indeed the striking symmetry of cephalothorax, pereopods (including chelipeds), membranous pleon (unpaired pleopods 2–5 excepted), uropods and telson, is unique among the so-called “asymmetrical” paguroids (i.e., all families except Pylochelidae). The pleonal symmetry and variability of pleopods of *P. typica* was compared by Mayo (1973) with a similar condition found, at least in females, in species of the diogenid *Cancellus* H. Milne Edwards, 1836, which live in pieces of rock, sponge, coral or other firm substrates. As in the species discussed herein of *Paguropsis* and *Paguropsina* gen. n., those of *Cancellus* also have symmetrical pereopods (including chelipeds) uropods and telson. In several important respects, however, species of *Paguropsis* and *Paguropsina* gen. n. differ drastically from *Cancellus*, and it is probable that the symmetry exhibited by species of these genera is not indicative of a close phylogenetic relationship and instead is attributable to parallel evolution. In *Cancellus*, pereopods 4 and 5, and uropods, have strongly developed rasps as in many typical “asymmetrical” paguroids, and pereopod 4 is only semichelate (McLaughlin 2003), whereas in *Paguropsis* and *Paguropsina* gen. n. there are no rasps, and pereopod 4 is fully chelate. Furthermore, males of species of *Cancellus* lack all pleopods, whereas males of species of *Paguropsis* and *Paguropsina* gen. n. have paired pleopods 1 and 2 modified as gonopods, normally have unpaired pleopods 3 and 4, and often also pleopod 5 as well albeit reduced.

Previous to this study, the development of a fully chelate pereopod 4, without a propodal rasp, has been less noted in *Paguropsis*. This condition is only present in one other paguroid, the semi-terrestrial coenobitid *Birgus latro* (Linnaeus, 1767) where the abandonment of housing for protection by adults and the non-aquatic life has led to various unusual morphological feeding and respiratory specializations (Drew et al. 2010). It seems evident that convergence has taken place and thus the development of chelate pereopods 4 is for very different purposes, and has occurred independently in these two genera classified in different families, specifically for handling the carcinoecia (acontiate anemone) in *Paguropsis* and *Paguropsina* gen. n., or as a general prehensile mechanism in *B. latro*. Regrettably, the behavior of species of *Paguropsis* and *Paguropsina* gen. n. has not been studied other than the basic observation at the time of the discovery of *P. typica*, that individuals use the specialized chelae of pereopods 4 to grasp and pull the blanket-like coenosarc of their acontiate anemone symbiont to cover or uncover their bodies (Alcock 1905).

The evolution of the specialized morphology of species of *Paguropsis* and *Paguropsina* gen. n. can be attributed to the symbiosis with anemones as mode of housing, without the intervention of mollusk shells or other hard habitat at any time during the life of the hermit crab. Aside from symmetry, species of *Paguropsis* and *Paguropsina* gen. n. are unique in lacking rasp structures on pereopods and uropods, a character that is a *sine qua non* for all five families of “asymmetrical” Paguroidea. Only two other species among the “asymmetrical” hermit crabs are known, or at least suspected, to use exclusively a cnidarian as carcinoecia, the Parapaguridae *Tylaspis anomala* Henderson, 1885 and *Sympagurus poupini* Lemaitre, 1994 (Lemaitre 1994 1998). However, except for the shape of the uropods and telson, these two parapagurids do have at least vestiges of rasps, are not strictly symmetrical as they have a right cheliped larger than the left. Although paired pleopods are present in *T. anomala*, these are asymmetrical and differ in degree of development from one side to the other.

Aside from the generic characters that differentiate species of *Paguropsis* and *Paguropsina* gen. n., the morphology of the species in these two genera is remarkably homogenous, with species differing only in relatively minor details. This homogeneity can be attributed, at least in part, of the adaptation to a similar symbiotic mode of life with an acontiate anemone as means of protection for the membranous pleon. All species in the two genera live symbiotically with indeterminate species of anemones. The need to manipulate their cnidarian symbiont has evidently led species of *Paguropsis* and *Paguropsina* gen. n. to develop fully chelate pereopods 4, the appendage used to grasp and move back and forth the sheet-like coenosarc of the symbiont. It is striking, however, that two species of *Paguropsis* (*P. gigas* sp. n. and *P. lacinia* sp. n.), and both known species of *Paguropsina* gen. n. (*P. pistillata* gen. et sp. n. and *P. inermis* gen. et sp. n.), have developed specialized morphological features on the chela of this appendage, those of the former genus as setal patches (Figs 15D, 17A, 21B, C, 22E), and those of the latter genus by the presence or absence of a conspicuous spine on the fixed finger (Figs 21E, 24F, 26D).

There is one unusual character discovered during this study that had not been documented before in the single previously known species of *Paguropsis*, *P. typica*: the presence on the ventral face of the coxae of the chelipeds of a decalcified longitudinal fissure (e.g., Fig. 5B, D). The fissure is present in all species of *Paguropsis* and *Paguropsina* gen. n. Although rare in paguroids, a similar and presumably homologous fissure on the coxae of the chelipeds has been documented by Forest (1989, 1993) in species of two other diogenid genera, *Paguristes* Dana, 1851 and *Bathynarius* Forest, 1989, and by Komai & Takeda (2004) in yet another species of the latter genus, *B. izuensis* Komai & Takeda, 2004. The function of this fissure is unknown, although as surmised by Forest (1989, 1993), it most likely plays a role during the molting period.

Modern studies that have focused on details of anomuran evolution (e.g., Tsang et al. 2011, Bracken-Grissom et al. 2013) have reaffirmed the virtually unanimous long held view that asymmetrical paguroids evolved from symmetrical ancestors similar to the “symmetrical” hermit crabs of the family Pylochelidae, and that the asymmetrical features exhibited by most paguroids originated as result of habitation in gastropod shells (particularly dextrally coiled shells). Although these modern studies have not been explicit in defining “asymmetry”, it is understood that the asymmetry refers to the most prominent external asymmetrical paguroid features, i.e., the right or left handedness, coiled pleon with unpaired pleopods, and various degrees of asymmetrical development of uropods and telson. One other external feature unique to all hermit crabs, whether they are symmetrical or asymmetrical, is the presence of rasp structures for better gripping their hard housing, on the propodi of pereopods 4 and 5 and anterior margins of uropods. These rasps structures consist of surfaces typically densely covered by modified setae that are scale-like or conical in shape (Keiler and Richter 2011). However, none of these modern studies have incorporated the previously monotypic *Paguropsis* in their data. That species of *Paguropsis* and *Paguropsina* gen. n. are symmetrical and lack any vestige of rasps in any appendage, seems to indicate three possible evolutionary scenarios: 1) the ancestor of these species did not use shells or for that matter any other form of hard type of housing for pleonal protection, and thus the asymmetry of pleopods requires a different explanation than the use of gastropod shells; 2) use of gastropod shell by the ancestor of these species occurred for a period not long enough to cause or fix any development of paguroid features except for beginnings of asymmetry in pleopods; or 3) the ancestor of these species was asymmetrical in all paguroid features but they secondarily regained their ancestral symmetry in all features except the pleopods. Regardless of which scenario is more plausible, species of *Paguropsis* and *Paguropsina* gen n. appear to represent a clade of key importance in paguroid evolution.

### Preliminary remarks on molecular genetics

The molecular data herein included (Table 1) complements the morphological descriptions or redescriptions of all seven species of *Paguropsis* and *Paguropsina* gen. n. This data is intended for future studies of genetic relationships among populations of the different species or in comprehensive phylogenetic studies of diogenids in particular and paguroids in general. A preliminary maximum likelihood tree generated from a concatenated analysis of three mitochondrial genes (12S, 16S, COI), and using the pagurid *Phimochirus operculatus* (Stimpson, 1859) as outgroup, reveals that all the species herein discussed separate into two highly supported clades, matching the proposed morphologically-based separation into two genera, i.e., *Paguropsis*, with the five species, and *Paguropsina* gen. n., with two species. Thus it seems evident that at least the main morphological characters used to differentiate the two genera, i.e., the shape of gills, shape of exopod of the third maxilliped, width of sternite XI, and armature of the fingers of pereopod 4, are of significant evolutionary value. Within each clade or genus, the species can be distinguished as well, although in some phenotypically similar species-pairs, i.e., *Paguropsis typica*-*P. confusa*, and *Paguropsina pistillata*-*P. inermis*, the separation is minimal or obscure based on our mitochondrial gene dataset. The minimal genetic separation of these species-pairs is not unexpected, given that the morphological differences between them are minimal. A wide character analysis combining morphology and genetic data of *Paguropsis* and *Paguropsina* gen. n., and incorporating other diogenid genera, is needed to better understand the evolution and origin of the family Diogenidae.

### Symbiotic association

**Figure 28.**
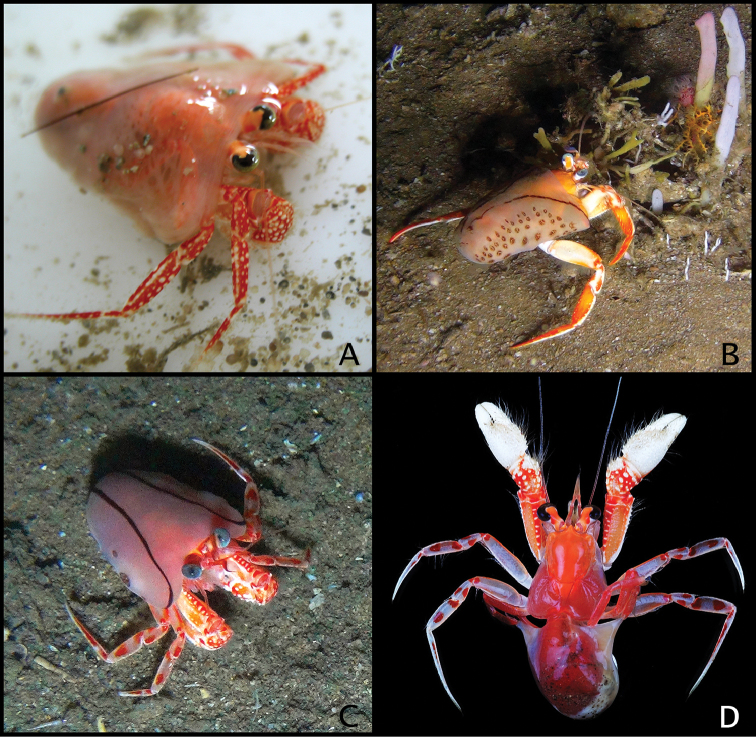
**A** *Paguropsis typica* Henderson, 1888: live specimen in sorting tray on board ship (photograph: B Richer de Forges) **B** *Paguropsis andersoni* (Alcock, 1899), not collected, ROV, KwaZulu-Natal, South Africa, DST/NRF ACEP Spatial Solutions cruise, Aliwal outer reef, off KwaZulu-Natal, RY *Angra Pequena*, sta R50: specimen photographed *in situ* with ROV, not collected (photograph: DST/NRF ACEP – Spatial Solutions project team) **C, D** *Paguropsis confusa* sp. n.: **C** specimen photographed *in situ* with ROV, not collected, off Durban, KwaZulu-Natal, South Africa, DST/NRF ACEP Spatial Solutions Project cruise, sta R45 Echinoderm Extravaganza (photograph: DST/NRF ACEP – Spatial Solutions project team) **D** not examined, Philippines, LUMIWAN CP 2867–16 (photograph: T-Y Chan).

The most remarkable biological feature of species of *Paguropsis* and *Paguropsina* gen. n. is their intriguing symbiotic association with acontiate anemones, without the intervention of snail shells or other hard type of housing. Regrettably, virtually no information exists on the nature and origin of the association except for the brief comments in reports of earlier naturalists (e.g., Alcock 1899, 1905, Boas 1926). Even the identity of the symbiont remains unclear, with earlier authors such as Boas (1926) reporting the cnidarian symbiont used by *Paguropsis typica* to be *Epizoanthus paguropsidis* (see Fig. 1D). However, recent photographs (Fig. 28A–D) of live specimens of *Paguropsis typica, P. andersoni*, and *P. confusa* (of the former species taken on board during an expedition in the Philippines, and of the latter two species taken *in situ* with ROV off South Africa, western Indian Ocean), reveal that the symbionts are undetermined species of acontiate anemones that have puzzling dark spots and lines on their body wall. Whether each species of *Paguropsis* or *Paguropsina* gen n. associate with a particular species of anemone remains unknown. In the interest of documenting additional information on the symbionts, we shared these photographs with anemone expert Dr. DG Fautin (Professor Emerita, University of Kansas), who kindly provided the following explanation (in litt.): “Many acontiate anemones that live on hermit crabs have cinclides (pores; singular cinclis) through which the acontia can be emitted. When the anemone contracts with the mouth tightly closed, the acontia are carried out through the cinclides with the water. Cinclides are typically in a ring distal to (“above”) the base of the anemone but they otherwise resemble these spots in being darker around the edge (which may be raised) than in the middle (through which acontia are emitted). In some anemones the pore is patent; in others, there appears to be no actual pore but the body wall is much thinner in that part than elsewhere – so the wall ruptures when pressure rises, thus acting like safety valves.” Various carcinologists (e.g., Alcock 1905; Boas 1926), based on preserved specimens, have documented and illustrated how *Paguropsis typica* presumably utilizes the prehensile chelae of pereopods 4 to move back and forth the coenosarc of the anemone to cover their bodies up to the shield and partially the meri of pereopods 3 (Figs 1A, E, 28A–C). A photograph of a freshly caught specimen of *P. confusa* sp. n. (Fig. 28D) obtained during this study, provides an indication of how effectively this species can grab onto the thin-walled body of the anemone using the dactyl and bear claw-like spination of the fixed finger of the chelae of pereopods 4. However, the two species of *Paguropsina* gen. n. have developed a slightly different and more simplified grasping morphology on the dactyl and fixed finger of pereopod 4 that resembles ice-block tongs. In these two species, the dactyl and fixed finger are strongly curved distally forming a near circle so that the claws directly face each other, and lack the bear claw-like spination on the fixed finger. In *P. pistillata* gen. et sp. n., the fixed finger is armed with a single spine (Figs 21B, 24F), and in *P. inermis* gen. et sp. n., the fixed finger is unarmed.

## Supplementary Material

XML Treatment for Paguropsis

XML Treatment for Paguropsis typica

XML Treatment for Paguropsis andersoni

XML Treatment for Paguropsis confusa

XML Treatment for Paguropsis gigas

XML Treatment for Paguropsis lacinia

XML Treatment for Paguropsina

XML Treatment for Paguropsina pistillata

XML Treatment for Paguropsina inermis
